# Photooxidation of thiosaccharides mediated by sensitizers in aerobic and environmentally friendly conditions†

**DOI:** 10.1039/d0ra09534f

**Published:** 2021-03-01

**Authors:** Miqueas G. Traverssi, Alicia B. Peñéñory, Oscar Varela, Juan P. Colomer

**Affiliations:** Departamento de Química Orgánica, Universidad Nacional de Córdoba, Facultad Ciencias Químicas, Ciudad Universitaria Edificio de Ciencias II Córdoba Argentina juanpablo@fcq.unc.edu.ar; Instituto de Investigaciones en Fisico-Química de Córdoba (INFIQC), Consejo Nacional de Investigaciones Científicas y Técnicas (CONICET), UNC Argentina; Departamento de Química Orgánica, Universidad de Buenos Aires, Facultad Ciencias Exactas y Naturales, Ciudad Universitaria Pab. 2, C1428EHA Buenos Aires Argentina; Centro de Investigación en Hidratos de Carbono (CIHIDECAR), Consejo Nacional de Investigaciones Científicas y Técnicas (CONICET), UBA Argentina

## Abstract

A series of β-d-glucopyranosyl derivates have been synthesized and evaluated in photooxidation reactions promoted by visible light and mediated by organic dyes under aerobic conditions. Among the different photocatalysts employed, tetra-*O*-acetyl riboflavin afforded chemoselectively the respective sulfoxides, without over-oxidation to sulfones, in good to excellent yields and short reaction times. This new methodology for the preparation of synthetically useful glycosyl sulfoxides constitutes a catalytic, efficient, economical, and environmentally friendly oxidation process not reported so far for carbohydrates.

## Abstract 2

An environmentally friendly and simple sensitized photooxidation methodology to obtain glycosyl sulfoxides with outstanding chemoselectivity in aerobic conditions is described.

## Graphical abstract

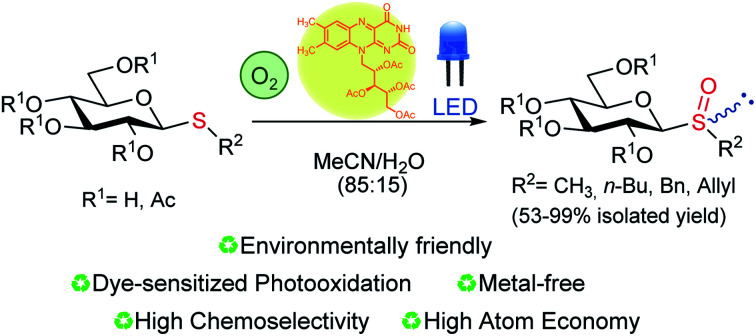

## Introduction

Sulfoxides play important roles as organic synthetic intermediates^1^ and biologically active compounds employed in the pharmaceutical industry.^2^ Therefore, the development of new chemoselective oxidation methodologies of sulfides to sulfoxides, avoiding the formation of sulfones, has been increased.^3–5^

One of the most powerful glycosylation methods discovered by Kahne and coworkers employs anomeric glycosyl sulfoxides as glycoside donors.^6^ Since then, several reports describing the study and synthesis of a wide range of glycosides^7–12^ and oligosaccharides^13,14^ using this methodology have been reported. Moreover, glycosyl sulfoxides have demonstrated several biological applications such as the proliferation inhibition of selected tumor cell lines,^15^ oral antithrombotic activity,^16^ and the capability of binding to proteins.^17^ Additionally, in previous works, our research group has synthesized sulfoxide derivates of thiodisaccharides and established the configuration at the sulfur stereocenter by a procedure developed by us employing high resolution 1D and 2D NMR techniques.^18,19^ Furthermore, some of these diastereomeric thiodisaccharides *S*-oxides (with different configurations at the sulfur stereocenter) have demonstrated to inhibit the activity of specific glycosidases, showing different reactivity towards enzymatic hydrolysis according to the *S*-configuration.^18,20^

An important disadvantage of the methodologies described for the oxidation of thiomonosaccharides employing conventional oxidation agents, is the extremely low reaction temperatures required to obtain the respective sulfoxides, without over-oxidation to sulfone.^6,21–23^ Also, it is important to highlight that these conditions are difficult to achieve and control.

Several reagents employed in the sulfide oxidations are toxic, generate by-products difficult to separate from the desired product, and/or contain heavy metals that produce hazardous goods. Furthermore, some of them are very efficient but also very expensive and must be employed stoichiometrically.^24^ In regard to some peroxy acids, certain limitations arise due to the instability of the pure compounds. This is the case of *m*-chloroperbenzoic acid, which is rarely available with a purity higher than 77%.^25^ Furthermore, the use of these compounds should be avoided in production processes since they offer a low atom economy. For all the reasons described above, the development of an energy-saving, catalytic, atom-economical, environmentally friendly, and highly selective oxidation process from thioethers to sulfoxides is required.

Among the different oxidants, molecular oxygen is a flawless reagent since it is “practically unlimited” and “free”, as is light. Photosensitized sulfides oxidation occur according to two main mechanisms (type I and type II).^26,27^ In type I mechanism, an electron transfer between the sulfide and the excited sensitizer takes place, giving rise to sulfide radical cation (RSR′˙^+^) that could follow different pathways:^28^ One of them affords the respective sulfoxide and another one, is the fragmentation to yield a thiyl radical ˙SR′ and alkyl cation R^+^ which evolves to different products. However, the C–S bond cleavage is less common for alkyl sulfides than for aromatic sulfides, and the nature of cleavage products depends on the stabilization of the cation intermediate.^29,30^

On the other hand, in a type II mechanism, an energy transfer process occurs to generate singlet oxygen (^1^O_2_) that is the responsible for the oxidation of the sulfide into a peroxysulfoxide intermediate. This intermediate reacts with another molecule of sulfide to afford the respective sulfoxide.^31^

Several photooxidation methodologies of thioethers to sulfoxides are described in the bibliography.^26,32–37^ Despite the important role of glycosyl sulfoxides in glycosylation reactions and the potential biological applications mentioned above, we were not able to find any study involving the photochemical oxidation of glycosyl sulfides. Therefore, as continuation of our research on the oxidation of thioglycosides, we report here the photosensitized oxidation of thiosaccharides under aerobic conditions. As catalytic amounts of organic dyes, oxygen, and light are employed, instead of the stoichiometric amounts of usually toxic oxidants, generally used for the oxidation of thiosaccharides, this is considered to be a catalytic, efficient, economical, and environmentally friendly method no reported so far for carbohydrates.

## Results and discussion

**Scheme 1:**
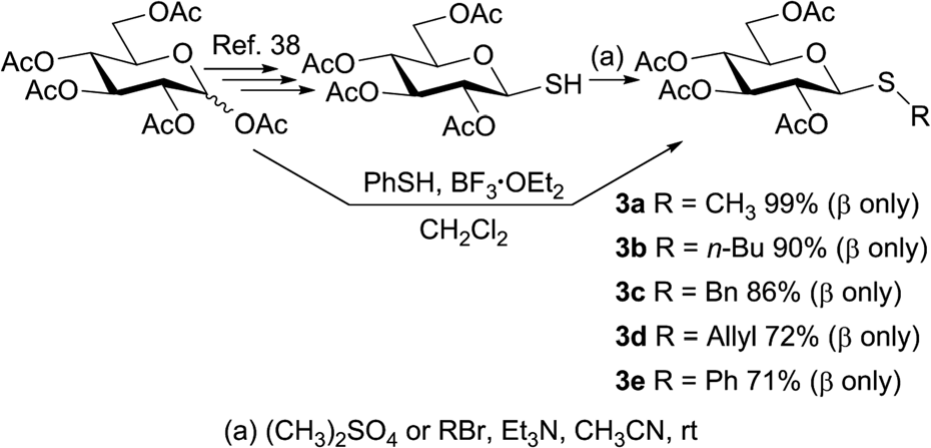
Synthesis of thiomonosaccharides 3a–e.

To study the photosensitized oxidation methodology, the thiosaccharides 3a–e were synthesized and used as substrates. Thus, penta-*O*-acetyl-d-glucopyranose 1 was stereoselectively transformed into the β-thioaldose 2.^38^ Subsequently, 2 was treated with dimethyl sulfate as methylating agent to afford the thioglucoside 3a in 96% isolated yield after simple extractions from the reaction mixture. Other thiomonosaccharides were also synthesized performing the reaction between 2 as glycosyl acceptor and several alkyl/allyl bromides in acetonitrile and in the presence of triethylamine. As shown in Scheme 1, all the obtained products 3a–d maintain the β configuration of the glycosyl acceptor precursor and the isolated yields range from good to excellent (72–99%). The β configuration at the anomeric centers of the alkyl/allyl thioglucose derivates was established according to the large coupling constant value (*J*_1,2_ ≈ 9–10 Hz), determined from the ^1^H NMR spectra.

With the objective of determining the scope of the reaction studied, an aryl thioglycoside was also synthesized employing the procedure described in bibliography.^39^ The phenyl thiosaccharide 3e, having the β-configuration at the anomeric center, was obtained after column chromatography purification in 71% isolated yield (Scheme 1).

With the thiomonosaccharides in hand, we proceeded to perform the photochemical oxidation studies employing diverse photocatalysts under aerobic conditions. To optimize the reaction settings, different variables as photocatalysts, solvents, atmospheres, and time were evaluated using the thiomonosaccharide 3a as a model substrate.

**Chart 1:**
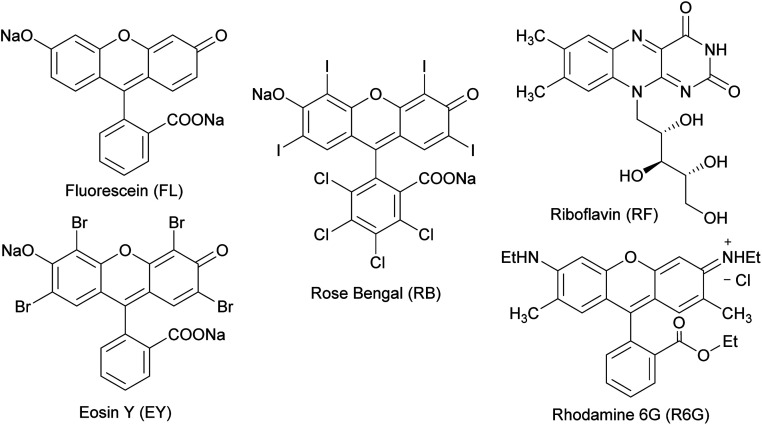
Structures of some organic dyes that are effective photosensitizers capable to generate singlet oxygen under visible light.

Several organic dyes are remarkably effective photosensitizers under visible light, since they possess triplet states of proper energies for sensitization of oxygen.^40,41^ This photosensitization is capable to generate singlet oxygen which is one of the species capable of oxidizing sulfides to sulfoxides. The structures of some of these organic dyes are shown in Chart 1. After each reaction, conversion was determined by ^1^H NMR using the integrals of the 5-H signal of the thiosaccharides (sulfide and sulfoxide). The ratio of the diastereomeric sulfoxides obtained was calculated from specific signals of the respective products.

As starting conditions, the organic dyes were used as photocatalyst (1 mol%) in an oxygen atmosphere, in aprotic or protic polar solvents (acetonitrile and isopropanol, respectively), previously saturated with O_2_. The LED selection to irradiate the organic dyes was performed considering the maximum absorption wavelengths of each sensitizer. When fluorescein was employed, the reaction mixtures were irradiated under blue LED light (467 nm) for 48 h, but the substrate 3a remains unalterable (Table 1, entries 1 and 2).

**Table 1:** Photooxidation of 3a to 4a employing different dyes and solvents

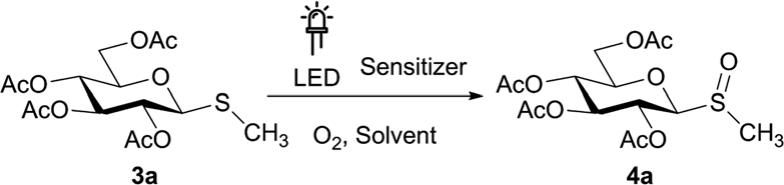				
Entry^a^	Dye (mol%)	*hν* (nm)	Solvent	Yield 4a^b^ (%)
1	FL (1)	467	MeCN	N.R.
2	FL (1)	467	i-PrOH	N.R.
3	FL^−2^ (1)	467	MeCN	N.R.
4	FL^−2^ (1)	467	i-PrOH	N.R.
5	EY (1)	522	MeCN	16
6	EY (1)	522	i-PrOH	15
7	RB (1)	522	MeCN	28
8	RB (1)	522	i-PrOH	37
9	R6G (1)	522	MeCN	84
10	R6G (1)	522	i-PrOH	25

a Reaction conditions: 3a (0.05 M), solvent (2 mL), 45 °C, irradiated with blue LED (467 nm) or green LED (522 nm), oxygen atmosphere, 48 h.

b Determined by ^1^H NMR. N.R. = no reaction.

Subsequently, the reactions were repeated in the presence of NaHCO_3_ to generate the basic form of the photocatalyst, since the production of singlet oxygen by some dye photosensitization was found to be very sensitive to pH changes.^42^ Nevertheless, the thiosaccharide 3a remained also unmodified (Table 1, entries 3 and 4). We have selected this base as is nontoxic and does not affect the substrate (otherwise *O*-deacetylation may take place).

Afterward, the experiments carried out with Eosin Y (EY), Rose Bengal (RB) or rhodamine 6G (R6G) gave the desired diastereomeric sulfoxides 4a in varied yields (Table 1, entries 5–10). The best result was obtained employing rhodamine 6G as photocatalyst, which led to the diastereomeric sulfoxides 4a in 84% yield.

Once selected the best photocatalyst, a solvent screening was evaluated to determine the more efficient medium to perform the photooxidation reaction, as depicted in Table 2.

**Table 2:** Solvent screening for the photooxidation reaction of sulfide 3a to sulfoxide 4a

Entry^a^	Dye (mol%)	Solvent	Yield 4a^b^ (%)
1	R6G (1)	MeCN	84
2	R6G (1)	i-PrOH	25
3	R6G (1)	MeOH	11
4	R6G (1)	Me_2_CO	30
5	R6G (1)	MeCN : MeOH (9 : 1)	81
6	R6G (1)	CH_2_Cl_2_	25
7	R6G (1)	PhMe	N.R.
8	R6G (1)	PEG	N.R.

a Reaction conditions: 3a (0.05 M), solvent (2 mL), 45 °C, irradiated with green LED (522 nm), oxygen atmosphere, 48 h.

b Determined by ^1^H NMR. N.R. = no reaction.

The photooxidation reaction works better in polar solvents as MeCN and in a mixture MeCN : MeOH (9 : 1) (entries 1 and 5, Table 2). Despite the particularly good conversions and high chemoselectivities obtained towards the sulfoxides, we were unsatisfied with the long reaction times required (48 h), without complete consumption of the substrate, probably due to the photocatalyst bleaching under such long periods of irradiation.

As efficient photooxidations of sulfides to sulfoxides, under visible light,^26,34,35^ mediated by riboflavin derivates are reported,^37,43^ we decided to evaluate the photooxidation reactions employing tetra-*O*-acetyl riboflavin (RFTA) as photocatalyst. First, riboflavin (RF) was peracetylated employing a protocol described in the bibliography.^44^ Then, the photooxidation reactions were performed under various conditions, as shown in Table 3. When MeCN, MeOH, EtOH or H_2_O were employed as solvents, the degree of conversion was low (entries 1–4). Surprisingly, in MeCN : H_2_O (85 : 15) an excellent yield (99%) and chemoselectivity were achieved in considerably lower reaction times (entry 5). This result was in agreement with the obtained by Neveselý *et al.* for oxidation reactions performed in such solvent mixture to avoid catalyst aggregation.^43^ Replacement of MeCN : H_2_O (85 : 15) with EtOH : H_2_O (95 : 5) as solvent mixture led to a lower isolated yield (57%) for the same reaction time (entry 6). Since no bleaching of the RFTA was observed, the reaction was repeated extending the irradiation time to 6 h, leading to complete conversion with excellent chemoselectivity (entry 7). Subsequently, a few control experiments were carried out at longer reaction times. In the absence of an oxygen atmosphere, photocatalyst or an irradiation source, the sulfoxide 4a was not produced and the substrate 3a remains unchanged (entries 8–10). In contrast, when the photooxidation reaction was carried out in MeCN : H_2_O (85 : 15) and air atmosphere excellent conversion and selectivity was also obtained in 6 h (entry 11). For comparison, the air atmosphere was also tested in EtOH : H_2_O (95 : 5) leading to incomplete conversion even after 24 h of irradiation (64%, entry 12).

**Table 3:** Solvent screening and reaction conditions employing RFTA as sensitizer

Entry^a^	Dye (mol%)	Solvent	Additive	*hν* (nm)	Atm	Time (h)	Yield 4a^b^ (%)
1	RFTA (2)	MeCN	—	467	O_2_	24	24
2	RFTA (2)	MeOH	—	467	O_2_	24	14
3	RFTA (2)	EtOH	—	467	O_2_	24	25
4	RFTA (2)	H_2_O	—	467	O_2_	24	13
5	RFTA (2)	MeCN : H_2_O (85 : 15)	—	467	O_2_	2	99
6	RFTA (2)	EtOH : H_2_O (95 : 5)	—	467	O_2_	2	57
7	RFTA (2)	EtOH : H_2_O (95 : 5)	—	467	O_2_	6	99
8	RFTA (2)	MeCN : H_2_O (85 : 15)	—	467	N_2_	24	N.R.
9	RFTA (2)	MeCN : H_2_O (85 : 15)	—	Dark	O_2_	24	N.R.
10	—	MeCN : H_2_O (85 : 15)	—	467	O_2_	24	N.R.
11	RFTA (2)	MeCN : H_2_O (85 : 15)	—	467	Air	6	>99
12	RFTA (2)	EtOH : H_2_O (95 : 5)	—	467	Air	24	64
13	RFTA (2)	MeCN : H_2_O (85 : 15)	NaN_3_	467	O_2_	24	N.R.
14	RFTA (2)	MeCN : H_2_O (85 : 15)	DABCO	467	O_2_	24	N.R.

a Reaction conditions: 3a (0.05 M), solvent (2 mL), 45 °C.

b Determined by ^1^H NMR. N.R. = no reaction.

To evidence the formation of singlet oxygen in the media some additional control reactions were performed. As sodium azide and 1,4-diazabicyclo[2.2.2]octane (DABCO) are specific and efficient quenchers of singlet oxygen,^45–48^ the reactions were conducted in the presence of these additives. As expected, formation of the respective sulfoxides 4a was completely inhibited (entries 13 and 14).

Finally, to determine the scope of the photooxidation reaction, different thiosaccharides 3a–f were oxidized under the optimized experimental conditions (Table 4).

**Table 4:** Scope of the photooxidation reaction employing different per-*O*-acetylated thiosaccharides under the optimized experimental conditions

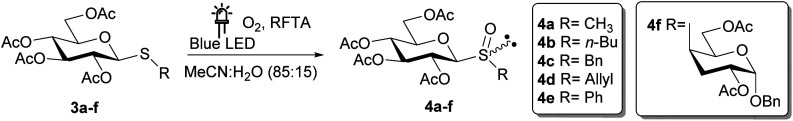					
Entry^a^	Sulfide	Time (h)	Conversion (%)	Isolated yield 4^b^ (%)	Diastereomeric ratio^c^ S_*S*_/S_*R*_
1	3a	2	100	99	1.6/1.0
2	3b	2	100	93	2.0/1.0
3	3c	6	94	57	1.5/1.0
4	3d	6	84	60	1.5/1.0
5	3e	6	0	0	N.R.
6	3f	6	0	0	N.R.
7	3f	24	0	0	N.R.

a Reaction conditions: 3 (0.05 M), solvent (2 mL), 45 °C, oxygen atmosphere (balloon).

b isolated yield.

c determined by ^1^H NMR. N.R. = no reaction.

As summarized in Table 4, an excellent total isolated yield (99%) was obtained for the oxidation of 3a to sulfoxide 4a (entry 1). This was in fact a diastereomeric mixture, which could be chromatographically separated affording the S_*S*_ and S_*R*_ sulfoxides in 61% and 37% isolated yields, respectively. The photooxidation reaction of 3b was also highly efficient, affording the diastereomeric mixture of sulfoxides 4b after 2 h (isolated yield 93%). On the other hand, the conversion of substrates 3c and 3d was incomplete after 6 h of irradiation, and the isolated yields fell to 57% and 60% respectively (entries 3 and 4).

These results were not surprising since it was described that C–S bond cleavage can occur when benzyl and allyl sulfides undergo photooxidation under singlet oxygen conditions, leading to lower sulfoxide yields.^49–51^ However, it is important to highlight that the photooxidation reaction of the allyl sulfide 3d was completely chemoselective, and the sulfoxide 4d was obtained without oxidation of the vinyl group.

Unfortunately, no evidence of an oxidation reaction was obtained for the sulfide 3e, as this substrate was recovered unchanged after 6 h under irradiation (entry 5). Probably the excited state of the photocatalyst is quenched prior to the formation of singlet oxygen by some interaction with 3e.

**Scheme 2:**
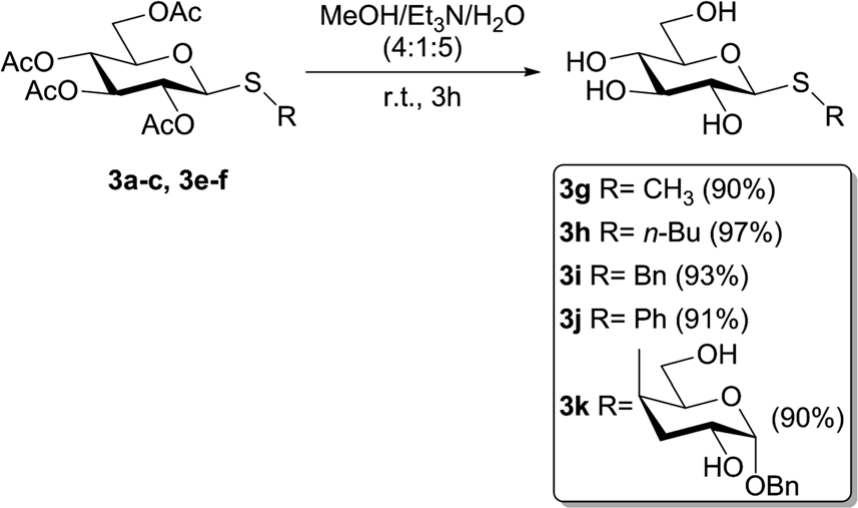
De-*O*-acetylation of thiosaccharides 3a–c, 3e–f.

To evaluate if the studied photochemical reaction could be applied to oxidize thiodisaccharides, the compound 3f was synthesized using a protocol described by our research group.^52^ The fact that no chemical changes of the thiodisaccharide were observed during the photooxidation, even after 24 h of irradiation, indicated that no reaction took place (Table 4, entries 6 and 7). This result may be explained considering the mechanism proposed in the bibliography.^26,27^ The peroxysulfoxide intermediate generated from the thiodisaccharide 3f needs to react with another molecule of this compound to afford the desired sulfoxide. The large steric hindrance produced by the sugar groups could prevent the peroxysulfoxide intermediate to evolve to the sulfoxide and consequently returns to the substrate. Displeased with these results, the removal of the acetyl protecting group of 3a, 3b, 3c, 3e, and 3f was performed (Scheme 2), since the oxidation potential of some carbohydrates can be modified by changing the protecting groups attached to these molecules.^53^ The de-*O*-acetylation reactions were carried out under mild conditions employing a mixture of MeOH/Et_3_N/H_2_O (4 : 1 : 5)^54–56^ to afford the free thiosaccharides 3g–k in very good to excellent isolated yields (90–97%).

**Scheme 3:**
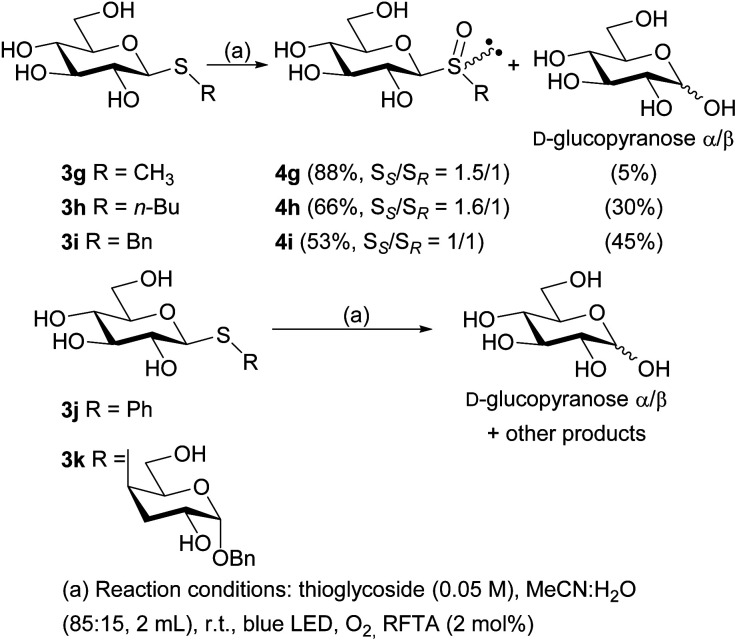
Photooxidation reactions of free thiosaccharides 3g–k.

With the free thioglycosides in hand, the photooxidation reactions were conducted under the optimized conditions. Similar to the photooxidation of its analogue 3a, the sulfide 3g gave excellent results, as the diastereomeric mixture of sulfoxides 4g was obtained in 88% isolated yield (ratio S_*S*_/S_*R*_ = 1.5/1) under irradiation for only 30 min. A mixture of α and β anomers of d-glucopyranose was also isolated as a minor product (5%). The diastereomeric mixture of free sulfoxides 4h and 4i were also obtained in lower reaction times than their peracetylated analogues, although in lower yields (66 and 53% yield, respectively). In addition, a major amount of d-glucopyranose was also obtained (Scheme 3).

Unfortunately, the photooxidation reaction of sulfide 3j showed incomplete consumption of the starting material after 8 h and the corresponding diastereomeric sulfoxides were not obtained. Instead, a complex mixture was formed, probably as result of radical pathways in the oxidative medium. Most of these products remained unidentified, although from the reaction crude, a mixture of both anomers of d-glucopyranose was isolated as main product (yield 35%, ratio *α*/*β* = 1 : 1.6) (Scheme 3). Similarly, when the free thiodisaccharide 3k was subjected to the photooxidation a complex mixture was obtained. With the purpose of determining the structure of such products, the reaction crude was peracetylated employing pyridine and acetic anhydride (1 : 1), and subsequently subjected to column chromatography purification. The ^1^H NMR spectrum of one of the fractions displayed characteristic signals evidencing the presence of peracetylated glucose (ratio *α*/*β* = 1 : 1.2). These facts demonstrate that a competitive fragmentation reaction took place in these cases probably *via* a radical cation intermediate generated by the oxidation of the thiosaccharides 3g–k through an electron transfer reaction (type I mechanism), which is the main reaction for 3j and 3k.

The stereocontrol in the oxidation reactions was provided by the asymmetric induction of the glucosyl residue, which favors the formation of the sulfoxides with S_*S*_ configuration in almost all cases.^57,58^ These results agree with previous reports describing that thioglycosides with α-configuration lead predominantly to sulfoxides with the S_*R*_ absolute configuration at the sulfur atom, while their β-anomers lead to diastereomeric mixtures of S_*S*_ (major) and S_*R*_ sulfoxides.^21,22,59^

The configuration at the sulfur stereocenter of each sulfoxide obtained was established employing the methodology developed by our research group.^18,19^ In this protocol, the anisotropic effect of the S=O group on the chemical shift of specific protons in the ^1^H NMR spectra must be considered. To perform this type of analysis, it is necessary to determine the rotamers, formed by rotation of the thioglycosidic linkage, present in the conformational equilibrium. As β-glucopyranosides populate almost exclusively the ^4^C_1_ conformation, each rotamer is characterized by specific NOE interactions observed in the corresponding 2D-NOESY spectra.

**Fig. 1:**
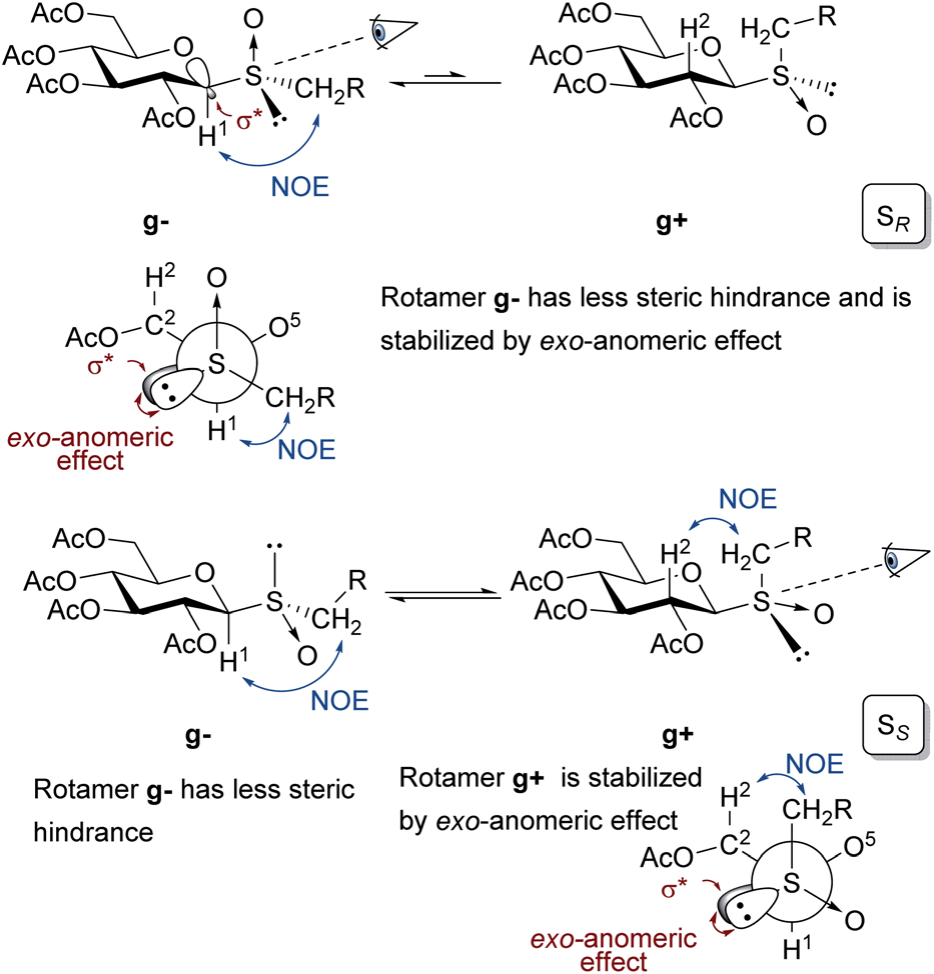
Conformations displayed by rotation of the anomeric linkage of β-glucopyranosyl sulfoxides with S_*R*_ or S_*S*_ configuration depicted for the ^4^C_1_ chair and in Newman projections.

As depicted in Fig. 1, in the sulfoxides with the S_*R*_ configuration, the rotamer *gauche*g− is favored because disposes the residue CH_2_R in a position with less steric hindrance and it is stabilized by the *exo*-anomeric effect.

This was justified by interresidue NOE contacts between the sugar-H^1^ and the methylene protons of the CH_2_R observed in the 2D-NOESY spectra. The lack of the NOE contact between sugar-H^2^–CH_2_R suggested the almost exclusive presence of the g− conformer. On the other hand, in the sulfoxides with the S_*S*_ configuration, both rotamers g− and g+ are stabilized by different factors. The first one (g−), arranges the residue CH_2_R in a position with less steric hindrance, while the second one (g+) presents stabilization by the *exo*-anomeric effect. This fact explains the coexistence of these two rotamers in equilibrium and was demonstrated by observing the NOE contacts between sugar-H^1^–CH_2_R protons for the g− rotamer, and the spatial interaction between sugar-H^2^–CH_2_R for the g+ rotamer. These results are in agreement with those reported by Sanhueza *et al.* in their study on the stereochemical properties of structurally related glucosyl sulfoxides.^60^

Once determined the preferential rotamers, the differences in the chemical shift for signals of specific protons in the ^1^H NMR spectra were analyzed for each sulfoxide. The shielding and deshielding effects were explained considering the position of the S=O group and the sulfur lone pair relative to the protons H^1^ and H^2^ of the thiomonosaccharides 4a–d, 4g–i. The sulfoxides with the S_*R*_ configuration in the most populated rotamer g− arranges the lone pair of electrons of the sulfur in the direction of the H^1^, generating a shielding effect, while H^2^ should be deshielded as result of the 1,3-diaxial interaction between the C2–H^2^ bond and the S=O group. To perform this kind of analysis in the sulfoxides with the S_*S*_ configuration both rotamers g− and g+ must be considered. In the rotamer g− the H^1^ is located in the S=O anisotropic deshielding region, although this effect could be partially countered by the protection effect generated by the proximity to the sulfur lone pair in the g+ rotamer. The shielding effect observed for the H^2^ protons could be explained by the 1,3-diaxial interaction with the sulfur lone pair in the g− rotamer. Furthermore, an additional shielding contribution should be generated by the proximity of the sulfur lone pair to H^2^ in the g+ rotamer, as evidenced in the Newman projection. As consequence of all these effects, the signal of proton H^1^ in S_*R*_ sulfoxides are expected to be shifted upfield compared to that of H^1^ in S_*S*_ sulfoxides, while the protons H^2^ are deshielded (higher *δ* value) in the sulfoxides S_*R*_ sulfoxides compared to S_*S*_ sulfoxides (Table 5).

**Table 5:** Chemical shift values obtained for H^1^ and H^2^ of the glucosyl sulfoxides 4a–d in CDCl_3_ and 4g–i in D_2_O

Sulfoxide	*δ*H^1^ (ppm)	*δ*H^2^ (ppm)
4a(S_*R*_)	4.15	5.44
4a(S_*S*_)	4.38	5.06
4b(S_*R*_)	4.16	5.44
4b(S_*S*_)	4.34	5.21
4c(S_*R*_)	3.83	5.44
4c(S_*S*_)	4.05	5.24
4d(S_*R*_)	4.16	5.43
4d(S_*S*_)	4.33	5.34
4g(S_*R*_)	4.25	3.74
4g(S_*S*_)	4.63	3.66
4h(S_*R*_)	4.28	3.74
4h(S_*S*_)	4.62	3.71
4i(S_*R*_)	4.07	3.76
4i(S_*S*_)	4.58	3.64

Some glycosyl sulfoxides, such as 4a*S* and 4a*R*, have been previously synthesized.^57^ The fact that their physical and spectral data agree with those of the same products obtained in this work, and which configurations at the sulfur stereocenter has been assigned using the procedure described above, serve as validation of this methodology developed by us.

Only the peracetylated glucosyl sulfoxides containing R = CH_3_, could be separated by column chromatography, and all the other sulfoxide derivates were obtained as their diastereomeric mixtures. Nevertheless, applications of glycosyl sulfoxides as glycosyl donors allows the use of the diastereomeric mixture, since the stereochemical outcome in glycosylation reactions is independent of the sulfoxide stereochemistry (S_*R*_ or S_*S*_).^61^

## Experimental

### Materials and methods

All photocatalysts were acquired commercially, except for tetra-*O*-acetyl riboflavin which was synthesized by a known procedure^44^ from commercially available riboflavin. Column chromatography was carried out with silica gel 60 (230–400 mesh). Analytical thin-layer chromatography (TLC) was carried out on silica gel 60 F254 aluminium-backed plates (layer thickness 0.2 mm). The spots were visualized by charring with a solution of (NH_4_)_6_Mo_7_O_24_·4H_2_O 25 g L^−1^, (NH_4_)_4_Ce(SO_4_)_4_·2H_2_O 10 g L^−1^ and 10% H_2_SO_4_ in H_2_O. Nuclear magnetic resonance (NMR) spectra were recorded at 400 MHz (^1^H) or 100 MHz (^13^C). Chemical shifts were calibrated to tetramethylsilane or to a residual solvent peak (CHCl_3_: ^1^H: *δ* = 7.26 ppm, ^13^C: *δ* = 77.2 ppm or H_2_O: *δ* = 4.79 ppm, respectively). Assignments of ^1^H and ^13^C NMR spectra were assisted by 2D ^1^H-COSY and 2D ^1^H–^13^C HSQC and HMBC experiments. For the assignment of the NMR signals, the H and C atoms of the aglycone residue have been labeled as depicted for each individual compound in the ESI.†

The coupling constants values are reported in Hz and resonance multiplicities abbreviated as follows: s = singlet, d = doublet, t = triplet, q = quartet, p = pentet, sx = sextet, m = multiplet, br = broad. High-resolution mass spectra (HRMS) were obtained using the electrospray ionization (ESI) technique and Q-TOF detection.

### Synthetic procedures

#### General procedure for synthesis of thiomonosaccharides 3a–d

The β-thioaldose 2^38^ (200 mg, 0.5 mmol) was dissolved in acetonitrile (2 mL), triethylamine (380 μL, 2.5 mmol, 5 eq.) and the electrophile (0.55 mmol, 1.1 eq.) were added. The reaction mixture was stirred at 30 °C for 2 hours, until TLC showed complete consumption of the starting materials. The reaction mixture was concentrated under reduced pressure and diluted with EtOAc (50 mL), washed with HCl 0.1 M (20 mL × 3), dried over anhydrous Na_2_SO_4_, filtered, and concentrated under reduced pressure.

##### Methyl 2,3,4,6-tetra-*O*-acetyl-1-thio-β-d-glucopyranoside (3a)

Following the general procedure, the synthesis of 3a was performed adding dimethyl sulfate (52 μL, 0.55 mmol) as the electrophile to the basic solution of 2. Upon reaction completion, the reaction mixture was diluted with acetonitrile, extracted with hexane (20 mL × 3) to remove excess of dimethyl sulfate, and then treated as was mentioned above. The thiosaccharide 3a^62–64^ (205.6 mg, 99%) was obtained as a white solid, m.p. at 86.8 °C (dec.). *R*_f_ = 0.60, hexane/EtOAc (1 : 1). [*α*]^24^_D_ = −11.9 (*c* = 0.8, CHCl_3_). ^1^H NMR (400 MHz, CDCl_3_): *δ* = 5.20 (t, *J*_2,3_ = *J*_3_,_4_ = 9.4 Hz, 1H, 3-H), 5.05 (t, *J*_3,4_ = *J*_4,5_ = 9.7 Hz, 1H, 4-H), 5.04 (t, *J*_1,2_ = *J*_2,3_ = 9.6 Hz, 1H, 2-H), 4.37 (d, *J*_1,2_ = 10.0 Hz, 1H, 1-H), 4.22 (dd, *J*_6a,6b_ = 12.4, *J*_5-6a_ = 4.8 Hz, 1H, 6a-H), 4.12 (dd, *J*_6a,6b_ = 12.4, *J*_5-6b_ = 1.9 Hz, 1H, 6b-H), 3.71 (ddd, *J*_4,5_ = 9.9, *J*_5,6a_ = 4.5, *J*_5,6b_ = 2.1 Hz, 1H, 5-H), 2.14 (s, 3H, CH_3_S), 2.05, 2.04, 2.00, 1.98 (4s, 12H, COCH_3_) ppm. ^13^C NMR (100 MHz, CDCl_3_): *δ* = 170.8, 170.3, 169.6 (×2) (*C*OCH_3_), 83.0 (C-1), 76.1 (C-5), 74.0 (C-3), 69.2 (C-2), 68.4 (C-4), 62.2 (C-6), 20.9, 20.8, 20.7 (×2) (CO*C*H_3_), 11.4 (CH_3_S) ppm. HRMS (ESI): calcd for C_15_H_22_NaO_9_S 401.0877 [M + Na]^+^; found 401.0862.

##### Butyl 2,3,4,6-tetra-*O*-acetyl-1-thio-β-d-glucopyranoside (3b)

Following the general procedure, the synthesis of 3b^63,65,66^ was performed adding *n*-butyl bromide (59 μL, 0.55 mmol) to the basic solution of 2. The thiosaccharide 3b (224 mg, 97%) was obtained as a white solid, m.p. 69.1–70.0 °C. *R*_f_ = 0.75, hexane/EtOAc (1 : 1). [*α*]^24^_D_ = −26.9 (*c* = 0.9, CHCl_3_). ^1^H NMR (400 MHz, CDCl_3_): *δ* = 5.21 (t, *J*_2,3_ = *J*_3,4_ = 9.4 Hz, 1H, 3-H), 5.07 (t, *J*_3,4_ = *J*_4,5_ = 9.9 Hz, 1H, 4-H), 5.02 (dd, *J*_1,2_ = 10.1, *J*_2,3_ = 9.4 Hz, 1H, 2-H), 4.47 (d, *J*_1,2_ = 10.0 Hz, 1H, 1-H), 4.23 (dd, *J*_6a,6b_ = 12.4, *J*_5,6a_ = 5.0 Hz, 1H, 6a-H), 4.13 (dd, *J*_6a,6b_ = 12.3, *J*_5,6b_ = 2.4 Hz, 1H, 6b-H), 3.70 (ddd, *J*_4,5_ = 10.0, *J*_5,6a_ = 4.9, *J*_5,6b_ = 2.4 Hz, 1H, 5-H), 2.73–2.60 (m, 2H, a-H), 2.07, 2.05, 2.02, 2.00 (4s, 12H, COCH_3_), 1.60–1.53 (m, 2H, b-H), 1.39 (sx, *J*_b,c_ = *J*_c,d_ = 7.4 Hz, 2H, c-H), 0.90 (t, *J*_c,d_ = 7.3 Hz, 3H, d-H) ppm. ^13^C NMR (100 MHz, CDCl_3_): *δ* = 170.8, 170.3, 169.6, 169.5 (*C*OCH_3_), 83.8 (C-1), 76.0 (C-5), 74.1 (C-3), 70.0 (C-2), 68.5 (C-4), 62.3 (C-6), 31.8 (C-b), 29.8 (C-a), 22.0 (C-c), 20.9 (×2), 20.7 (×2) (CO*C*H_3_), 13.7 (C-d) ppm. HRMS (ESI): calcd for C_18_H_28_NaO_9_S 443.1346 [M + Na]^+^; found 443.1357.

##### Benzyl 2,3,4,6-tetra-*O*-acetyl-1-thio-β-d-glucopyranoside (3c)

Following the general procedure, the synthesis of 3c was performed adding benzyl bromide (65 μL, 0.55 mmol) to the basic solution of 2. The thiosaccharide 3c^63,66,67^ (215 mg, 86%) was obtained as a white solid, m.p. at 93.8 °C (dec.). *R*_f_ = 0.65, hexane/EtOAc (1 : 1). [*α*]^24^_D_ = −86.5 (*c* = 1.0, CHCl_3_). ^1^H NMR (400 MHz, CDCl_3_): *δ* = 7.34–7.29 (m, 5H, *Ph*CH_2_O), 5.16–5.04 (m, 3H, 3-H, 2-H, 4-H), 4.29 (d, *J*_1,2_ = 9.7 Hz, 1H, 1-H), 4.23 (dd, *J*_6a,6b_ = 12.4, *J*_5,6a_ = 5.1 Hz, 1H, 6a-H), 4.13 (dd, *J*_6a,6b_ = 12.3, *J*_5,6b_ = 2.2 Hz, 1H, 6b-H), 3.94 (d, *J*_gem_ = 12.9 Hz, 1H, a-H), 3.83 (d, *J*_gem_ = 12.9 Hz, 1H, a-H), 3.59 (ddd, *J*_4,5_ = 9.6, *J*_5,6a_ = 5.0, *J*_5,6b_ = 2.3 Hz, 1H, 5-H), 2.11, 2.01 (×2), 1.99 (4s, 12H, COCH_3_) ppm. ^13^C NMR (100 MHz, CDCl_3_): *δ* = 170.7, 170.3, 169.5 (×2) (*C*OCH_3_), 137.0, 129.2, 128.8, 127.6 (C-aromatics), 82.2 (C-1), 76.0 (C-5), 74.0 (C-3), 70.0 (C-2), 68.6 (C-4), 62.4 (C-6), 34.0 (C-a), 20.9, 20.8, 20.7 (×2) (CO*C*H_3_) ppm. HRMS (ESI): calcd for C_21_H_26_NaO_9_S 477.1190 [M + Na]^+^; found 477.1222.

##### Allyl 2,3,4,6-tetra-*O*-acetyl-1-thio-β-d-glucopyranoside (3d)

Following the general procedure, the synthesis of 3d was carried out adding allyl bromide (47 μL, 0.55 mmol) to the basic solution of 2. The thiosaccharide 3d^68^ (160 mg, 72%) was obtained as a colorless syrup. *R*_f_ = 0.67, hexane/EtOAc (1 : 1). [*α*]^25^_D_ = −15.9 (*c* = 0.9, CHCl_3_). ^1^H NMR (400 MHz, CDCl_3_): *δ* = 5.81–5.71 (m, 1H, b-H), 5.18 (t, *J*_2,3_ = *J*_3,4_ = 9.3 Hz, 1H, 3-H), 5.13–5.08 (m, 2H, c-H, c′-H), 5.02 (t, *J*_3,4_ = *J*_4,5_ = 9.7 Hz, 1H, 4-H), 5.01 (dd, *J*_1,2_ = 9.9, *J*_2,3_ = 9.4 Hz, 1H, 2-H), 4.45 (d, *J*_1,2_ = 10.1 Hz, 1H, 1-H), 4.19 (dd, *J*_6a,6b_ = 12.3, *J*_5,6a_ = 5.2 Hz, 1H, 6a-H), 4.09 (dd, *J*_6a,6b_ = 12.3, *J*_5,6b_ = 2.3 Hz, 1H, 6b-H), 3.62 (ddd, *J*_4,5_ = 10.0, *J*_5,6a_ = 5.1, *J*_5,6b_ = 2.3 Hz, 1H, 5-H), 3.35 (dd, *J*_gem_ = 13.5, *J*_a,b_ = 8.4 Hz, 1H, a-H), 3.19 (dd, *J*_gem_ = 13.5, *J*_a,b_ = 6.1 Hz, 1H, a-H), 2.04, 2.00, 1.98, 1.96 (4s, 12H, COCH_3_) ppm. ^13^C NMR (100 MHz, CDCl_3_): *δ* = 170.6, 170.2, 169.4 (×2) (*C*OCH_3_), 133.5 (C-b), 118.0 (C-c), 82.0 (C-1), 75.8 (C-5), 74.0 (C-3), 70.0 (C-2), 68.6 (C-4), 62.3 (C-6), 32.9 (C-a), 20.7 (×2), 20.6 (×2) (CO*C*H_3_) ppm. HRMS (ESI): calcd for C_17_H_24_NaO_9_S 427.1033 [M + Na]^+^; found 427.1014.

##### Phenyl 2,3,4,6-tetra-*O*-acetyl-1-thio-β-d-glucopyranoside (3e)

This compound was synthesized employing a procedure previously described.^39^ To a solution of penta-*O*-acetyl glucopyranose 1 (975 mg, 2.5 mmol) in anhydrous CH_2_Cl_2_ (5.0 mL) was added thiophenol (0.3 mL, 3 mmol), followed by the dropwise addition of BF_3_·OEt_2_ (1.6 mL, 12.5 mmol) under argon atmosphere. The reaction mixture was stirred 4 h until complete consumption of the starting materials. The mixture was diluted to 50 mL in CH_2_Cl_2_ and it was extracted with water (2 × 50 mL), sodium bicarbonate (1 × 50 mL), and brine (1 × 50 mL). The organic phase was dried over anhydrous Na_2_SO_4_, filtered, and concentrated under reduced pressure. Column chromatography of the residue using pentane/EtOAc (4 : 1 → 1 : 1) afforded 3e^63,67,69,70^ (782 mg, 71%) as a white solid, m.p. 116.3–117.9 °C. *R*_f_ = 0.47, pentane/EtOAc (2 : 1). ^1^H NMR (400 MHz, CDCl_3_): *δ* = 7.51–7.48 (m, 2H, aromatic), 7.32–7.30 (m, 3H, aromatic), 5.22 (t, *J*_2,3_ = *J*_3,4_ = 9.3 Hz, 1H, 3-H), 5.04 (t, *J*_3,4_ = *J*_4,5_ = 9.8 Hz, 1H, 4-H), 4.97 (dd, *J*_1,2_ = 10.0, *J*_2,3_ = 9.3 Hz, 1H, 2-H), 4.71 (d, *J*_1,2_ = 10.1 Hz, 1H, 1-H), 4.22 (dd, *J*_6a,6b_ = 12.3, *J*_5-6a_ = 5.0 Hz, 1H, 6a-H), 4.18 (dd, *J*_6a,6b_ = 12.3, *J*_5,6b_ = 2.6 Hz, 1H, 6b-H), 3.72 (ddd, *J*_4,5_ = 10.0, *J*_5,6a_ = 5.0, *J*_5,6b_ = 2.7 Hz, 1H, 5-H), 2.08 (×2), 2.01, 1.99 (4s, 12H, COCH_3_) ppm. ^13^C NMR (100 MHz, CDCl_3_): *δ* = 170.7, 170.3, 169.5, 169.4 (*C*OCH_3_), 133.3, 131.8, 129.1, 128.6 (aromatic), 85.9 (C-1), 76.0 (C-5), 74.1 (C-3), 70.1 (C-2), 68.4 (C-4), 62.3 (C-6), 20.9, 20.8, 20.7 (×2) (CO*C*H_3_) ppm. HRMS (ESI): calcd for C_20_H_24_NaO_9_S 463.1033 [M + Na]^+^; found 463.0989.

##### Benzyl 3-deoxy-2,6-di-*O*-acetyl-4-*S*-(2,3,4,6-tetra-*O*-acetyl-β-d-glucopyranosyl)-4-thio-α-d-*xylo*-hexopyranoside (3f)

Thiodisaccharide 3f was obtained following a protocol described by our research group^52^ as a white solid, m.p. at 110 °C (dec.). *R*_f_ = 0.53 (pentane/EtOAc, 1 : 1). [*α*]^25^_D_ = +28.5 (*c* = 1.2, CHCl_3_) ^1^H NMR (400 MHz, CDCl_3_): *δ* = 7.36–7.29 (m, 5H, aromatic), 5.21 (m, 2H, 2-H, 3′-H), 5.08 (t, *J*_3′,4′_ = *J*_4′,5′_ = 9.7 Hz, 1H, 4′-H), 5.01 (t, *J*_1′,2′_ = *J*_2′,3′_ = 9.6 Hz, 1H, 2′-H), 5.01 (d, *J*_1,2_ = 3.2 Hz, 1H, 1-H), 4.74 (d, *J*_gem_ = 12.0 Hz, 1H, PhC*H*_2_O), 4.61 (d, *J*_1′,2′_ = 10.0 Hz, 1H, 1′-H), 4.52 (d, *J*_gem_ = 12.0 Hz, 1H, PhC*H*_2_O), 4.36 (m, 1H, 5-H), 4.21 (dd, *J*_6′a,6′b_ = 12.5, *J*_5′,6′a_ = 4.6 Hz, 1H, 6′a-H), 4.16 (m, 2H, 6a-H, 6b-H), 4.13 (dd, *J*_6′a,6′b_ = 12.5, *J*_5′,6′b_ = 2.3 Hz, 1H, 6′b-H), 3.67 (ddd, *J*_4′,5′_ = 9.9, *J*_5′,6′a_ = 4.4, *J*_5′,6′b_ = 2.4 Hz, 1H, 5′-H), 3.39 (br d, *J* = 2.1 Hz, 1H, 4-H), 2.34 (td, *J*_2,3a_ = *J*_3a,3b_ = 12.6, *J*_3a,4_ = 3.6 Hz, 1H, 3a-H), 2.14 (m, 1H, 3b-H), 2.07, 2.06 (×2), 2.03, 2.01, 1.99 (COCH_3_) ppm. ^13^C NMR (100 MHz, CDCl_3_): *δ* = 170.7, 170.6, 170.2 (×2), 169.7, 169.4 (COCH_3_), 137.3, 128.6, 128.1, 128.0 (C-Ph), 94.9 (C-1), 82.8 (C-1′), 76.1 (C-5′), 73.9 (C-3′), 70.0 (C-2′), 69.2 (PhCH_2_O), 68.3 (×2, C-4′, C-5), 66.9 (C-2), 65.4 (C-6), 62.0 (C-6′), 42.9 (C-4), 30.9 (C-3), 21.1, 20.9, 20.8, 20.7 (×3) (COCH_3_). HRMS (ESI): calcd for C_31_H_40_NaO_15_S 707.1980 [M + Na]^+^; found 707.1959.

#### Synthesis of unprotected thiosaccharides 3g–k

##### Methyl 1-thio-β-d-glucopyranoside (3g)

The thiosaccharide 3a (37.8 mg, 0.1 mmol) was dissolved in MeOH/Et_3_N/H_2_O (4 : 1 : 5, 0.55 mL) and the reaction mixture was stirred at 30 °C for 2 h. When TLC showed complete consumption of the starting material, the mixture was concentrated under reduced pressure. Column chromatography using CH_2_Cl_2_/MeOH (4 : 1 → 2 : 1) afforded 3g^71–73^ (18.9 mg, 90%) as a colorless syrup. *R*_f_ = 0.40, CH_2_Cl_2_/MeOH (4 : 1). [*α*]^23^_D_ = −17.2 (*c* = 1.1, H_2_O) ^1^H NMR (400 MHz, D_2_O): *δ* = 4.49 (d, *J*_1,2_ = 9.8 Hz, 1H, 1-H), 3.96 (dd, *J*_6a,6b_ = 12.4, *J*_5,6a_ = 2.1 Hz, 1H, 6a-H), 3.77 (dd, *J*_6a,6b_ = 12.5, *J*_5,6b_ = 5.6 Hz, 1H, 6b-H), 3.55 (t, *J*_2,3_ = *J*_3,4_ = 8.8 Hz, 1H, 3-H), 3.54–3.50 (m, 1H, 5-H), 3.46 (dd, *J*_3,4_ = 8.9, *J*_4,5_ = 9.6 Hz, 1H, 4-H), 3.41 (dd, *J*_1,2_ = 9.7, *J*_2,3_ = 8.8 Hz, 1H, 2-H), 2.27 (s, 3H, CH_3_S) ppm. ^13^C NMR (100 MHz, D_2_O): *δ* = 85.6 (C-1), 79.9 (C-5), 77.2 (C-3), 71.7 (C-2), 69.6 (C-4), 60.9 (C-6), 11.4 (CH_3_S) ppm. HRMS (ESI): calcd for C_7_H_14_NaO_5_S 233.0454 [M + Na]^+^; found 233.0447.

##### Butyl 1-thio-β-d-glucopyranoside (3h)

The thiosaccharide 3b (538.0 mg, 1.28 mmol) was dissolved in MeOH/Et_3_N/H_2_O (4 : 1 : 5, 7.2 mL) and the reaction mixture was stirred at 30 °C for 3 h. When TLC showed complete consumption of the starting material, the mixture was concentrated under reduced pressure. Column chromatography using CH_2_Cl_2_/MeOH (8 : 1) afforded 3h (312 mg, 97%) as a colorless syrup. *R*_f_ = 0.78, CH_2_Cl_2_/MeOH (4 : 1). [*α*]^23^_D_ = −40.1 (*c* = 1.2, MeOH) ^1^H NMR (400 MHz, D_2_O): *δ* = 4.55 (d, *J*_1,2_ = 9.9 Hz, 1H, 1-H), 3.93 (dd, *J*_6a,6b_ = 12.4, *J*_5,6a_ = 1.6 Hz, 1H, 6a-H), 3.73 (dd, *J*_6a,6b_ = 12.4, *J*_5,6b_ = 5.5 Hz, 1H, 6b-H), 3.51 (t, *J*_2,3_ = *J*_3,4_ = 8.6 Hz, 1H, 3-H), 3.48 (ddd, *J*_4,5_ = 9.4, *J*_5,6b_ = 5.4, *J*_5,6a_ = 1.6 Hz, 1H, 5-H), 3.43 (t, *J*_3,4_ = *J*_4,5_ = 9.3 Hz, 1H, 4-H), 3.34 (t, *J*_1,2_ = *J*_2,3_ = 9.3 Hz, 1H, 2-H), 2.85–2.72 (m, 2H, a-H), 1.65 (p, *J*_a,b_ = *J*_b,c_ = 7.4 Hz, 2H, b-H), 1.43 (sx, *J*_b,c_ = *J*_c,d_ = 7.4 Hz, 2H, c-H), 0.92 (t, *J*_c,d_ = 7.4 Hz, 3H, d-H) ppm. ^13^C NMR (100 MHz, D_2_O): *δ* = 85.4 (C-1), 79.9 (C-5), 77.3 (C-3), 72.4 (C-2), 69.6 (C-4), 61.0 (C-6), 31.5 (C-b), 29.7 (C-a), 21.3 (C-c), 12.9 (C-d) ppm. HRMS (ESI): calcd for C_10_H_20_NaO_5_S 275.0924 [M + Na]^+^; found 275.0929.

##### Benzyl 1-thio-β-d-glucopyranoside (3i)

The thiosaccharide 3c (260.0 mg, 0.57 mmol) was dissolved in MeOH/Et_3_N/H_2_O (4 : 1 : 5, 3.2 mL) and the reaction mixture was stirred at 30 °C for 3 h. When TLC showed complete consumption of the starting material, the mixture was concentrated under reduced pressure. Column chromatography using CH_2_Cl_2_/MeOH (8 : 1) afforded 3i (152.3 mg, 93%) as a colorless syrup. *R*_f_ = 0.82, CH_2_Cl_2_/MeOH (4 : 1). [*α*]^23^_D_ = −129.4 (*c* = 1.0, MeOH) ^1^H NMR (400 MHz, D_2_O): *δ* = 7.45–7.35 (m, 5H, aromatic), 4.34 (d, *J*_1,2_ = 9.4 Hz, 1H, 1-H), 4.05 (d, *J*_gem_ = 13.2 Hz, 1H, a-H), 3.96 (d, *J*_gem_ = 13.3 Hz, 1H, a-H), 3.88 (dd, *J*_6a,6b_ = 12.5, *J*_5,6a_ = 2.1 Hz, 1H, 6a-H), 3.71 (dd, *J*_6a,6b_ = 12.5, *J*_5,6b_ = 5.6 Hz, 1H, 6b-H), 3.44–3.34 (m, 4H, 4-H, 3-H, 2-H, 5-H) ppm. ^13^C NMR (100 MHz, D_2_O): *δ* = 138.0, 129.1, 128.8, 127.4 (aromatic), 84.0 (C-1), 79.8(C-5), 77.3(C-3), 72.2 (C-2), 69.5(C-4), 60.9 (C-6), 33.6 (C-a) ppm. HRMS (ESI): calcd for C_13_H_18_NaO_5_S 309.0767 [M + Na]^+^; found 309.0770.

##### Phenyl 1-thio-β-d-glucopyranoside (3j)

The thiosaccharide 3e (44.1 mg, 0.1 mmol) was dissolved in MeOH/Et_3_N/H_2_O (4 : 1 : 5, 0.55 mL) and the reaction mixture was stirred at 30 °C for 3 h. After TLC showed complete consumption of the starting material, the mixture was concentrated under reduced pressure and the residue was purified by column chromatography using CH_2_Cl_2_/MeOH (4 : 1 → 2 : 1), affording 3j^74,75^ (24.5 mg, 91%) as a white solid, m.p. at 128.5 °C (dec.). *R*_f_ = 0.66, CH_2_Cl_2_/MeOH (4 : 1). [*α*]^24^_D_ = +49.2 (*c* = 1.0, EtOH) ^1^H NMR (400 MHz, D_2_O): *δ* = 7.65–7.63 (m, 3H, aromatic), 7.50–7.44 (m, 3H, aromatic), 4.85 (d, *J*_1,2_ = 9.9 Hz, 1H, 1-H), 3.95 (dd, *J*_6a,6b_ = 12.5, *J*_5-6a_ = 2.2 Hz, 1H, 6a-H), 3.77 (dd, *J*_6a,6b_ = 12.5, *J*_5-6b_ = 5.6 Hz, 1H, 6b-H), 3.58 (t, *J*_2,3_ = *J*_3,4_ = 8.9 Hz, 1H, 3-H), 3.56–3.51 (m, 1H, 5-H), 3.47 (dd, *J*_4,5_ = 9.7, *J*_3,4_ = 9.0 Hz, 1H, 4-H), 3.41 (dd, *J*_1,2_ = 9.8, *J*_2,3_ = 9.0 Hz, 1H, 2-H) ppm. ^13^C NMR (100 MHz, D_2_O): *δ* = 132.0, 131.7, 129.4, 128.1 (aromatic), 87.3 (C-1), 79.9 (C-5), 77.3 (C-3), 71.8 (C-2), 69.4 (C-4), 60.9 (C-6) ppm. HRMS (ESI): calcd for C_12_H_16_NaO_5_S 295.0611 [M + Na]^+^; found 295.0619.

##### Benzyl 3-deoxy-4-*S*-(β-d-glucopyranosyl)-4-thio-α-d-*xylo*-hexopyranoside (3k)

The thiodisaccharide 3f (68.4 mg, 0.1 mmol) was dissolved in MeOH/Et_3_N/H_2_O (4 : 1 : 5, 0.84 mL) and the reaction mixture was stirred at 30 °C for 3 h. When TLC showed complete consumption of the starting material into a more polar product (*R*_f_ = 0.74, BuOH/EtOH/H_2_O (10 : 4 : 4)), the mixture was concentrated under reduced pressure. Subsequently, purification of the residue by column chromatography using CH_2_Cl_2_/MeOH (4 : 1 → 2 : 1) afforded 3k (40.2 mg, 93%) as a colorless syrup. ^1^H NMR (400 MHz, D_2_O): *δ* = 7.55–7.44 (m, 5H, aromatic), 5.02 (d, *J*_1,2_ = 3.9 Hz, 1H, 1-H), 4.84 (d, *J*_gem_ = 11.7 Hz, 1H, PhC*H*_2_O), 4.71 (d, *J*_gem_ = 11.8 Hz, 1H, PhC*H*_2_O), 4.64 (d, *J*_1′,2′_ = 9.8 Hz, 1H, 1′-H), 4.26–4.16 (m, 2H, 5-H, 2-H), 3.95 (dd, *J*_6′a,6′b_ = 12.3 Hz, *J*_5′,6′a_ = 2.0 Hz, 1H, 6′a-H), 3.77 (dd, *J*_6a,6b_ = 11.8, *J*_5,6a_ = 5.0 Hz, 1H, 6a-H), 3.75 (dd, *J*_6′a,6′b_ = 12.4, *J*_5′,6′b_ = 5.4 Hz, 1H, 6′b-H), 3.63 (dd, *J*_6a,6b_ = 11.8, *J*_5,6b_ = 7.4 Hz, 1H, 6b-H), 3.58–3.43 (m, 4H, 4-H, 3′-H, 4′-H, 5′-H), 3.36 (dd, *J*_1′,2′_ = 9.7, *J*_2′,3′_ = 8.9 Hz, 1H, 2′-H), 2.29–2.16 (m, 2H, 3a-H, 3b-H) ppm. ^13^C NMR (100 MHz, D_2_O): *δ* = 137.3, 128.8, 128.7, 128.4 (aromatic), 97.5 (C-1), 84.9 (C-1′), 80.0 (C-4′), 77.3 (C-3′), 72.6 (C-2′), 70.6 (C-5), 69.8 (Ph*C*H_2_O), 69.6 (C-5′), 64.2 (C-2), 62.5 (C-6), 61.0 (C-6′), 42.9 (C-4), 32.7 (C-3) ppm. HRMS (ESI): calcd for C_19_H_28_NaO_9_S 455.1346 [M + Na]^+^; found 455.1369.

#### General procedure for the photooxidation of glucopyranosyl sulfides

Sulfide 3a–k (0.1 mmol) and the photocatalyst (1–10 mol%, 0.001–0.01 mmol) were dissolved in the solvent mixture in a glass vial, equipped with a rubber septum and a magnetic stirrer. The reaction mixture was saturated with oxygen by bubbling with a balloon for 5 minutes and irradiated with a 3 W LED with continuous stirring. The average temperature value in the reaction vial was determined to be 42 °C. The balloon was left to ensure constant supply of oxygen into the reaction vial. The reaction course was monitored by TLC (hexane/EtOAc, 1 : 1). For sulfoxides 4a–d, the reaction mixtures were purified by column chromatography with hexane/EtOAc (8 : 1 → 1 : 1), while sulfoxides 4g–i were purified using MeCN/H_2_O (9 : 1 → 4 : 1) or CH_2_Cl_2_/MeOH (8 : 1 → 4 : 1).

##### Methyl 2,3,4,6-tetra-*O*-acetyl-1-thio-β-d-glucopyranoside (*S*)-*S*-oxide (4a*S*)

The major product of the oxidation of thiosaccharide 3a was sulfoxide 4a*S*^57^ (24 mg, 61%) obtained as a colorless syrup. *R*_f_ = 0.25, EtOAc. [*α*]^23^_D_ = −12.2 (*c* = 0.9, CHCl_3_). ^1^H NMR (400 MHz, CDCl_3_): *δ* = 5.31 (t, *J*_2,3_ = *J*_3,4_ = 9.3 Hz, 1H, 3-H), 5.11 (dd, *J*_4,5_ = 9.9, *J*_3,4_ = 9.6 Hz, 1H, 4-H), 5.07 (dd, *J*_1,2_ = 10.0, *J*_2,3_ = 9.4 Hz, 1H, 2-H), 4.38 (d, *J*_1,2_ = 10.2 Hz, 1H, 1-H), 4.32 (dd, *J*_6a,6b_ = 12.6, *J*_5,6a_ = 4.5 Hz, 1H, 6a-H), 4.21 (dd, *J*_6a,6b_ = 12.6, *J*_5,6b_ = 2.2 Hz, 1H, 6b-H), 3.84 (ddd, *J*_4,5_ = 10.1, *J*_5,6a_ = 4.5, *J*_5,6b_ = 2.2 Hz, 1H, 5-H), 2.68 (s, 3H, CH_3_SO), 2.09, 2.07, 2.04, 2.02 (4s, 12H, COCH_3_) ppm. ^13^C NMR (100 MHz, CDCl_3_): *δ* = 170.6, 170.1, 169.9, 169.5 (*C*OCH_3_), 90.9 (C-1), 77.0 (C-5), 73.3 (C-3), 68.4 (C-2), 67.8 (C-4), 61.5 (C-6), 33.0 (CH_3_SO), 20.8, 20.7 (×3) (CO*C*H_3_) ppm. HRMS (ESI): calcd for C_15_H_22_NaO_10_S 417.0826 [M + Na]^+^; found 417.0756.

##### Methyl 2,3,4,6-tetra-*O*-acetyl-1-thio-β-d-glucopyranoside (*R*)-*S*-oxide (4a*R*)

The minor product of the oxidation of thiosaccharide 3a was sulfoxide 4a*R*^57^ (14.6 mg, 36%) obtained as a colorless syrup. *R*_f_ = 0.20, EtOAc. [*α*]^23^_D_ = −64.7 (*c* = 0.8, CHCl_3_). ^1^H NMR (400 MHz, CDCl_3_): *δ* = 5.44 (t, *J*_1,2_ = *J*_2,3_ = 9.6 Hz, 1H, 2-H), 5.36 (t, *J*_2,3_ = *J*_3,4_ = 9.3 Hz, 1H, 3-H), 5.15 (dd, *J*_4,5_ = 9.9, *J*_3,4_ = 9.4 Hz, 1H, 4-H), 4.27 (dd, *J*_6a,6b_ = 12.7, *J*_5,6a_ = 5.1 Hz, 1H, 6a-H), 4.23 (dd, *J*_6a,6b_ = 12.7, *J*_5,6b_ = 3.0 Hz, 1H, 6b-H), 4.15 (d, *J*_1,2_ = 9.8 Hz, 1H, 1-H), 3.82 (ddd, *J*_4,5_ = 10.1, *J*_5,6a_ = 4.9, *J*_5,6b_ = 2.9 Hz, 1H, 5-H), 2.69 (s, 3H, CH_3_SO), 2.08, 2.06, 2.05, 2.03 (4s, 12H, COCH_3_) ppm. ^13^C NMR (100 MHz, CDCl_3_): *δ* = 170.7, 170.6, 169.3, 169.1 (*C*OCH_3_), 87.6 (C-1), 77.0 (C-5), 73.8 (C-3), 67.9 (C-4), 67.0 (C-2), 62.0 (C-6), 33.2 (CH_3_SO), 20.8 (×2), 20.7 (×2) (CO*C*H_3_) ppm. HRMS (ESI): calcd for C_15_H_22_NaO_10_S 417.0826 [M + Na]^+^; found 417.0798.

##### Butyl 2,3,4,6-tetra-*O*-acetyl-1-thio-β-d-glucopyranoside (*R*,*S*)-*S*-oxide (4b*R*,*S*)

The oxidation reaction of thiosaccharide 3b gave the diastereomeric mixture of sulfoxides 4b*R*,*S* that could not be separated by column chromatography. The mixture (40.5 mg, 93%, ratio *S*/*R*, 2 : 1) was obtained as a white solid and showed a single spot by TLC analysis. *R*_f_ = 0.17, hexane/EtOAc (1 : 1). ^1^H NMR (400 MHz, CDCl_3_) data for *S* isomer: *δ* = 5.29 (t, *J*_2,3_ = *J*_3,4_ = 9.2 Hz, 1H, 3-H_S_), 5.21 (t, *J*_1,2_ = *J*_2,3_ = 9.6 Hz, 1H, 2-H_S_), 5.09 (dd, *J*_4,5_ = 9.7, *J*_3,4_ = 9.4 Hz, 1H, 4-H_S_ overlapping with 4-H_R_ of 4b*R*), 4.34 (d, *J* = 9.9 Hz, 1H, 1-H_S_), 4.27 (dd, *J*_6a,6b_ = 12.6, *J*_5,6a_ = 4.6 Hz, 1H, 6a-H_S_), 4.17 (dd, *J*_6a,6b_ = 12.6, *J*_5,6b_ = 2.3 Hz, 1H, 6b-H_S_), 3.82–3.78 (m, 1H, 5-H_S_,overlapping with 5-H_R_ of 4b*R*), 2.96–2.89 (m, 1H, a-H_S_), 2.83–2.76 (m, 1H, a-H_S_), 2.07–2.01 (4s, 12H, COCH_3_ overlapping with COCH_3_ of 4b*R*), 1.79–1.73 (m, 2H, b-H_S_ overlapping with b-H_R_ of 4b*R*), 1.57–1.43 (m, 2H, c-H_S_ overlapping with c-H_R_ of 4b*R*), 0.96 (t, *J*_c,d_ = 7.3 Hz, 3H, d-H_S_ overlapping with d-H_R_ of 4b*R*) ppm. ^13^C NMR (100 MHz, CDCl_3_) data for *S* isomer: *δ* = 170.6–168.9 (*C*OCH_3_ overlapping with *C*OCH_3_ of 4b*R*), 90.4 (C-1_S_), 77.0 (C-5_S_), 73.3 (C-3_S_), 68.5 (C-2_S_), 67.8 (C-4_S_), 61.6 (C-6_S_), 47.2 (C-a_S_), 24.2 (C-b_S_), 22.1 (C-c_S_), 20.8–20.7 (CO*C*H_3_ overlapping with CO*C*H_3_ of 4b*R*), 13.8 (C-d_S_ overlapping with C-d_R_ of 4b*R*) ppm. ^1^H NMR (400 MHz, CDCl_3_) data for *R* isomer: *δ* = 5.44 (dd, *J*_1,2_ = 9.8, *J*_2,3_ = 9.4 Hz, 1H, 2-H_R_), 5.35 (t, *J*_2,3_ = *J*_3,4_ = 9.3 Hz, 1H, 3-H_R_), 5.12 (dd, *J*_4,5_ = 10.0, *J*_3,4_ = 9.5 Hz, 1H, 4-H_R_ overlapping with 4-H_S_ of 4b*S*), 4.27–4.21 (m, 2H, 6a-H_R_, 6b-H_R_), 4.16 (d, *J*_1,2_ = 9.9 Hz, 1H, 1-H_R_), 3.82–3.78 (m, 1H, 5-H_R_,overlapping with 5-H_S_ of 4b*S*), 3.16–3.09 (m, 1H, a-H_R_), 2.74–2.67 (m, 1H, a-H_R_), 2.07–2.01 (4s, 12H, COCH_3_ overlapping with COCH_3_ of 4b*S*), 1.79–1.73 (m, 2H, b-H_R_ overlapping with b-H_S_ of 4b*S*), 1.57–1.43 (m, 2H, c-H_R_ overlapping with c-H_S_ of 4b*S*), 0.96 (t, *J*_c,d_ = 7.3 Hz, 3H, d-H_R_ overlapping with d-H_S_ of 4b*S*) ppm. ^13^C NMR (100 MHz, CDCl_3_) data for *R* isomer: *δ* = 170.6–168.9 (*C*OCH_3_ overlapping with *C*OCH_3_ of 4b*S*), 87.0 (C-1_R_), 77.0 (C-5_R_), 73.9 (C-3_R_), 68.0 (C-4_R_), 67.0 (C-2_R_), 62.1 (C-6_R_), 47.1 (C-a_R_), 24.9 (C-b_R_), 22.3 (C-c_R_), 20.8–20.7 (CO*C*H_3_ overlapping with CO*C*H_3_ of 4b*S*), 13.8 (C-d_R_ overlapping with C-d_S_ of 4b*S*) ppm. HRMS (ESI): calcd for C_18_H_28_NaO_10_S 459.1295 [M + Na]^+^; found 459.1271.

##### Benzyl 2,3,4,6-tetra-*O*-acetyl-1-thio-β-d-glucopyranoside (*R*,*S*)-*S*-oxide (4c*R*,*S*)

The oxidation reaction of thiosaccharide 3c gave the diastereomeric mixture of sulfoxides 4c*R*,*S*^76^ that could not be separated by column chromatography. The mixture (26.8 mg, 57%, ratio *S*/*R*, 1.5 : 1) was obtained as a white solid and showed a single spot in TLC analysis. *R*_f_ = 0.12, hexane/EtOAc (1 : 1). ^1^H NMR (400 MHz, CDCl_3_) data for *S* isomer: *δ* = 7.41–7.32 (m, 5H, aromatic_S_ overlapping with aromatic_R_ of 4c*R*), 5.28 (t, *J*_1,2_ = *J*_2,3_ = 9.3 Hz, 1H, 2-H_S_), 5.24 (t, *J*_2,3_ = *J*_3,4_ = 9.1 Hz, 1H, 3-H_S_), 5.14–5.07 (m, 1H, 4-H_S_ overlapping with 4-H_R_ of 4c*R*), 4.33–4.26 (m, 1H, 6a-H_S_ overlapping with 6b-H_R_, a-H_R_), 4.24 (dd, *J*_6a,6b_ = 12.6, *J*_5,6b_ = 2.4 Hz, 1H, 6b-H_S_), 4.16 (d, *J*_gem_ = 13.0 Hz, 1H, a-H_S_), 4.16 (d, *J*_1,2_ = 9.8 Hz, 1H, 1-H_S_), 4.07 (d, *J*_gem_ = 13.0 Hz, 1H, a-H_S_), 3.77 (ddd, *J*_4,5_ = 9.9, *J*_5,6a_ = 4.6, *J*_5,6b_ = 2.3 Hz, 1H, 5-H_S_), 2.16–2.00 (4s, 12H, COCH_3_ overlapping with COCH_3_ of 4c*R*) ppm. ^13^C NMR (100 MHz, CDCl_3_) data for *S* isomer: *δ* = 170.6–168.7 (*C*OCH_3_ overlapping with *C*OCH_3_ of 4c*R*), 130.7–128.7 (aromatic_S_ overlapping with aromatic_R_ of 4c*R*), 88.9 (C-1_S_), 76.9 (C-5_S_), 73.2 (C-3_S_), 68.6 (C-2_S_), 67.9 (C-4_S_), 61.7 (C-6_S_), 53.8 (C-a_S_), 20.9–20.6 (CO*C*H_3_ overlapping with CO*C*H_3_ of 4c*R*) ppm. ^1^H NMR (400 MHz, CDCl_3_) data for *R* isomer: *δ* = 7.41–7.31 (m, 5H, aromatic_R_ overlapping with aromatic_S_ of 4c*S*), 5.44 (dd, *J*_1,2_ = 10.1, *J*_2,3_ = 9.3 Hz, 1H, 2-H_R_), 5.22 (t, *J*_2,3_ = *J*_3,4_ = 9.3 Hz, 1H, 3-H_R_), 5.14–5.07 (m, 1H, 4-H_R_ overlapping with 4-H_S_ of 4c*S*), 4.41 (d, *J*_gem_ = 12.3 Hz, 1H, a-H_R_), 4.35 (dd, *J*_6a,6b_ = 12.5, *J*_5,6a_ = 2.5 Hz, 1H, 6a-H_R_), 4.33–4.26 (m, 2H, 6b-H_R_, a-H_R_ overlapping with 6a-H_S_ of 4c*S*), 3.83 (d, *J*_1,2_ = 10.2 Hz, 1H, 1-H_R_), 3.73 (ddd, *J*_4,5_ = 10.0, *J*_5,6a_ = 6.1, *J*_5,6b_ = 2.3 Hz, 1H, 5-H_R_), 2.16–2.00 (4s, 12H, COCH_3_ overlapping with COCH_3_ of 4c*S*) ppm. ^13^C NMR (100 MHz, CDCl_3_) data for *R* isomer: *δ* = 170.6–168.7 (*C*OCH_3_ overlapping with *C*OCH_3_ of 4c*S*), 130.7–128.7 (aromatic_R_ overlapping with aromatic_S_ of 4c*S*), 84.6 (C-1_R_), 77.1 (C-5_R_), 73.9 (C-3_R_), 68.1 (C-4_R_), 66.5 (C-2_R_), 62.6 (C-6_R_), 53.5 (C-a_R_), 20.9–20.6 (CO*C*H_3_ overlapping with CO*C*H_3_ of 4c*S*) ppm. HRMS (ESI): calcd for C_21_H_26_NaO_10_S 493.1139 [M + Na]^+^; found 493.1115.

##### Allyl 2,3,4,6-tetra-*O*-acetyl-1-thio-β-d-glucopyranoside (*R*,*S*)-*S*-oxide (4d*R*,*S*)

The oxidation reaction of thiosaccharide 3d gave the diastereomeric mixture of sulfoxides 4d*R*,*S* that could not be separated by column chromatography. The mixture (25.3 mg, 60%, ratio *S*/*R*, 1.5 : 1) was obtained as a white solid and showed a single spot by TLC. *R*_f_ = 0.10, hexane/EtOAc (1 : 1). ^1^H NMR (400 MHz, CDCl_3_) data for *S* isomer: *δ* = 6.00–5.89 (m, 1H, b-H_S_), 5.51–5.41 (m, 2H, c-H_S_, c′-H_S_ overlapping with c-H_R_, c′-H_R_, 2-H_R_ of 4d*R*), 5.34 (t, *J*_1,2_ = *J*_2,3_ = 9.2 Hz, 1H, 2-H_S_), 5.29 (t, *J*_2,3_ = *J*_3,4_ = 9.1 Hz, 1H, 3-H_S_), 5.08 (t, *J*_3,4_ = *J*_4,5_ = 9.2 Hz, 1H, 4-H_S_), 4.33 (d, *J*_1,2_ = 9.4 Hz, 1H, 1-H_S_), 4.28–4.22 (m, 1H, 6a-H_S_ overlapping with 6a-H_R_, 6b-H_R_, 1-H_R_ of 4d*R*), 4.19 (dd, *J*_6a,6b_ = 12.6, *J*_5,6b_ = 2.1 Hz, 1H, 6b-H_S_), 3.80–3.76 (m, 1H, 5-H_S_ overlapping with 5-H_R_ of 4d*R*), 3.66 (dd, *J*_gem_ = 13.3, *J*_a,b_ = 7.0 Hz, 1H, a-H_S_), 3.57 (dd, *J*_gem_ = 13.1, *J*_a,b_ = 8.0 Hz, 1H, a-H_S_), 2.09–2.02 (4s, 12H, COCH_3_ overlapping with COCH_3_ of 4d*R*) ppm. ^13^C NMR (100 MHz, CDCl_3_) data for *S* isomer: *δ* = 170.5–168.8 (*C*OCH_3_ overlapping with *C*OCH_3_ of 4d*R*), 125.5 (C-b_S_), 124.3 (C-c_S_), 89.0 (C-1_S_), 76.9 (C-5_S_), 73.3 (C-3_S_), 68.6 (C-2_S_), 67.8 (C-4_S_), 61.7 (C-6_S_), 52.0 (C-a_S_), 20.8–20.6 (CO*C*H_3_ overlapping with CO*C*H_3_ of 4d*R*) ppm. ^1^H NMR (400 MHz, CDCl_3_) data for *R* isomer: *δ* = 5.84–5.73 (m, 1H, b-H_R_), 5.51–5.41 (m, 3H, c-H_R_, c′-H_R_, 2-H_R_ overlapping with c-H_S_, c′-H_S_ of 4d*S*), 5.35 (t, *J*_2,3_ = *J*_3,4_ = 9.3 Hz, 1H, 3-H_R_), 5.11 (t, *J*_3,4_ = *J*_4,5_ = 9.7 Hz, 1H, 4-H_R_), 4.28–4.22 (m, 3H, 6a-H_R_, 6b-H_R_, 1-H_R_ overlapping with 6a-H_S_ of 4d*S*), 3.83 (dd, *J*_gem_ = 12.7, *J*_a,b_ = 9.0 Hz, 1H, a-H_R_), 3.80–3.76 (m, 1H, 5-H_R_ overlapping with 5-H_S_ of 4d*S*), 3.75 (dd, *J*_gem_ = 12.6, *J*_a,b_ = 7.0 Hz, 1H, a-H_R_), 2.09–2.02 (4s, 12H, COCH_3_ overlapping with COCH_3_ of 4d*S*) ppm. ^13^C NMR (100 MHz, CDCl_3_) data for *R* isomer: *δ* = 170.5–168.8 (*C*OCH_3_ overlapping with *C*OCH_3_ of 4d*S*), 125.6 (C-b_R_), 124.3 (C-c_R_), 85.5 (C-1_R_), 77.0 (C-5_R_), 73.3 (C-3_R_), 68.1 (C-4_R_), 66.7 (C-2_R_), 62.3 (C-6_R_), 51.8 (C-a_R_), 20.8–20.6 (CO*C*H_3_ overlapping with CO*C*H_3_ of 4d*S*) ppm. HRMS (ESI): calcd for C_17_H_24_NaO_10_S 443.0982 [M + Na]^+^; found 443.0980.

##### Methyl 1-thio-β-d-glucopyranoside (*R*,*S*)–*S*-oxide (4g*R*,*S*)

The oxidation reaction of the free thiosaccharide 3g gave the diastereomeric mixture of sulfoxides 4g*R*,*S* that could not be separated by column chromatography. The mixture (19.9 mg, 88%, ratio *S*/*R*, 1.5 : 1) was obtained as a white solid and showed a single spot by TLC analysis. *R*_f_ = 0.38, MeCN/H_2_O (4 : 1). ^1^H NMR (400 MHz, D_2_O) data for *S* isomer: *δ* = 4.63 (d, *J*_1,2_ = 9.7 Hz, 1H, 1-H_S_), 4.00 (dd, *J*_6a,6b_ = 12.6, *J*_5,6a_ = 2.1 Hz, 1H, 6a-H_S_), 3.83 (dd, *J*_6a,6b_ = 12.6, *J*_5,6b_ = 5.9 Hz, 1H, 6b-H_S_), 3.71–3.61 (m, 3H, 3-H_S_, 2-H_S_, 5-H_S_ overlapping with 3-H_R_, 5-H_R_ of 4g*R*), 3.50 (t, *J*_3,4_ = *J*_4,5_ = 9.3 Hz, 1H, 4-H_S_), 2.85 (s, 3H, CH_3_SO_S_) ppm. ^13^C NMR (100 MHz, D_2_O) data for *S* isomer: *δ* = 91.0 (C-1_S_), 80.8 (C-5_S_), 77.2 (C-3_S_), 69.4 (C-2_S_), 69.0 (C-4_S_), 60.9 (C-6_S_), 30.7 (CH_3_SO_S_) ppm. ^1^H NMR (400 MHz, D_2_O) data for *R* isomer: *δ* = 4.25 (d, *J*_1,2_ = 9.6 Hz, 1H, 1-H_R_), 4.03 (dd, *J*_6a,6b_ = 12.7, *J*_5,6a_ = 2.2 Hz, 1H, 6a-H_R_), 3.88 (dd, *J*_6a,6b_ = 12.6, *J*_5,6b_ = 4.9 Hz, 1H, 6b-H_R_), 3.74 (dd, *J*_1,2_ = 9.5, *J*_2,3_ = 9.2 Hz, 1H, 2-H_R_), 3.71–3.61 (m, 2H, 3-H_R_, 5-H_R_ overlapping with 3-H_S_, 2-H_S_, 5-H_S_ of 4g*S*), 3.55 (t, *J*_3,4_ = *J*_4,5_ = 9.2 Hz, 1H, 4-H_R_), 2.86 (s, 3H, CH_3_SO_R_) ppm. ^13^C NMR (100 MHz, D_2_O) data for *R* isomer: *δ* = 89.3 (C-1_R_), 80.3 (C-5_R_), 77.0 (C-3_R_), 68.9 (C-4_R_), 68.0 (C-2_R_), 60.6 (C-6_R_), 31.7 (CH_3_SO_R_) ppm. HRMS (ESI): calcd for C_7_H_14_NaO_6_S 249.0403 [M + Na]^+^; found 249.0395.

##### Butyl 1-thio-β-d-glucopyranoside (*R*,*S*)-*S*-oxide (4h*R*,*S*)

The oxidation reaction of the free thiosaccharide 3h afforded after purification by column chromatography the diastereomeric mixture of sulfoxides 4h*R*,*S*. The mixture (19.1 mg, 66%, ratio *S*/*R*, 1.6 : 1) was obtained as a colorless syrup and showed a single spot by TLC analysis. *R*_f_ = 0.58, CH_2_Cl_2_/MeOH (4 : 1). ^1^H NMR (400 MHz, D_2_O) data for *S* isomer: *δ* = 4.62 (d, *J*_1,2_ = 9.7 Hz, 1H, 1-H_S_), 3.96 (dd, *J*_6a,6b_ = 12.6, *J*_5,6a_ = 2.1 Hz, 1H, 6a-H_S_), 3.79 (dd, *J*_6a,6b_ = 12.8, *J*_5,6b_ = 6.1 Hz, 1H, 6b-H_S_), 3.71 (t, *J*_1,2_ = *J*_2,3_ = 9.3 Hz, 1H, 2-H_S_), 3.64 (t, *J*_2,3_ = *J*_3,4_ = 9.0 Hz, 1H, 3-H_S_), 3.59 (ddd, *J*_4,5_ = 9.7, *J*_5,6b_ = 5.9, *J*_5,6a_ = 2.1 Hz, 1H, 5-H_s_), 3.46 (br t, *J*_3,4_ = *J*_4,5_ = 9.4 Hz, 1H, 3-H_s_), 3.30–3.23 (m, 1H, a-H_s_ overlapping with a-H_R_ of 4h*R*), 3.02–2.93 (m, 1H, a-H_s_ overlapping with a-H_R_ of 4h*R*), 1.85–1.66 (m, 2H, b-H_s_ overlapping with b-H_R_ of 4h*R*) 1.60–1.45 (m, 2H, c-H_s_ overlapping with c-H_R_ of 4h*R*), 0.97(t, *J*_c,d_ = 7.3 Hz, 3H, d-H_s_) ppm. ^13^C NMR (100 MHz, D_2_O) data for *S* isomer: *δ* = 90.8 (C-1_S_), 80.8 (C-5_S_), 77.2 (C-3_S_), 69.1 (C-2_S_), 68.9 (C-4_S_), 60.8 (C-6_S_), 44.8 (C-a_S_), 24.1 (C-b_s_ overlapping with C-b_R_), 21.2 (C-c_S_), 12.9 (C-d_s_) ppm. ^1^H NMR (400 MHz, D_2_O) data for *R* isomer: *δ* = 4.28 (d, *J*_1,2_ = 9.8 Hz, 1H, 1-H_R_), 3.97 (dd, *J*_6a,6b_ = 12.7, *J*_5,6a_ = 2.2 Hz, 1H, 6a-H_R_), 3.83 (dd, *J*_6a,6b_ = 13.0, *J*_5,6b_ = 5.1 Hz, 1H, 6b-H_R_), 3.74 (t, *J*_1,2_ = *J*_2,3_ = 9.7 Hz, 1H, 2-H_R_), 3.66 (t, *J*_2,3_ = *J*_3,4_ = 8.7 Hz, 1H, 3-H_R_), 3.64–3.59 (m, 1H, 5-H_R_), 3.52 (br t, *J*_3,4_ = *J*_4,5_ = 9.4 Hz, 1H, 3-H_R_), 3.30–3.23 (m, 1H, a-H_R_ overlapping with a-H_S_ of 4h*S*), 3.02–2.93 (m, 1H, a-H_R_ overlapping with a-H_S_ of 4h*S*), 1.85–1.66 (m, 2H, b-H_R_ overlapping with b-H_S_ of 4h*S*) 1.60–1.45 (m, 2H, c-H_R_ overlapping with c-H_S_ of 4h*S*), 0.97(t, *J*_c,d_ = 7.4 Hz, 3H, d-H_R_) ppm. ^13^C NMR (100 MHz, D_2_O) data for *R* isomer: *δ* = 88.2 (C-1_R_), 80.3 (C-5_R_), 77.0 (C-3_R_), 68.7 (C-4_R_), 67.8 (C-2_R_), 60.5 (C-6_R_), 45.7 (C-a_R_), 24.1 (C-b_R_ overlapping with C-b_S_), 21.3 (C-c_R_), 12.9 (C-d_R_) ppm HRMS (ESI): calcd for C_10_H_20_NaO_6_S 291.0873 [M + Na]^+^; found 291.0879.

##### Benzyl 1-thio-β-d-glucopyranoside (*R*,*S*)-*S*-oxide (4i*R*,*S*)

The oxidation reaction of the free thiosaccharide 3i afforded the diastereomeric mixture of sulfoxides **4i*R*,*S*** after purification by column chromatography. The mixture (18.4 mg, 53%, ratio *S*/*R*, 1 : 1) was obtained as a white solid and showed a single spot by TLC analysis. *R*_f_ = 0.56, CH_2_Cl_2_/MeOH (4 : 1). ^1^H NMR (400 MHz, D_2_O) data for *S* isomer: *δ* = 7.53–7.46 (m, 5H, aromatic_S_ overlapping with aromatic_R_ of 4i*R*), 4.58 (d, *J*_1,2_ = 9.7 Hz, 1H, 1-H_S_), 4.48 (d, *J*_gem_ = 13.2 Hz, 1H, a-H_S_), 4.41 (d, *J*_gem_ = 13.1 Hz, 1H, a-H_S_), 4.00 (dd, *J*_6a,6b_ = 12.6, *J*_5,6a_ = 2.0 Hz, 1H, 6a-H_S_), 3.84 (dd, *J*_6a,6b_ = 12.6, *J*_5,6b_ = 5.8 Hz, 1H, 6b-H_S_), 3.66–3.45 (m, 4H, 2-H_S_, 3-H_S_, 5-H_S,_ 4-H_S_ overlapping with 3-H_R_, 5-H_R,_ 4-H_R_ of 4i*R*) ppm. ^13^C NMR (100 MHz, D_2_O) data for *S* isomer: *δ* = 130.6–128.9 (aromatic_S_ overlapping with aromatic_R_ of 4i*R*), 90.8 (C-1_S_), 80.9 (C-5_S_), 77.2 (C-3_S_), 69.4 (C-2_S_), 68.9 (C-4_S_), 60.8 (C-6_S_), 51.7 (C-a_S_) ppm. ^1^H NMR (400 MHz, D_2_O) data for *R* isomer: *δ* = 7.53–7.46 (m, 5H, aromatic_R_ overlapping with aromatic_s_ of 4i*S*), 4.53 (d, *J*_gem_ = 12.6 Hz, 1H, a-H_R_), 4.42 (d, *J*_gem_ = 12.5 Hz, 1H, a-H_R_), 4.07 (d, *J*_1,2_ = 10.0 Hz, 1H, 1-H_R_), 4.07 (dd, *J*_6a,6b_ = 12.6, *J*_5,6a_ = 2.0 Hz, 1H, 6a-H_R_), 3.90 (dd, *J*_6a,6b_ = 12.7, *J*_5,6b_ = 4.8 Hz, 1H, 6b-H_R_), 3.76 (dd, *J*_1,2_ = 9.9, *J*_2,3_ = 8.7 Hz, 1H, 2-H_R_), 3.66–3.45 (m, 3H, 3-H_R_, 5-H_R,_ 4-H_R_ overlapping with 2-H_S_, 3-H_S_, 5-H_S,_ 4-H_S_ of 4i*S*) ppm. ^13^C NMR (100 MHz, D_2_O) data for *R* isomer: *δ* = 130.6–128.9 (aromatic_R_ overlapping with aromatic_S_ of 4i*S*), 86.8 (C-1_R_), 80.4 (C-5_R_), 76.9 (C-3_R_), 68.7 (C-4_R_), 67.6 (C-2_R_), 60.6 (C-6_R_), 51.6 (C-a_R_) ppm. HRMS (ESI): calcd for C_13_H_18_NaO_6_S 325.0716 [M + Na]^+^; found 325.0720.

## Conclusions

Photooxidation reactions of thiomonosaccharides under aerobic conditions were performed employing different organic dyes as sensitizers. Among the photocatalysts evaluated tetra-*O*-acetyl riboflavin provided good to excellent yields for the oxidation of alkyl, benzyl, and vinyl thioglycosides in considerably short reaction times. In addition, outstanding chemoselectivity towards the glycosyl sulfoxides without over-oxidation to sulfone was achieved. Furthermore, high selectivity was observed for the photooxidation of alkyl, allyl and benzyl thioglycosides as, under the same controlled conditions, phenyl thioglycosides are not oxidized. In the photooxidation reactions the formation of the sulfoxides with the S_*S*_ configuration was favored in almost all cases, due to the stereoselectivity generated by the asymmetric induction of the glucosyl residue.

The absolute configuration at the sulfur stereocenter of each sulfoxide was determined considering the shielding/deshielding effects generated by the anisotropy of the S=O bond in the chemical shift on the chemical shift of the ^1^H NMR signals of specific protons, by the anisotropy of the S=O bond. These effects were analyzed for the preferential rotational conformers (g+ and/or g−) for each diastereoisomer, which were confirmed by the presence of characteristic NOE contacts in the NOESY spectra. On the basis of all these data the S_*S*_ or S_*R*_ configurations were assigned.

This photosensitized oxidation reaction, employing visible light under aerobic conditions, constitutes a remarkably simple, efficient, and economical methodology to obtain glycosyl sulfoxides. In addition, it is an environmentally friendly process since high atom economy is achieved. The desired sulfoxides were isolated after a rather simple purification process due to the use of catalytic amounts of organic dyes together with the high chemoselectivity observed.

## Conflicts of interest

There are no conflicts to declare.

## Supplementary Material

RA-011-D0RA09534F-s001
