# Supplementary material for: Photooxidation of thiosaccharides mediated by sensitizers in aerobic and environmentally friendly conditions
Source: RSC Adv. 2021 Mar 1;11(16):9262–73. doi: 10.1039/d0ra09534f (PMC8695230; doi:10.1039/d0ra09534f)

Electronic Supplementary Material (ESI) for RSC Advances.
This journal is © The Royal Society of Chemistry 2021

Supporting Information for

Photooxidation of Thiosaccharides Mediated by Sensitizers in Aerobic and Environmentally Friendly Conditions

Miqueas G. Traverssi[a,b], Alicia B. Peñéñory[a,b], Oscar Varela[c,d] and Juan P. Colomer[a,b]*

[a]*Universidad Nacional de Córdoba. Facultad Ciencias Químicas, Departamento de Química Orgánica. Ciudad Universitaria, Edificio de Ciencias II, Córdoba, Argentina.*
[b]*Consejo Nacional de Investigaciones Científicas y Técnicas (CONICET)-UNC Instituto de Investigaciones en Fisico-Química de Córdoba (INFIQC).*
[c]*Universidad de Buenos Aires. Facultad Ciencias Exactas y Naturales, Departamento de Química Orgánica. Ciudad Universitaria, Pab. 2, C1428EHA, Buenos Aires, Argentina.*
[d]*Consejo Nacional de Investigaciones Científicas y Técnicas (CONICET)-UBA Centro de Investigación en Hidratos de Carbono (CIHIDECAR).*

Index

^{1}H, ^{13}C NMR Spectra of acetyl thiosaccharides **3a-f** …………………………….….. S2-S7

^{1}H, ^{13}C NMR Spectra of free thiosaccharides **3g-k** ……………………………….…..... S8-S12

^{1}H, ^{13}C, NOESY NMR Spectra of acetyl sulfoxides **4a-d** …………………………... S13-S20

^{1}H, ^{13}C, NOESY NMR Spectra of free sulfoxides **4g-i** …...………….……..……... S20-S24

Oxidation reaction of free thiosaccharide **3i** peracetylated …………………………. S25

^{1}H NMR of compound **3a**

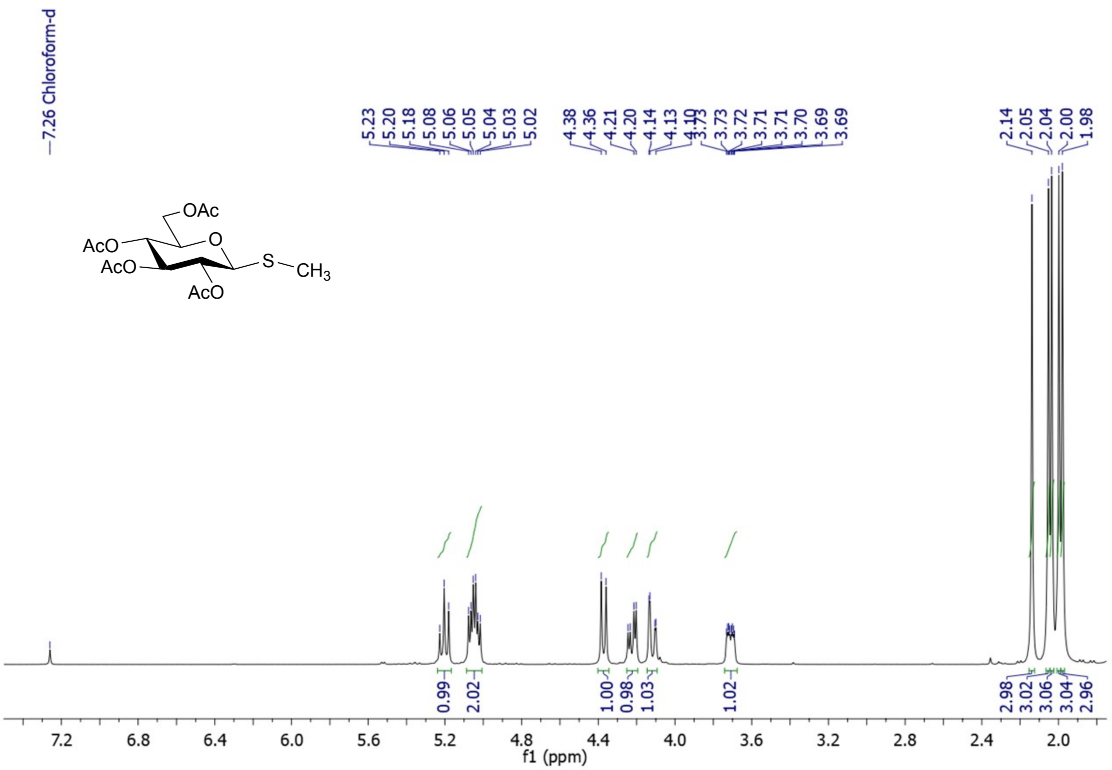

^{13}C NMR of compound **3a**

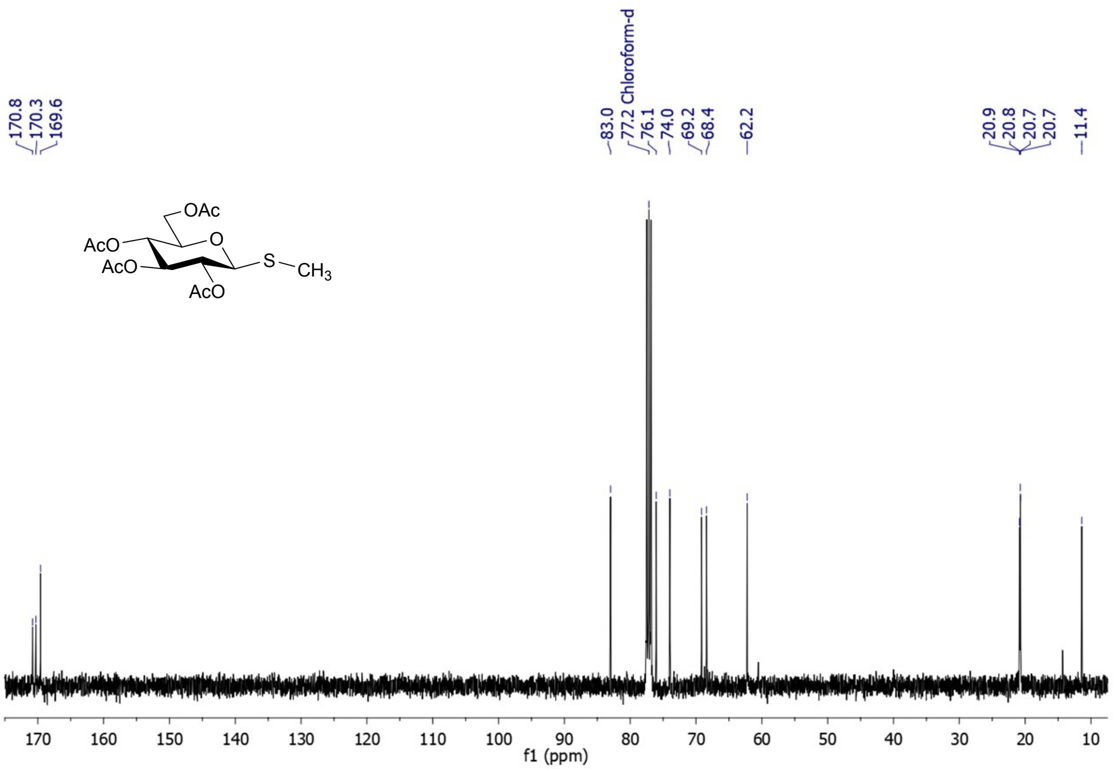

^{1}H NMR of compound **3b**

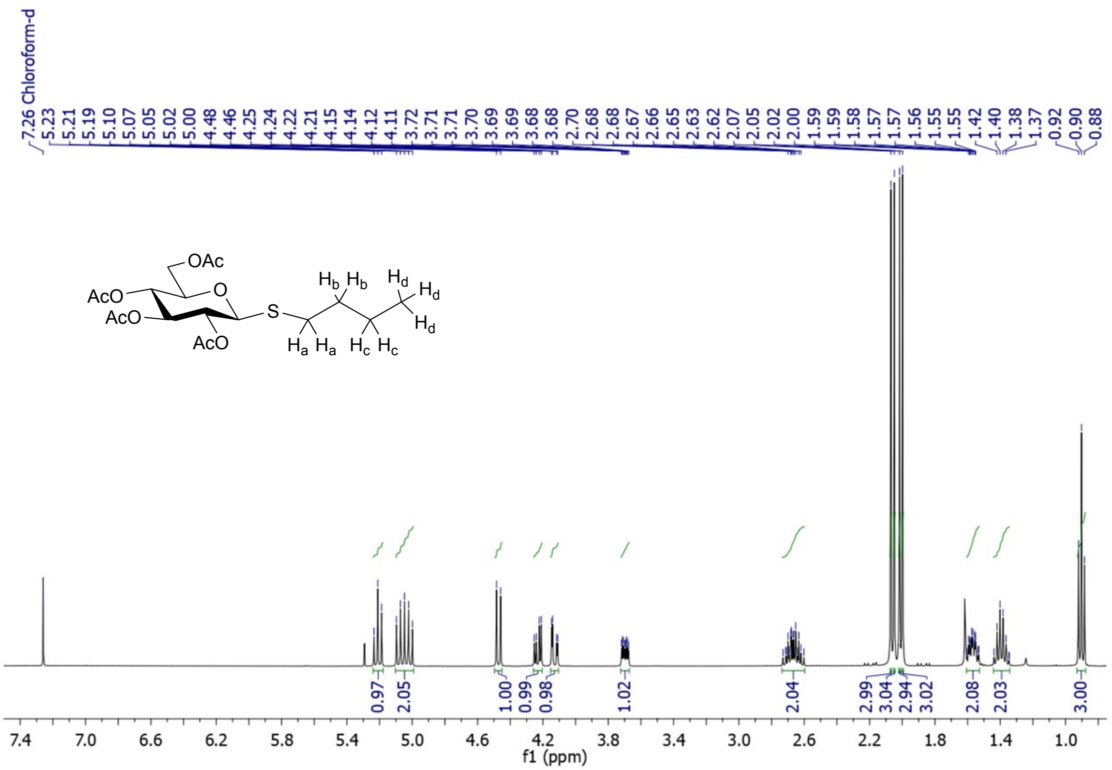

^{13}C NMR of compound **3b**

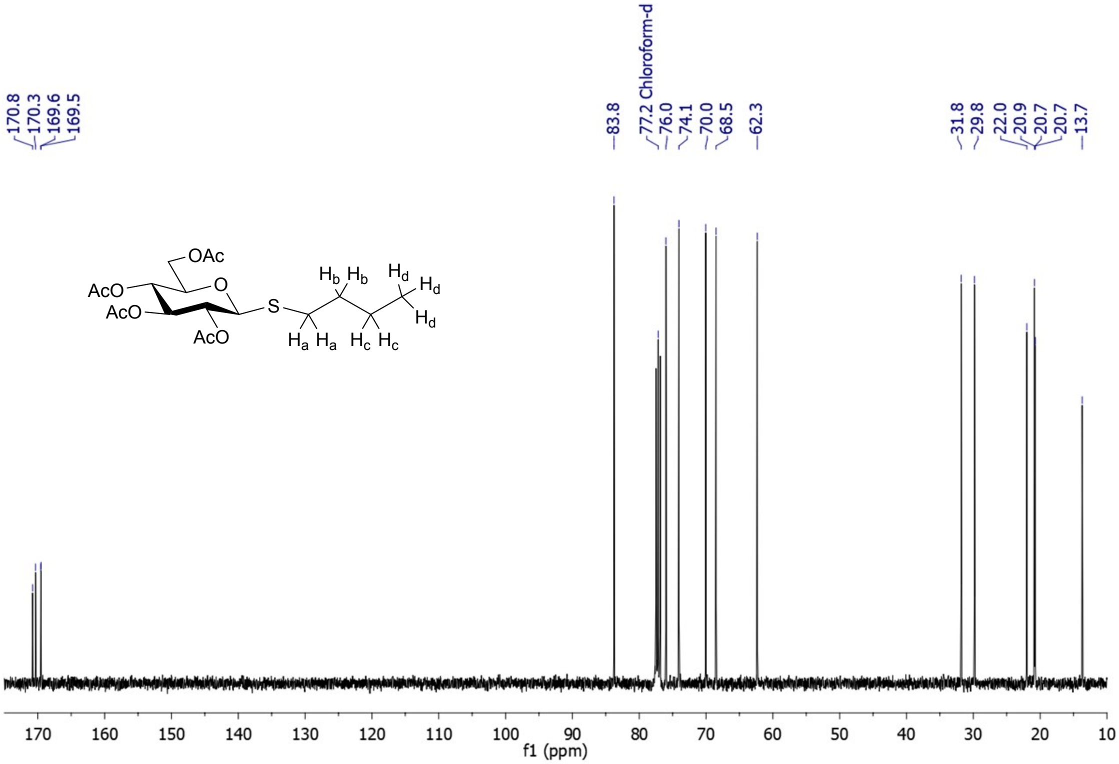

^{1}H NMR of compound **3c**

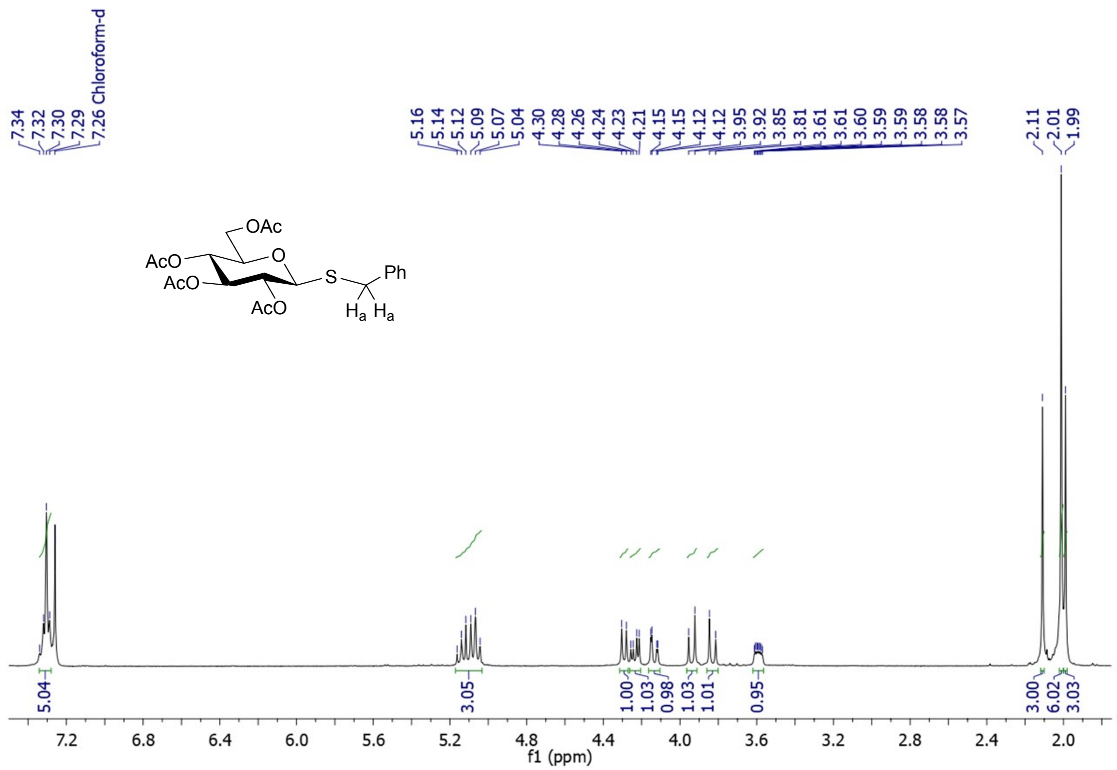

^{13}C NMR of compound **3c**

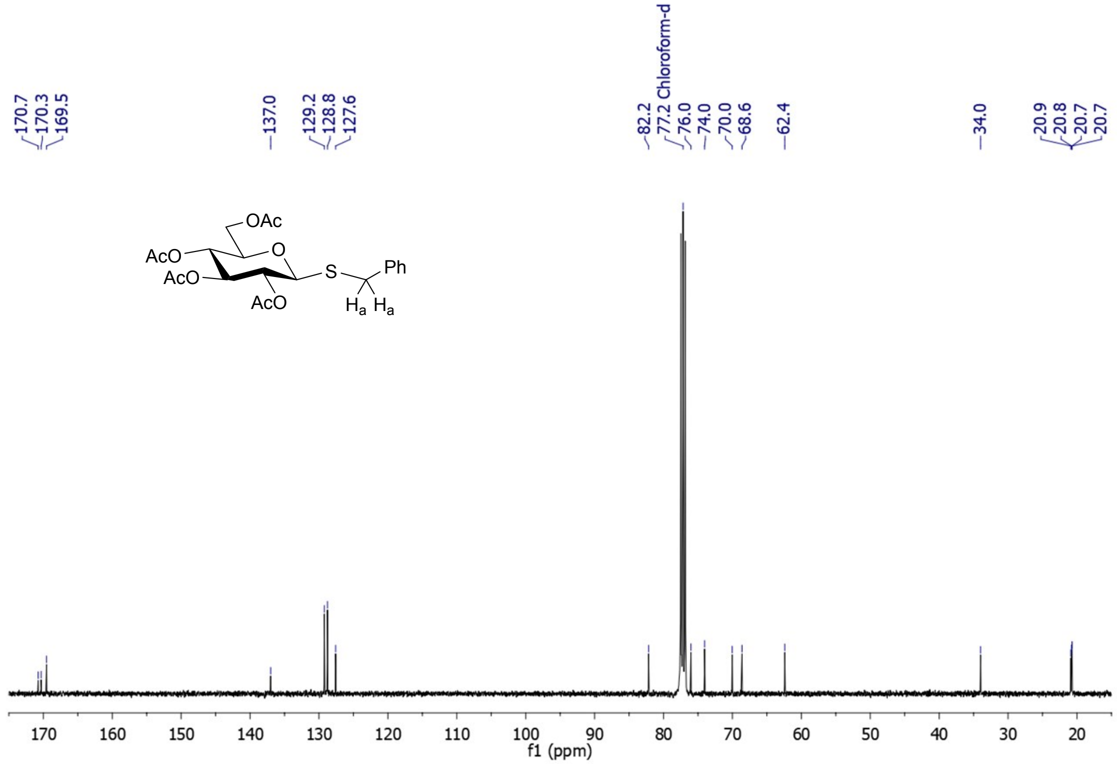

^{1}H NMR of compound **3d**

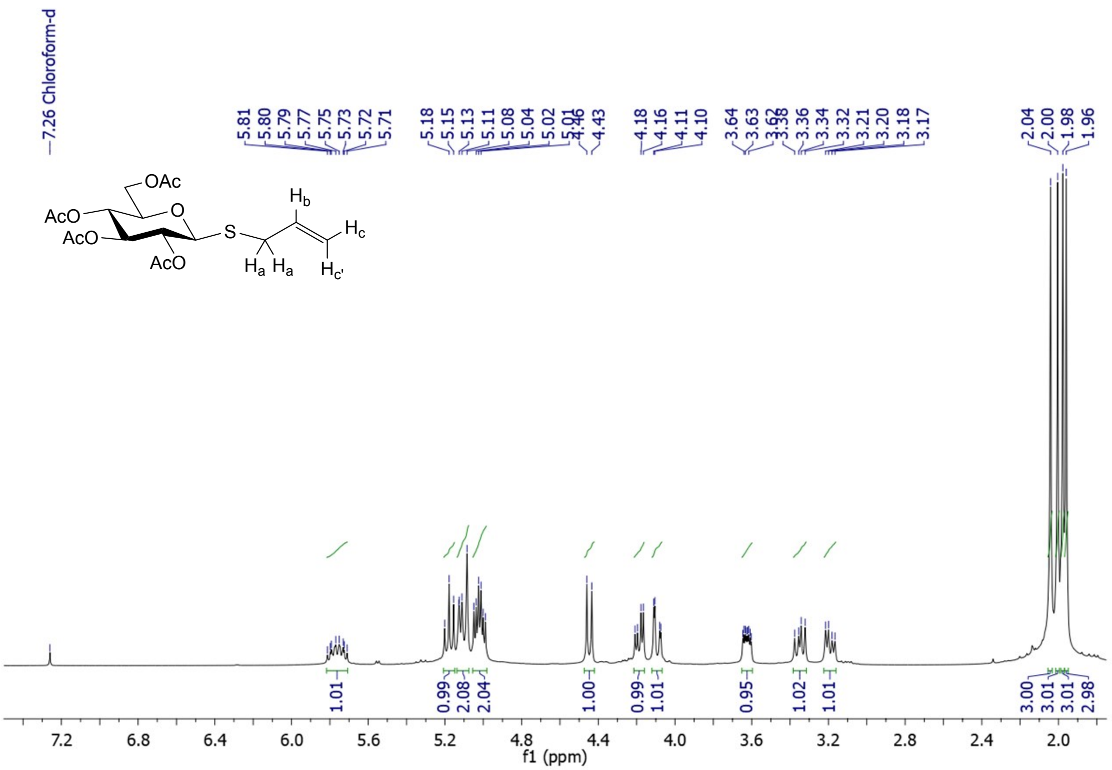

^{13}C NMR of compound **3d**

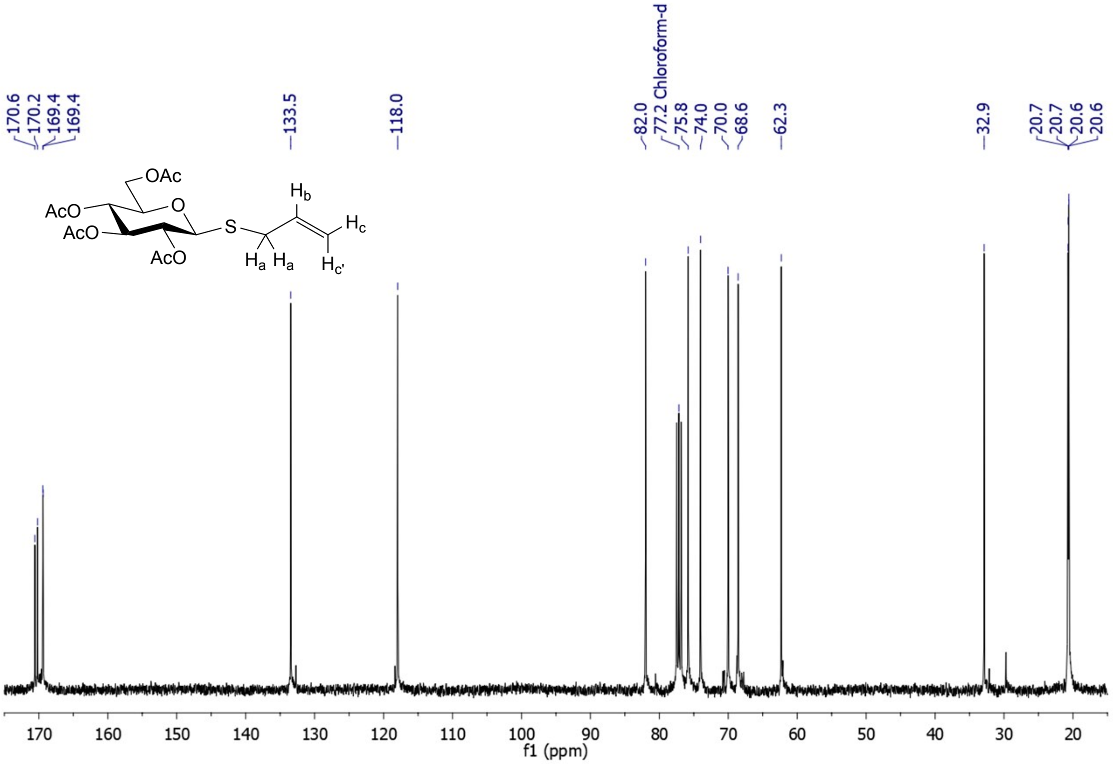

^{1}H NMR of compound **3e**

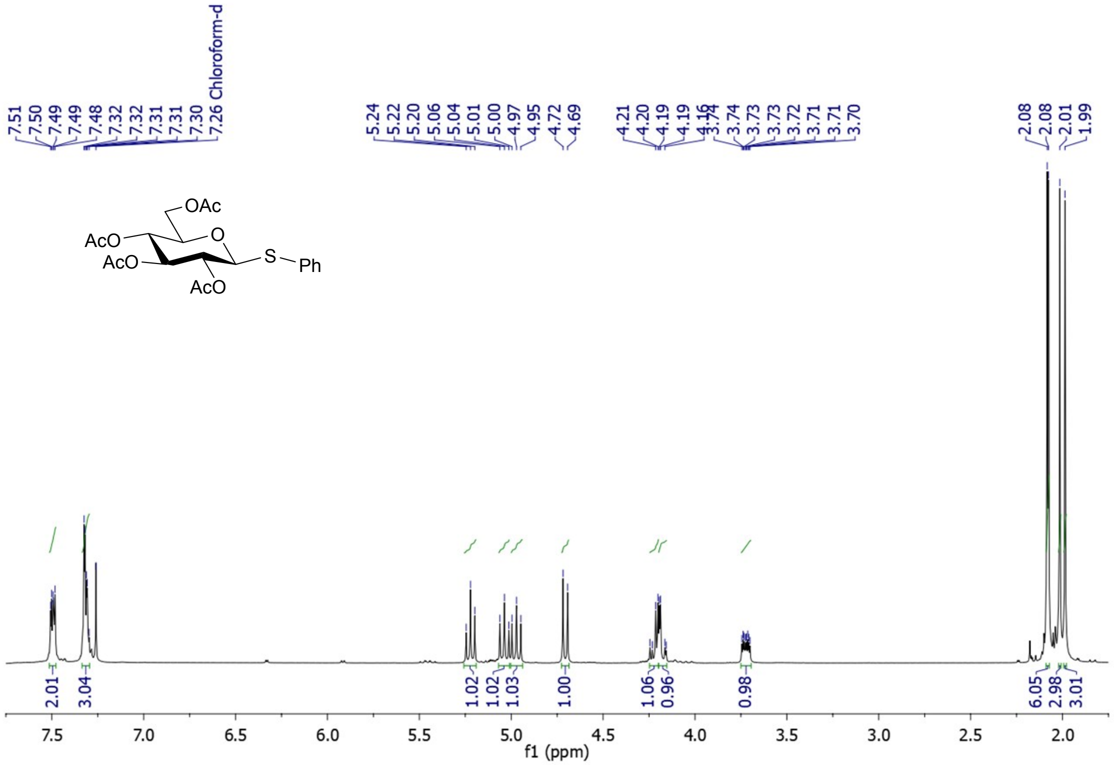

^{13}C NMR of compound **3e**

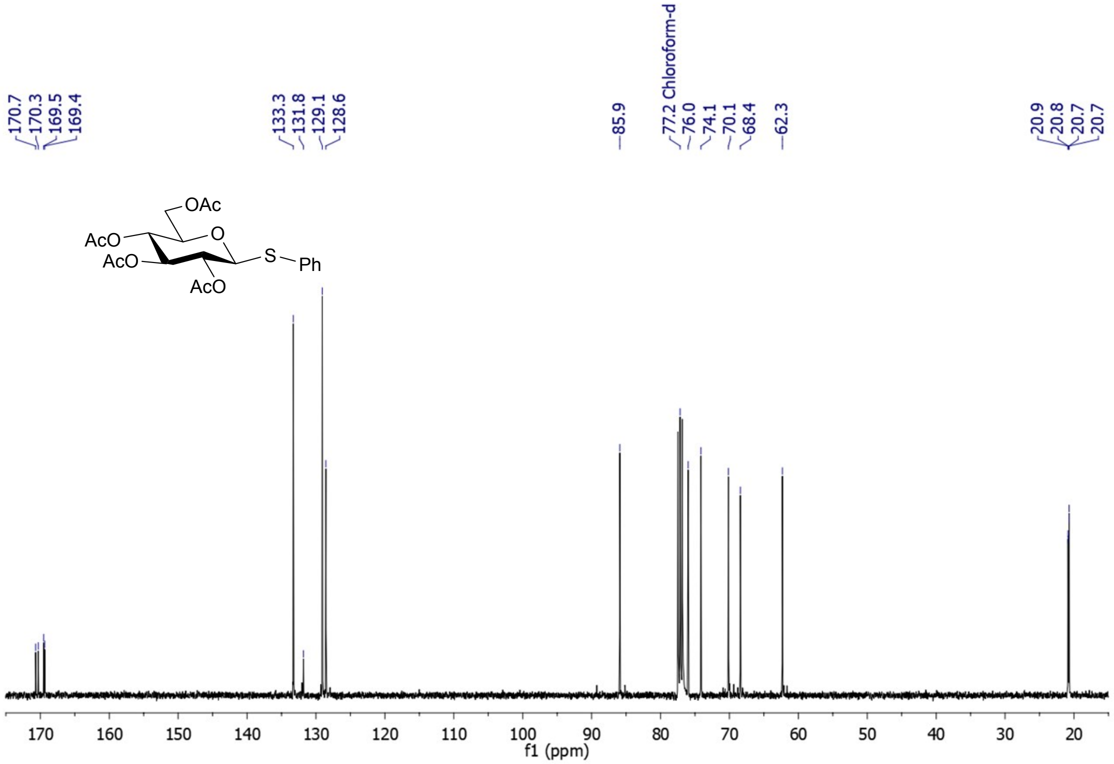

^{1}H NMR of compound **3f**

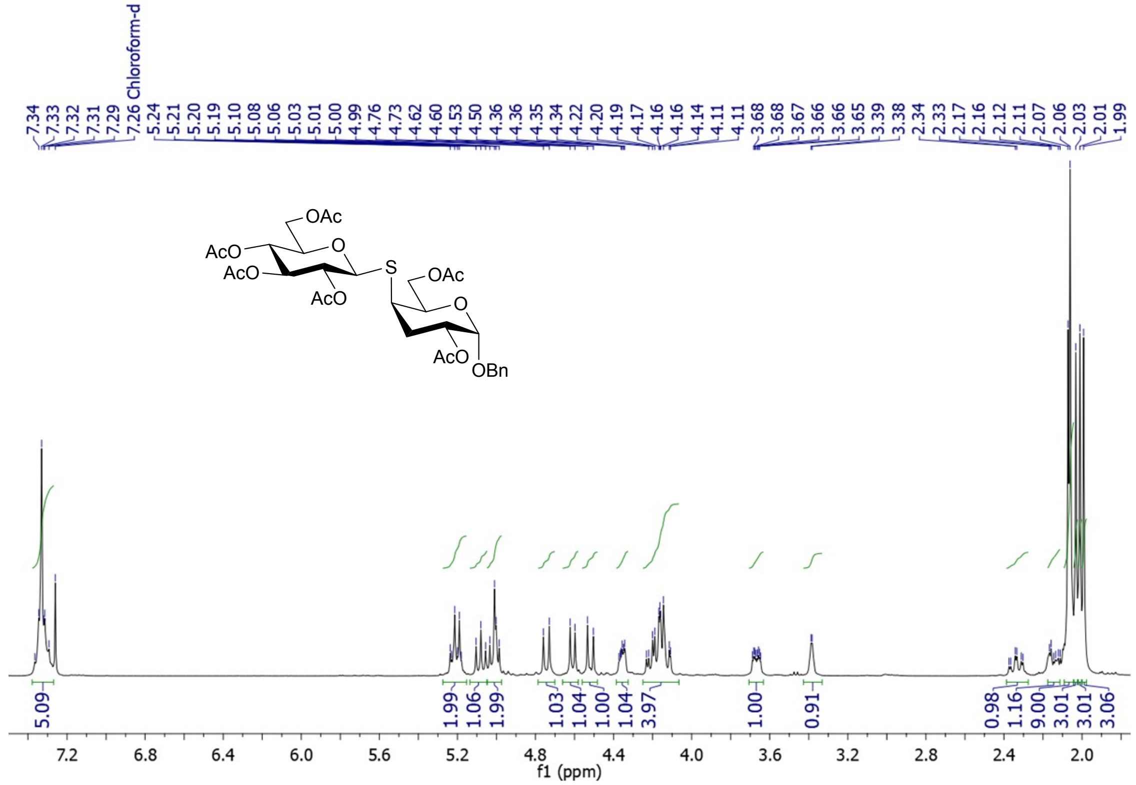

^{13}C NMR of compound **3f**

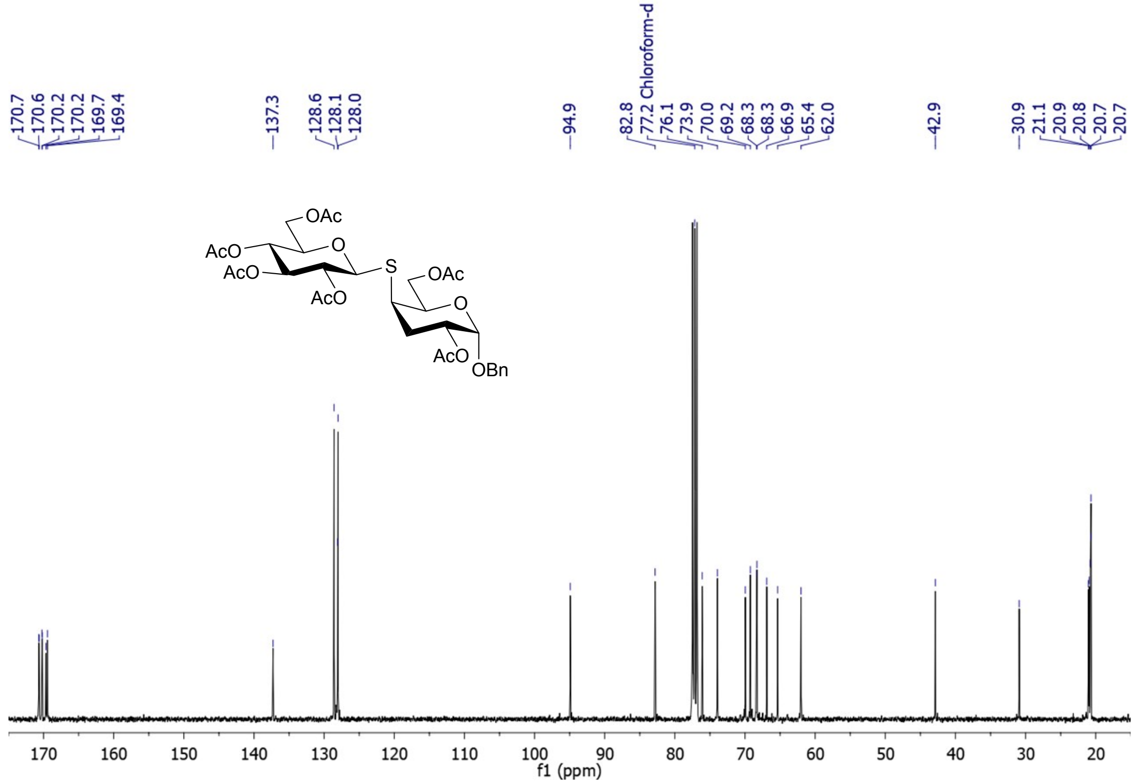

^{1}H NMR of compound **3g**

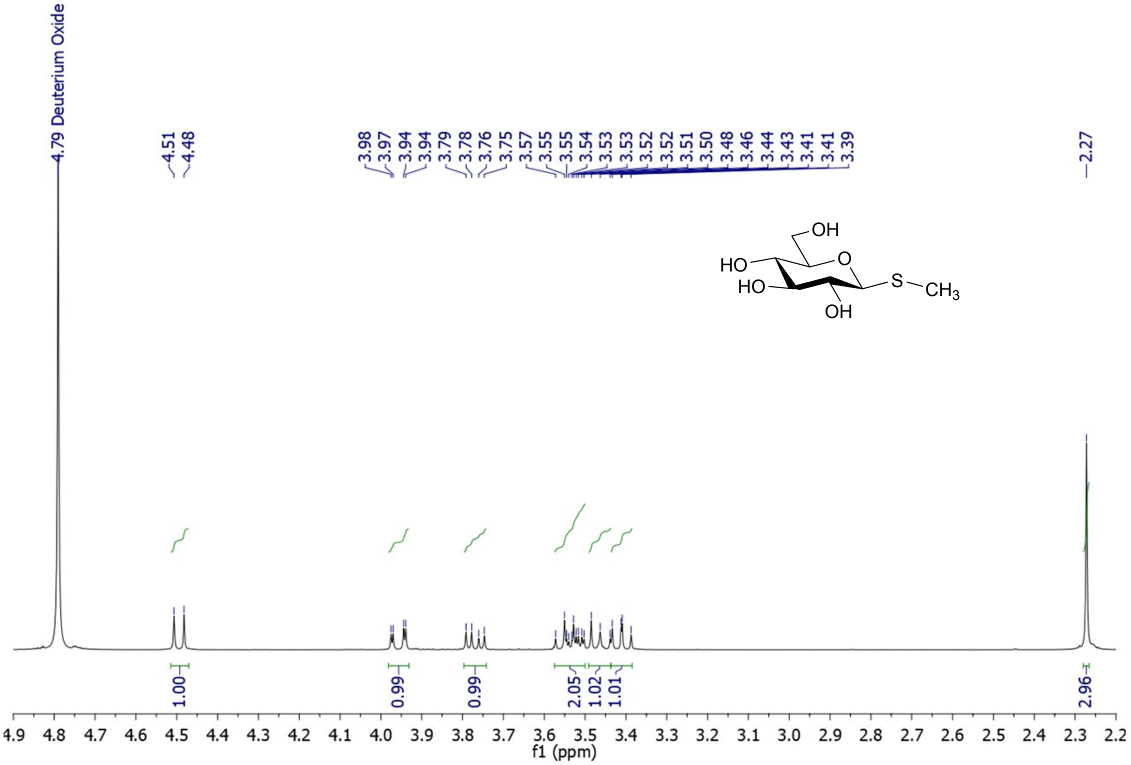

^{13}C NMR of compound **3g**

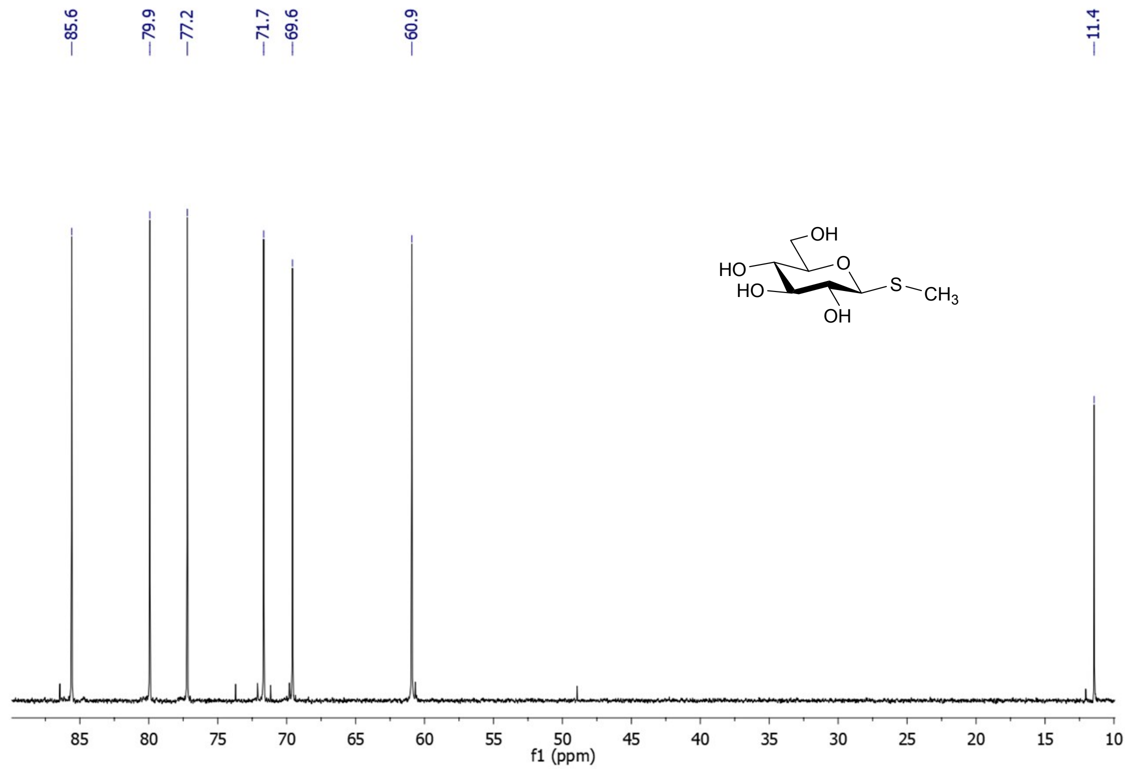

^{1}H NMR of compound **3h**

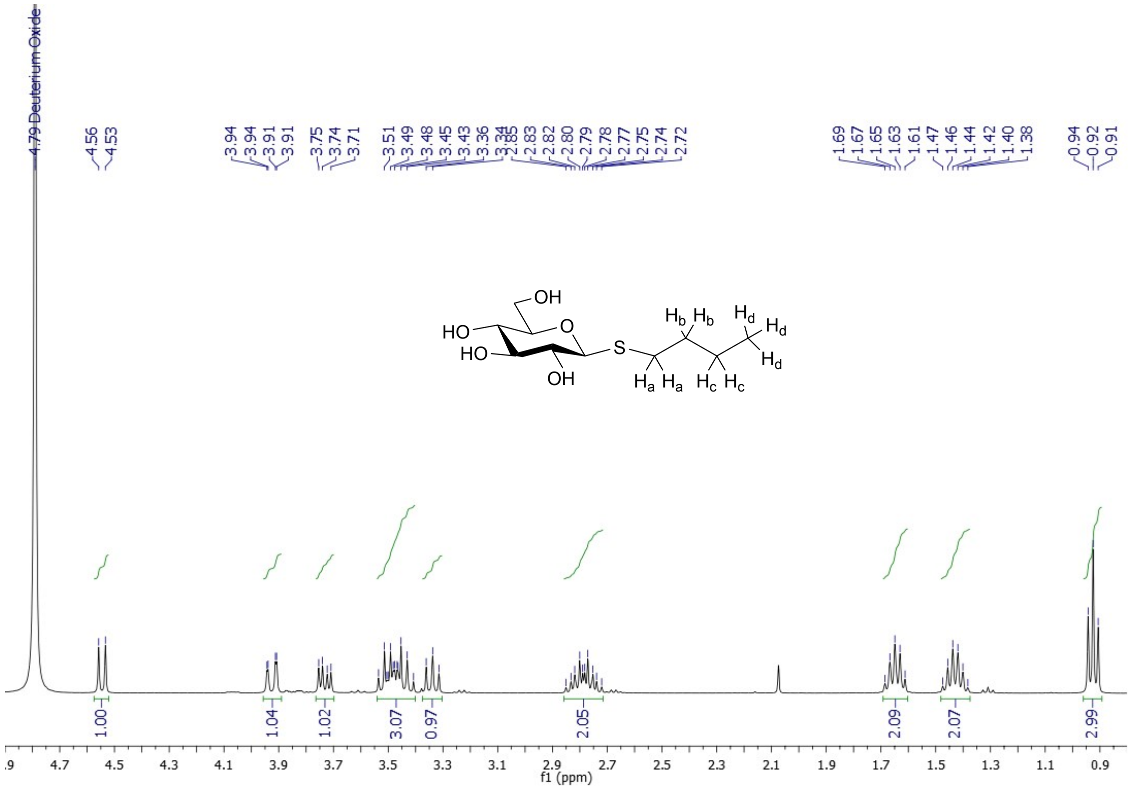

^{13}C NMR of compound **3h**

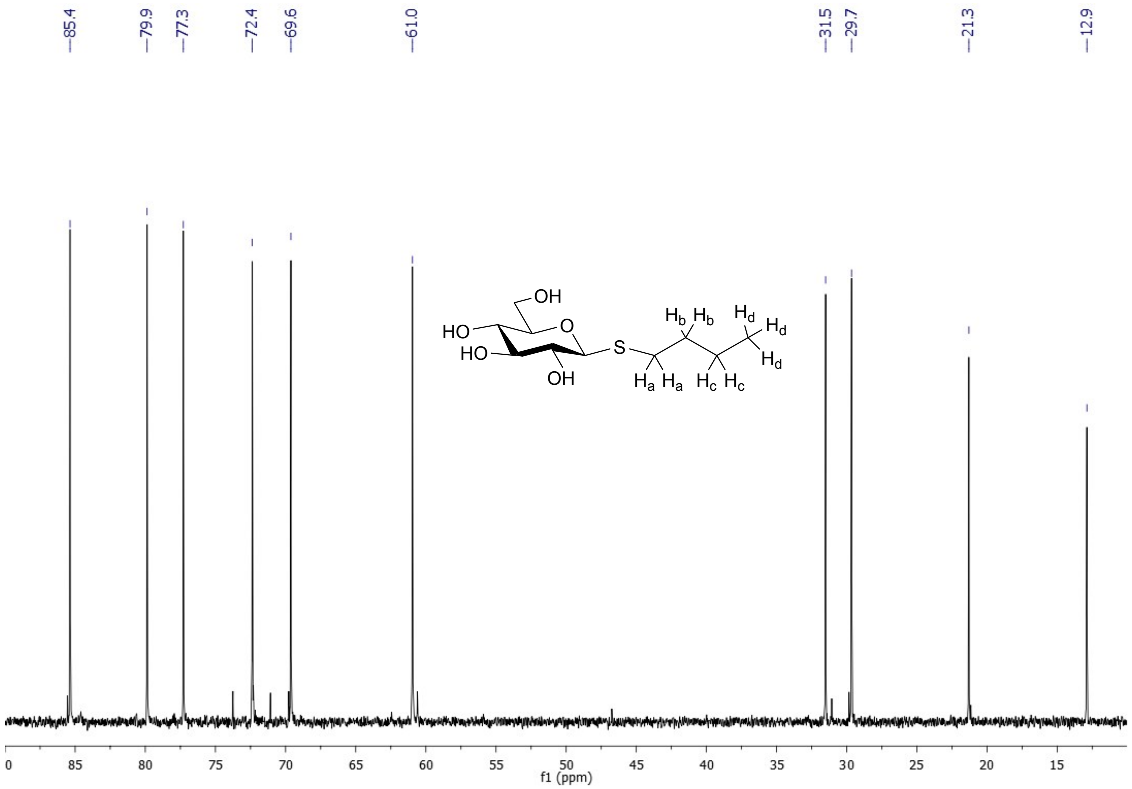

^{1}H NMR of compound **3i**

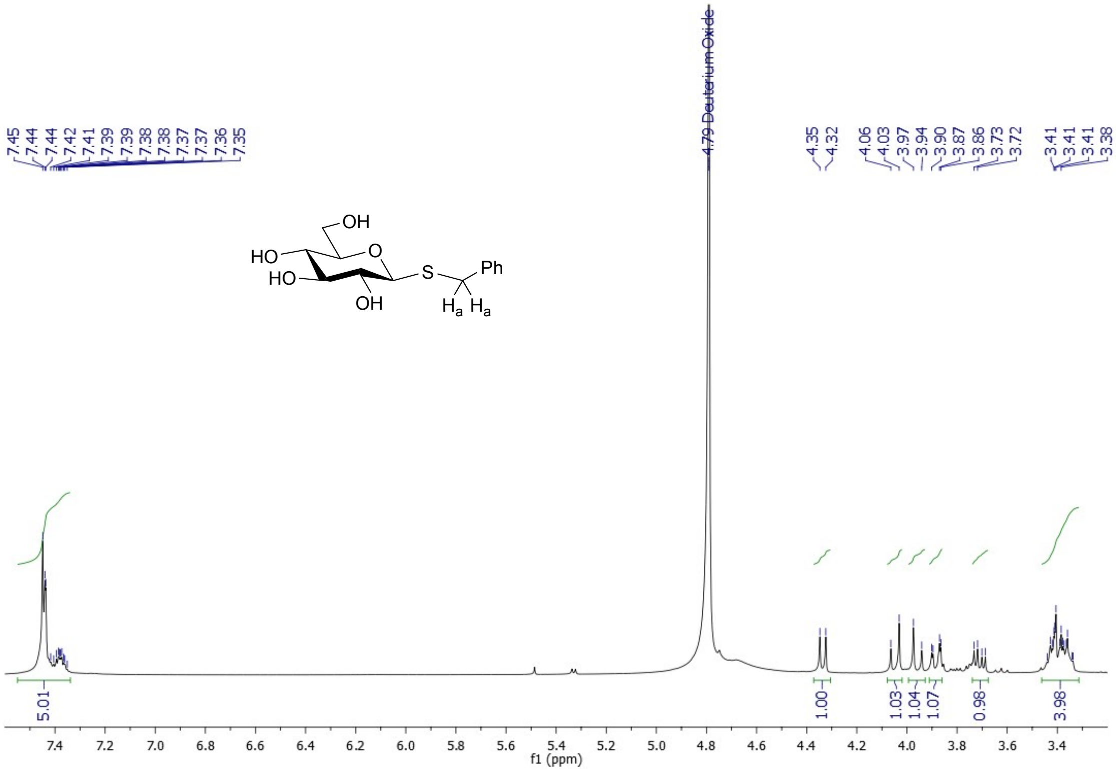

^{13}C NMR of compound **3i**

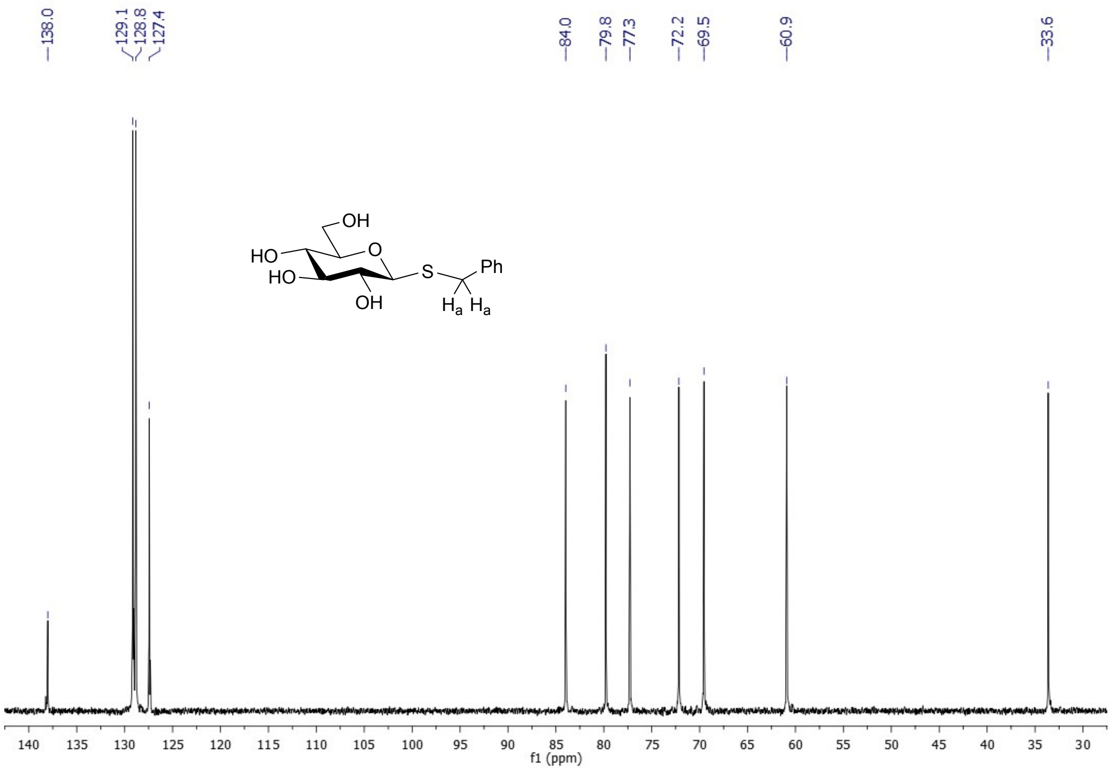

^{1}H NMR of compound **3j**

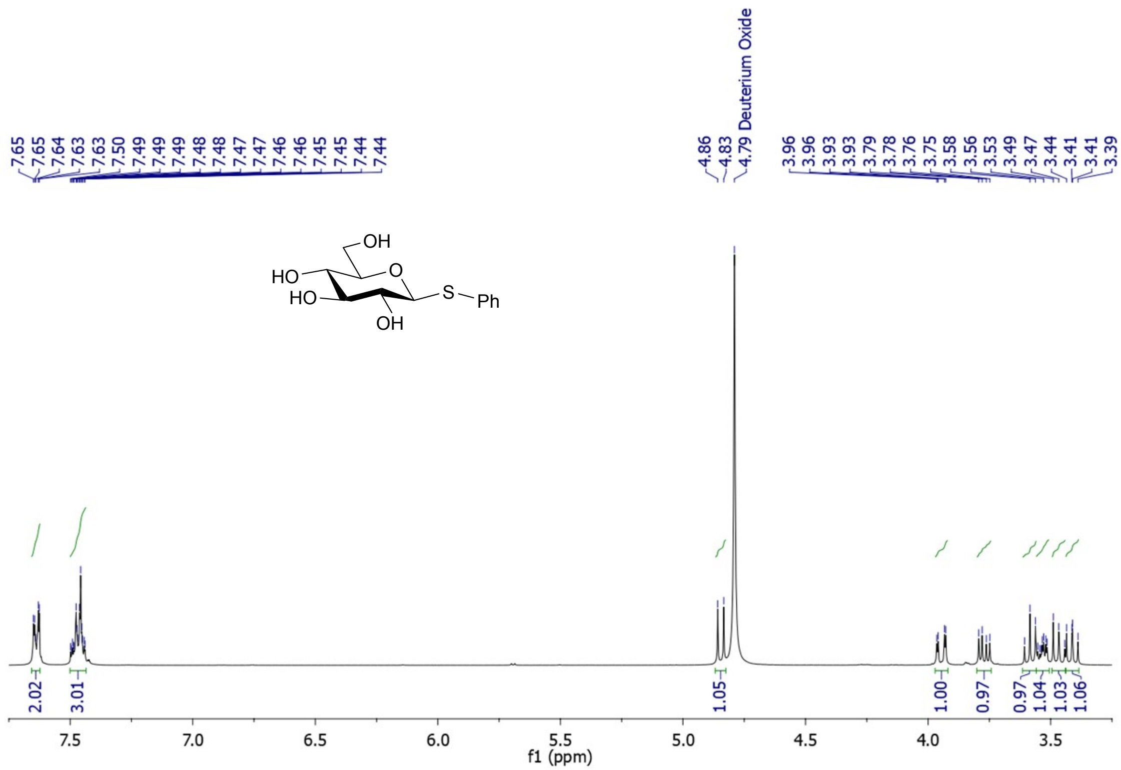

^{13}C NMR of compound **3j**

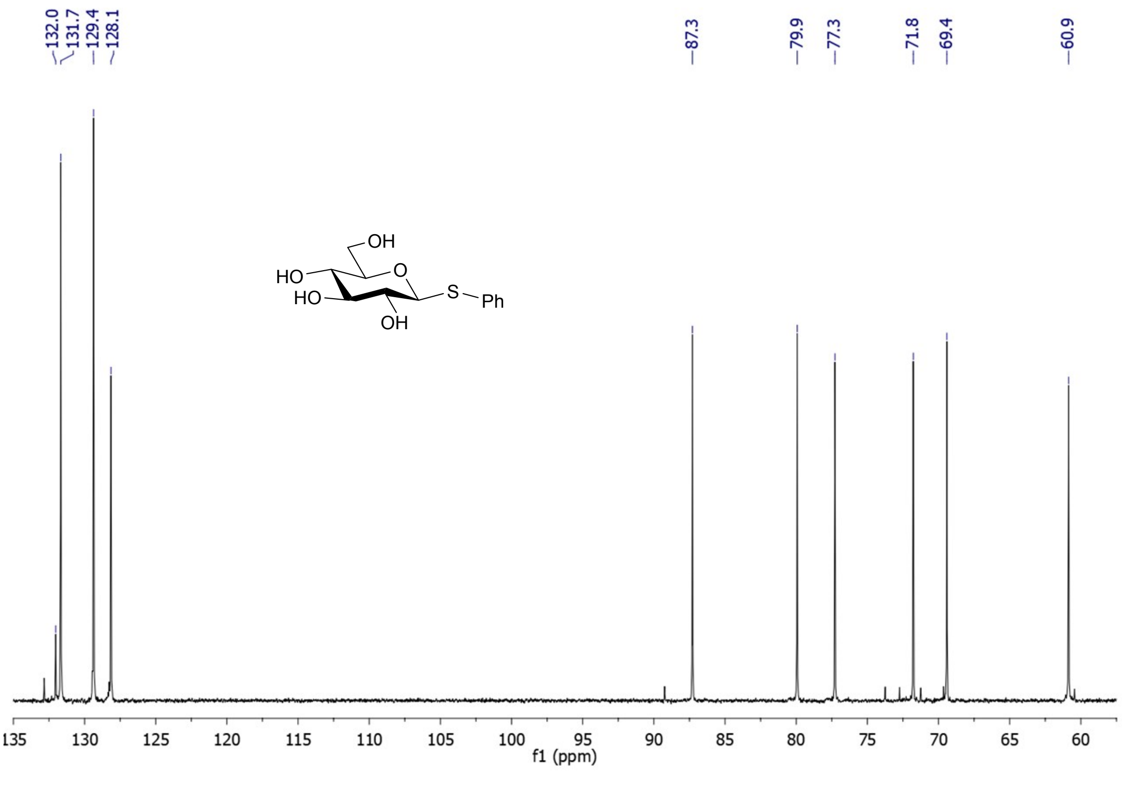

^{1}H NMR of compound **3k**

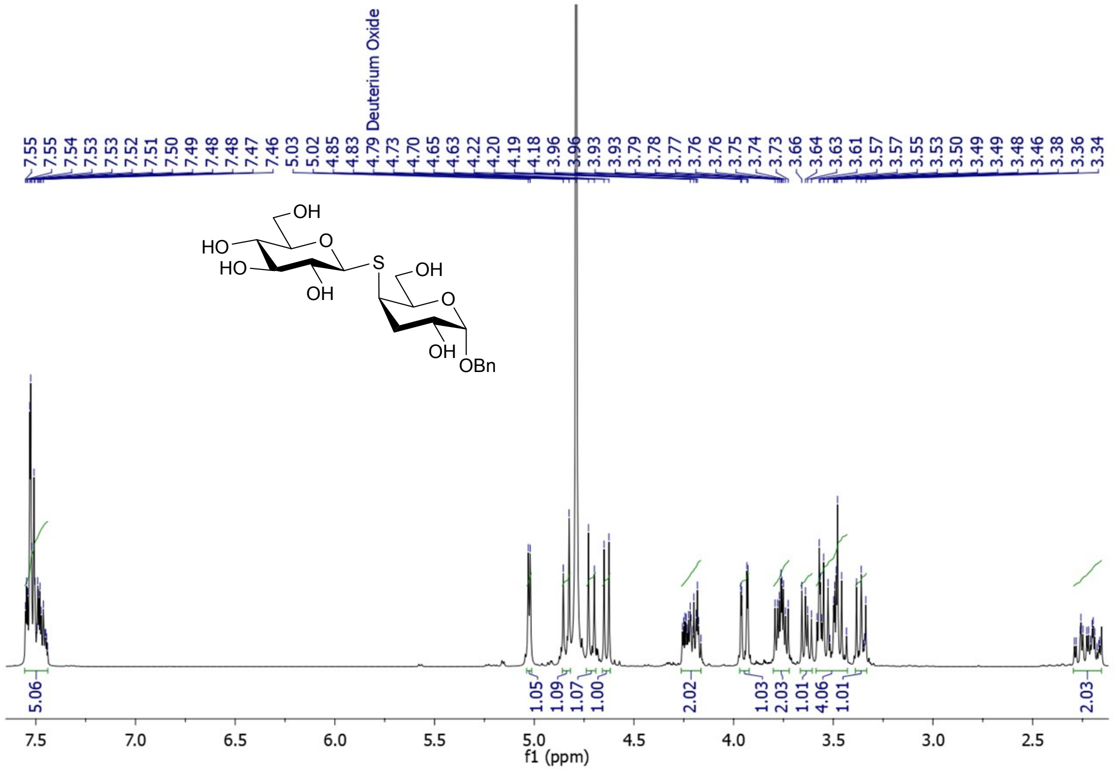

^{13}C NMR of compound **3k**

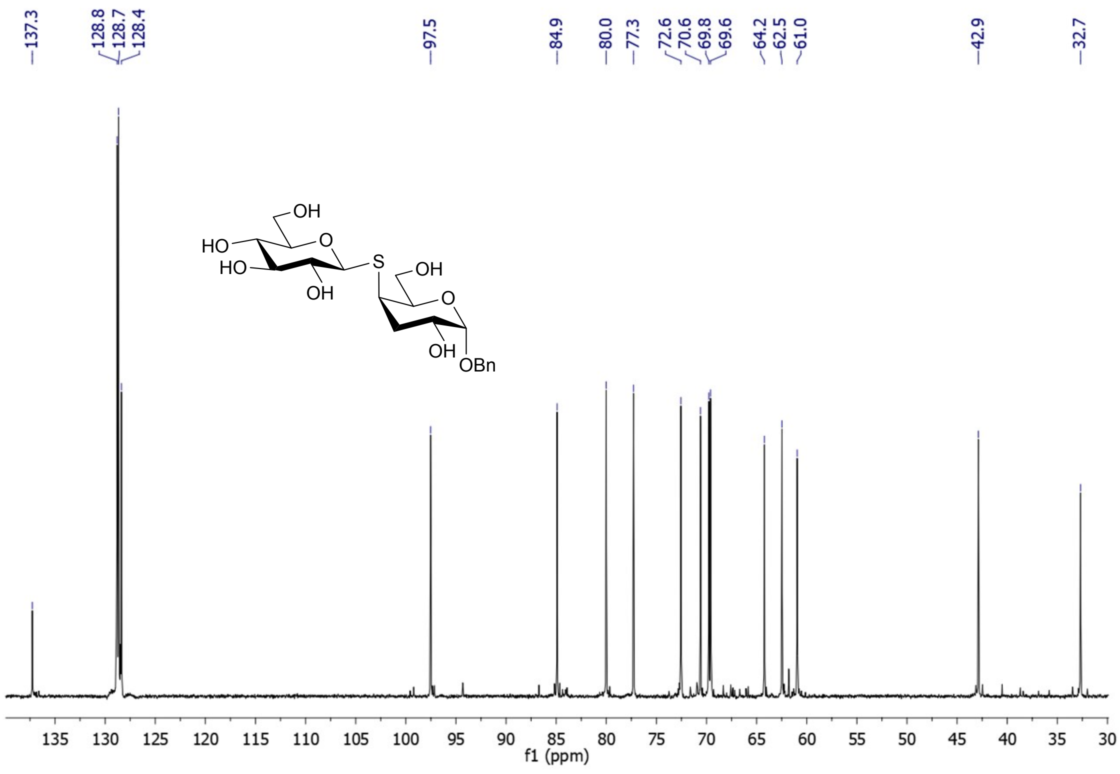

^{1}H NMR of compound **4a*R***

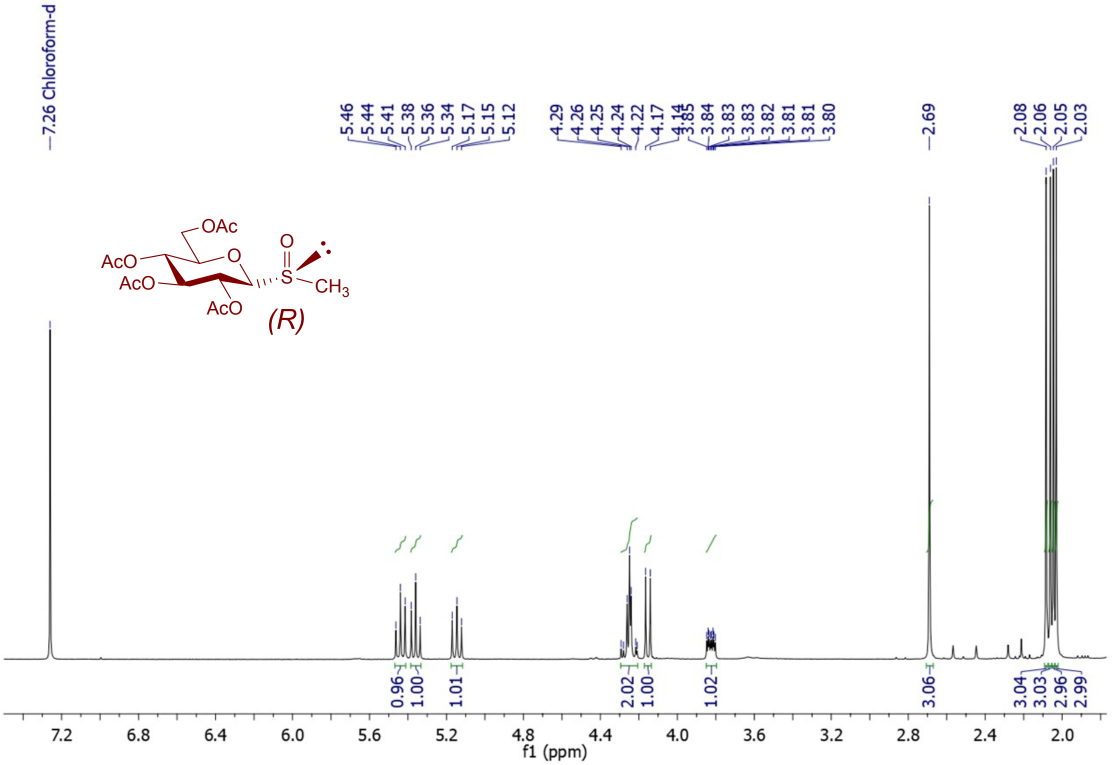

^{13}C NMR of compound **4a*R***

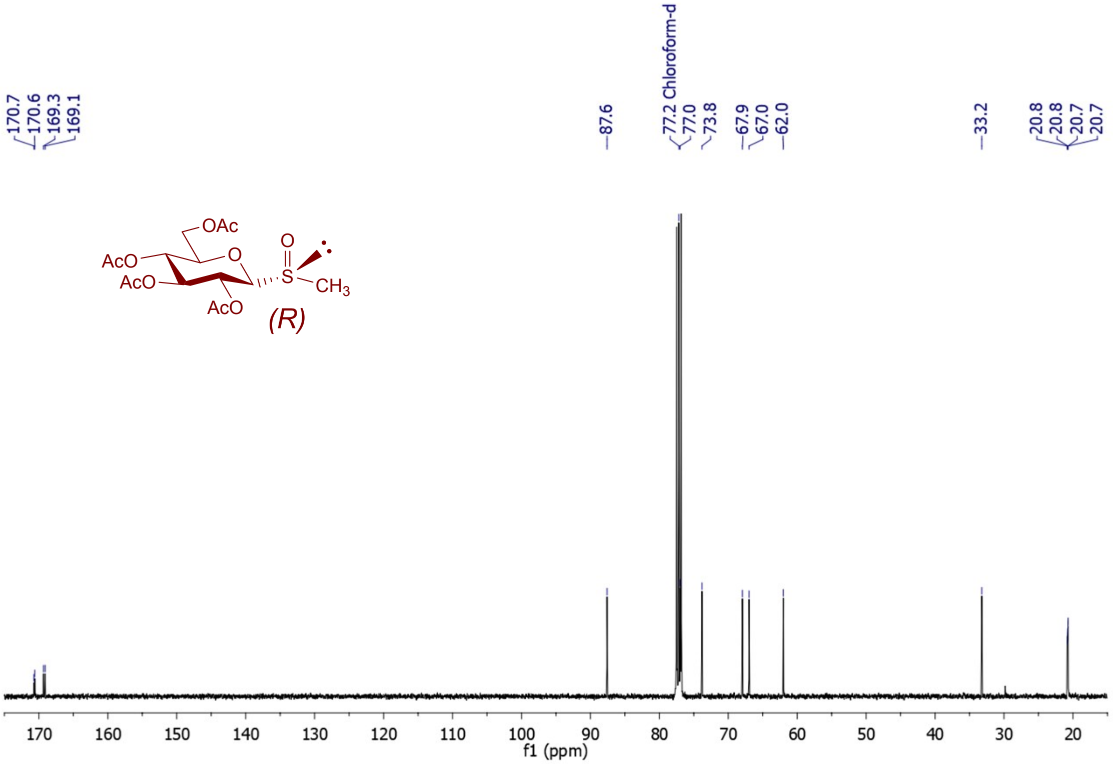

NOESY of compound **4a*R***

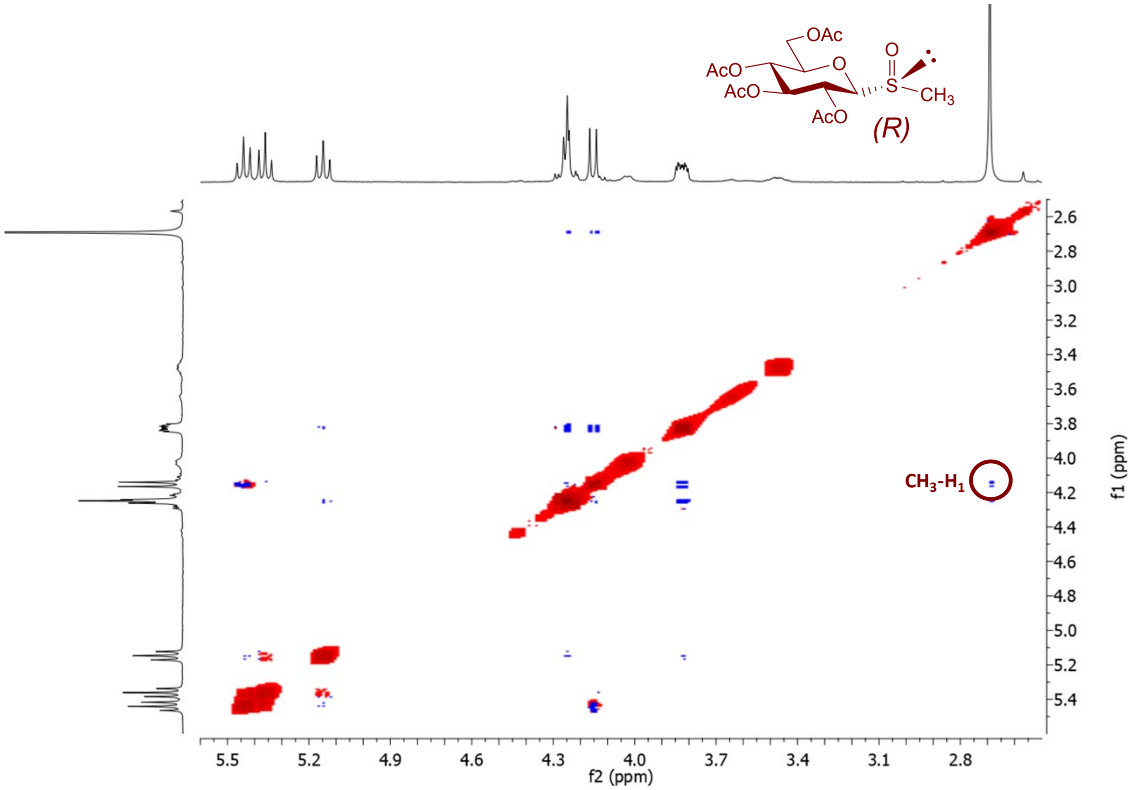

^{1}H NMR of compound **4a*S***

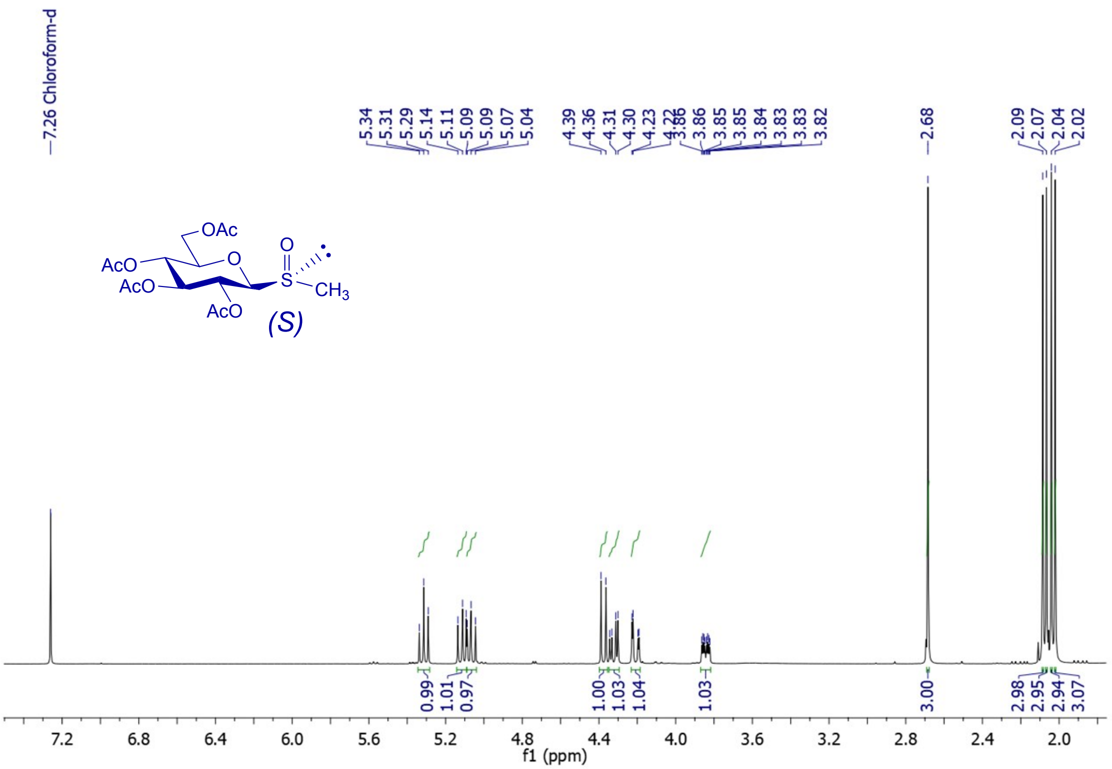

^{13}C NMR of compound **4a*S***

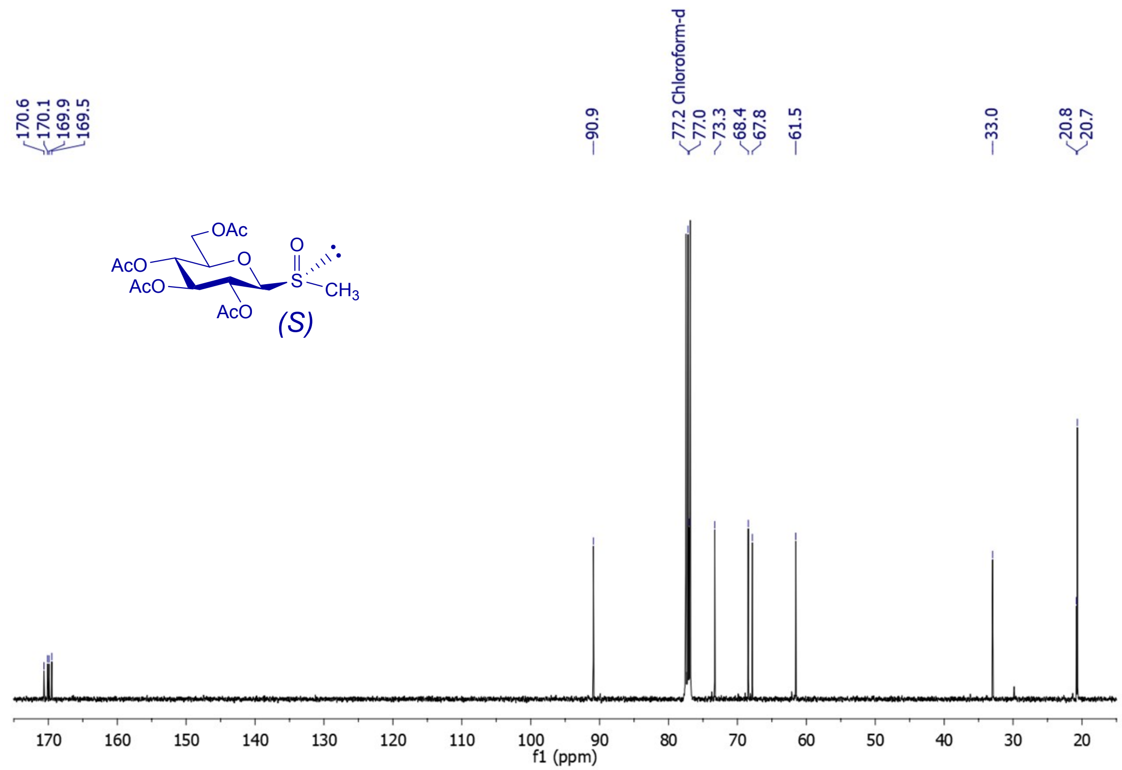

NOESY of compound **4a*S***

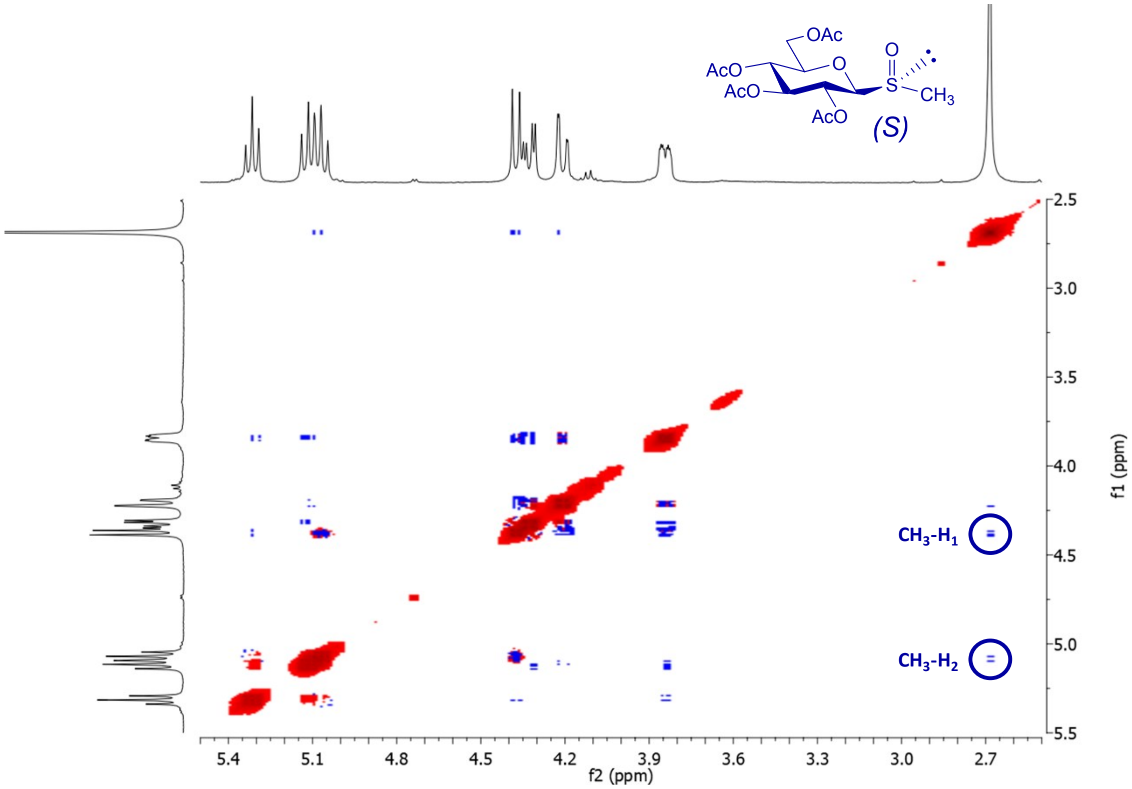

^{1}H NMR of compounds **4b*R*** and **4b*S***

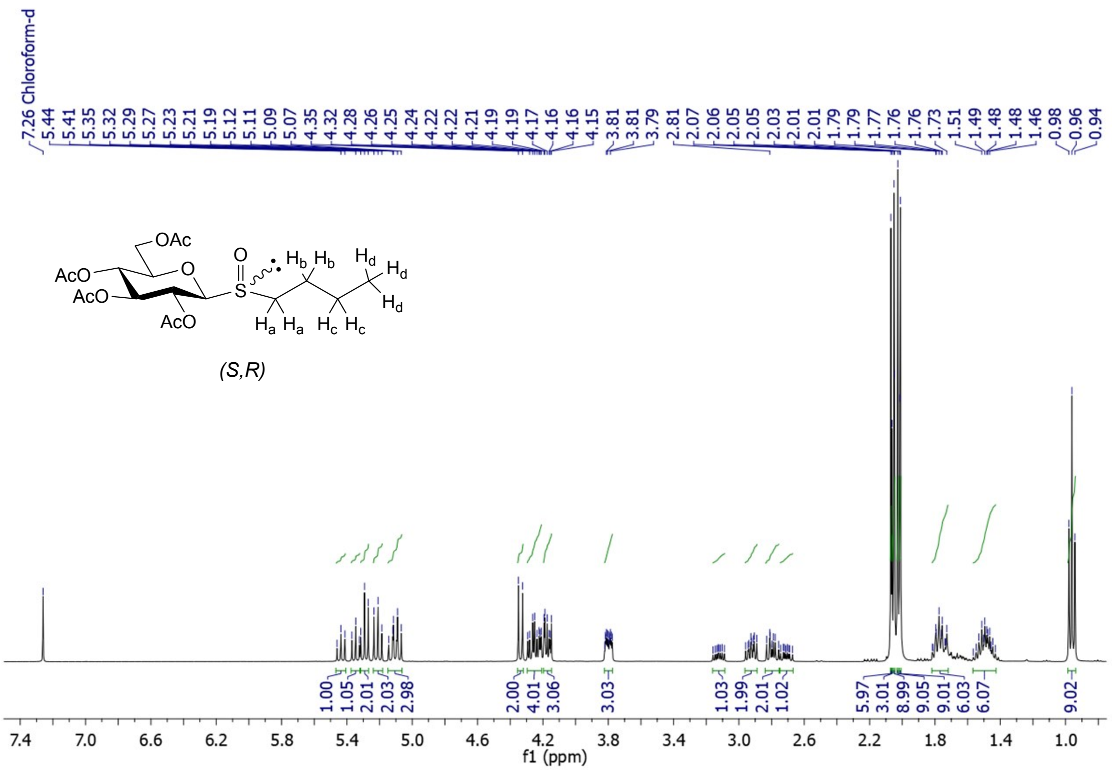

^{13}C NMR of compounds **4b*R*** and **4b*S***

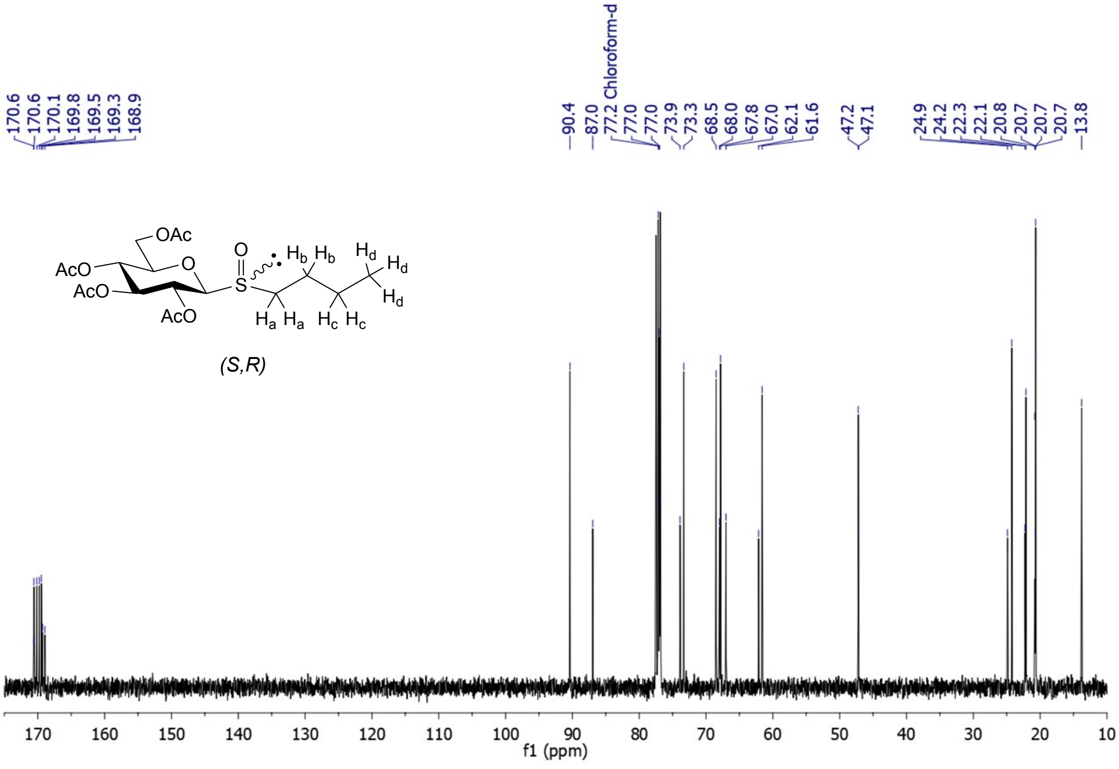

NOESY of compounds **4b*R*** and **4b*S***

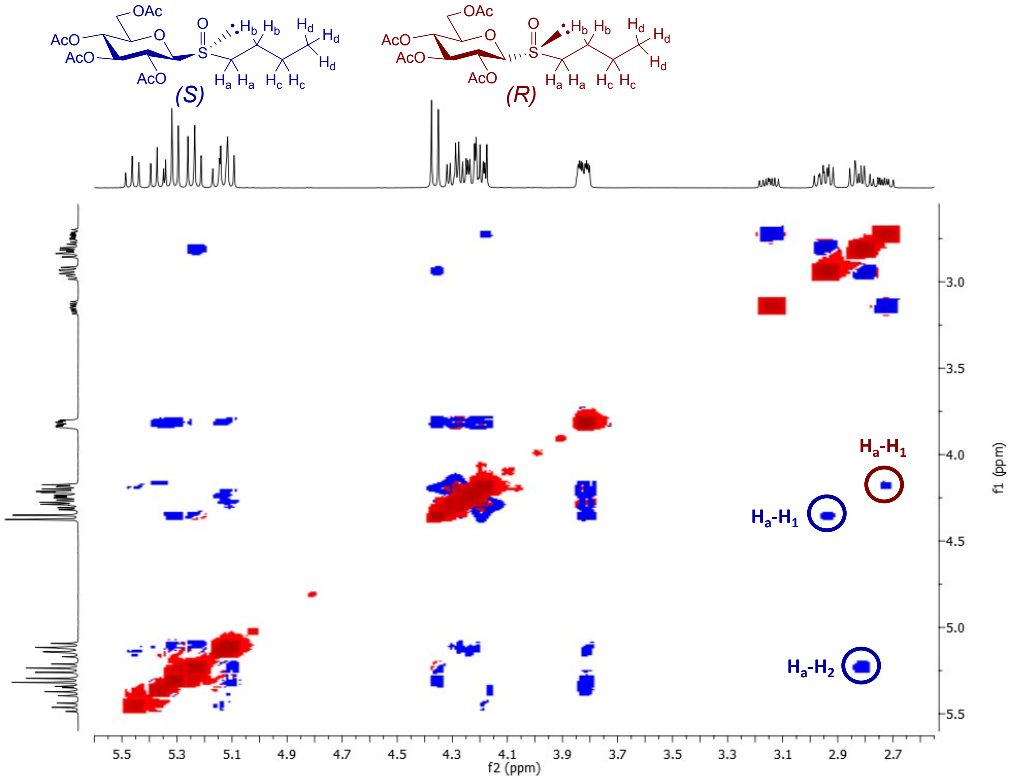

^{1}H NMR of compounds **4c*R*** and **4c*S***

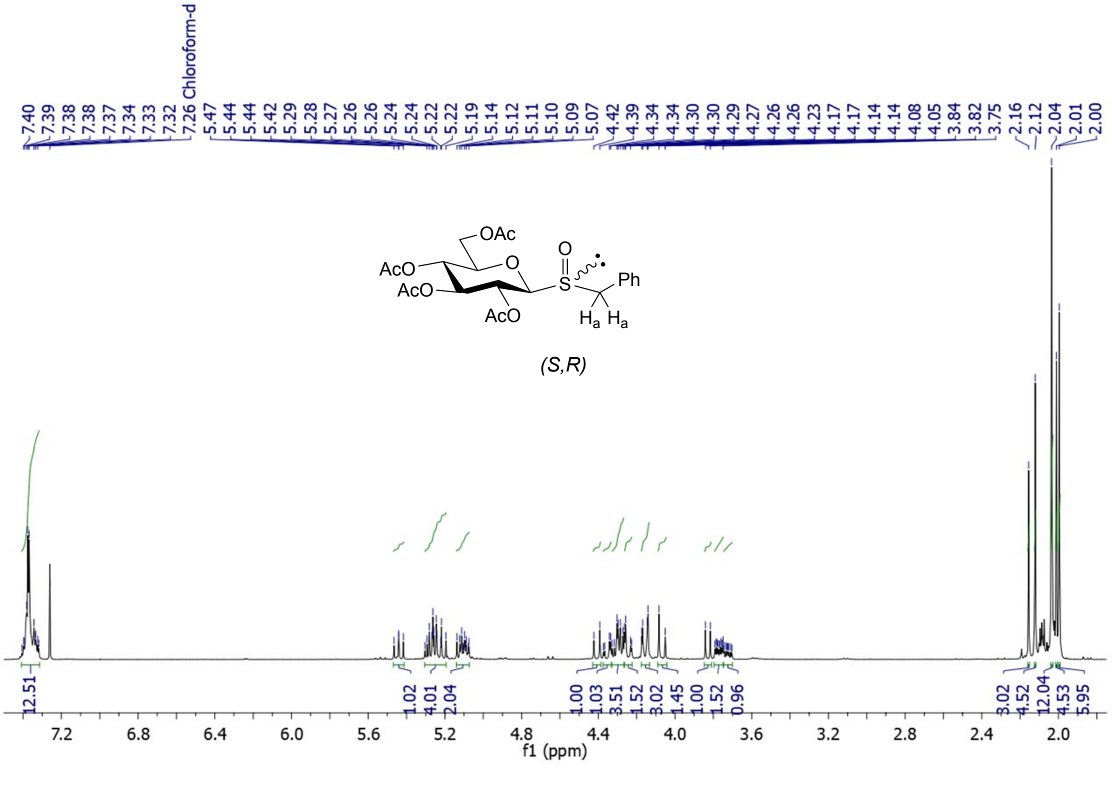

^{13}C NMR of compounds **4c*R*** and **4c*S***

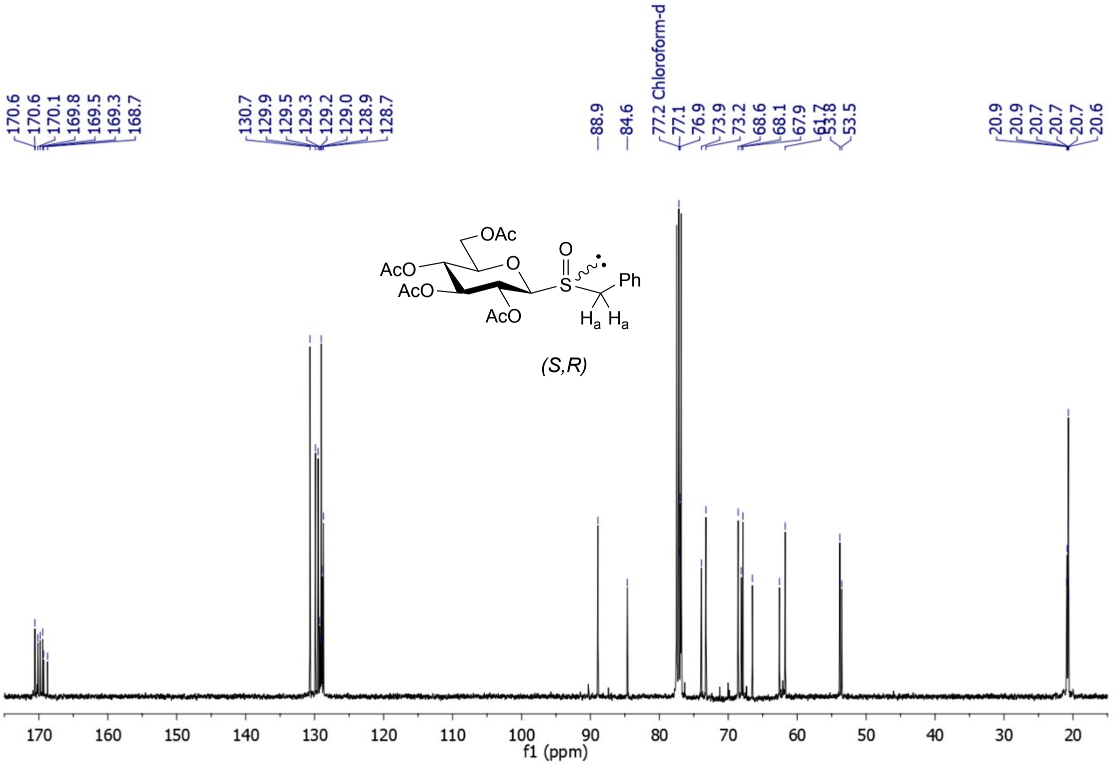

NOESY of compounds **4c*R*** and **4c*S***

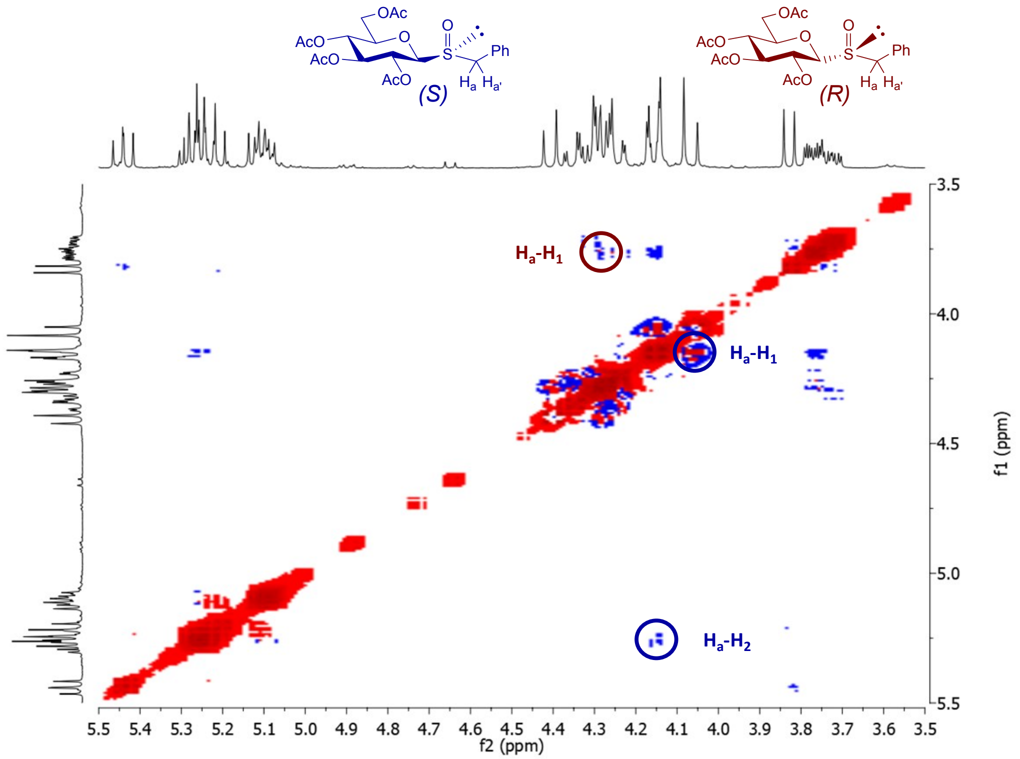

^{1}H NMR of compounds **4d*R*** and **4d*S***

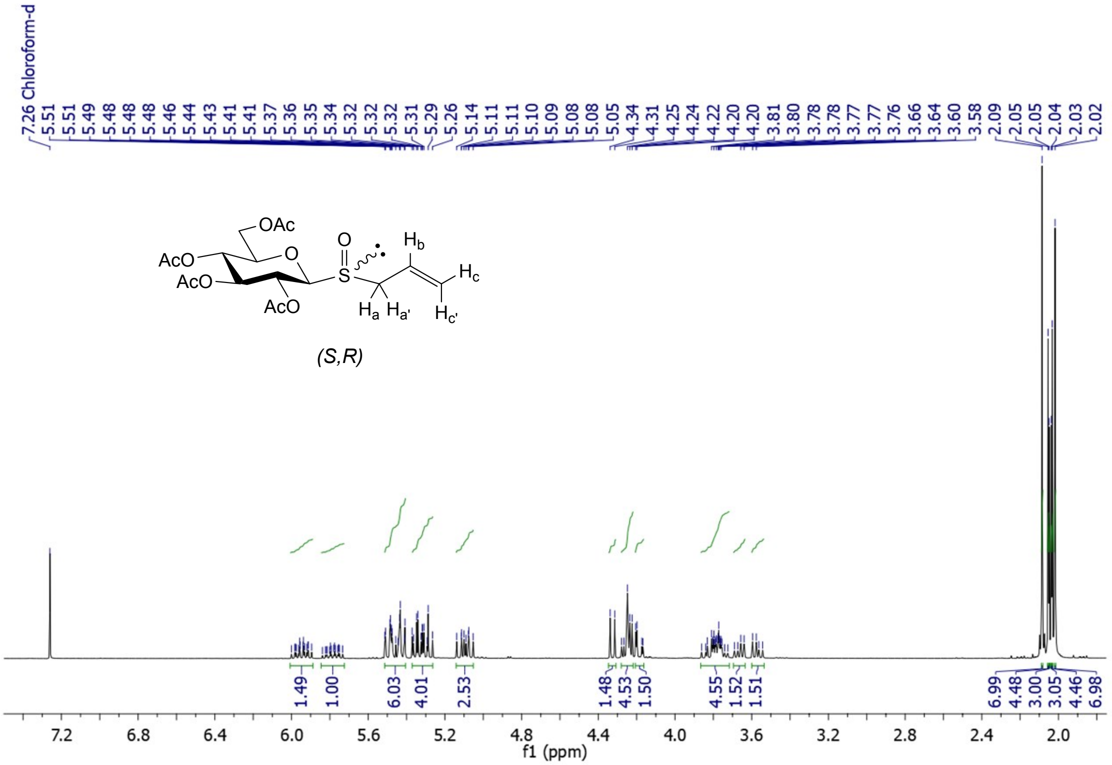

^{13}C NMR of compounds **4d*R*** and **4d*S***

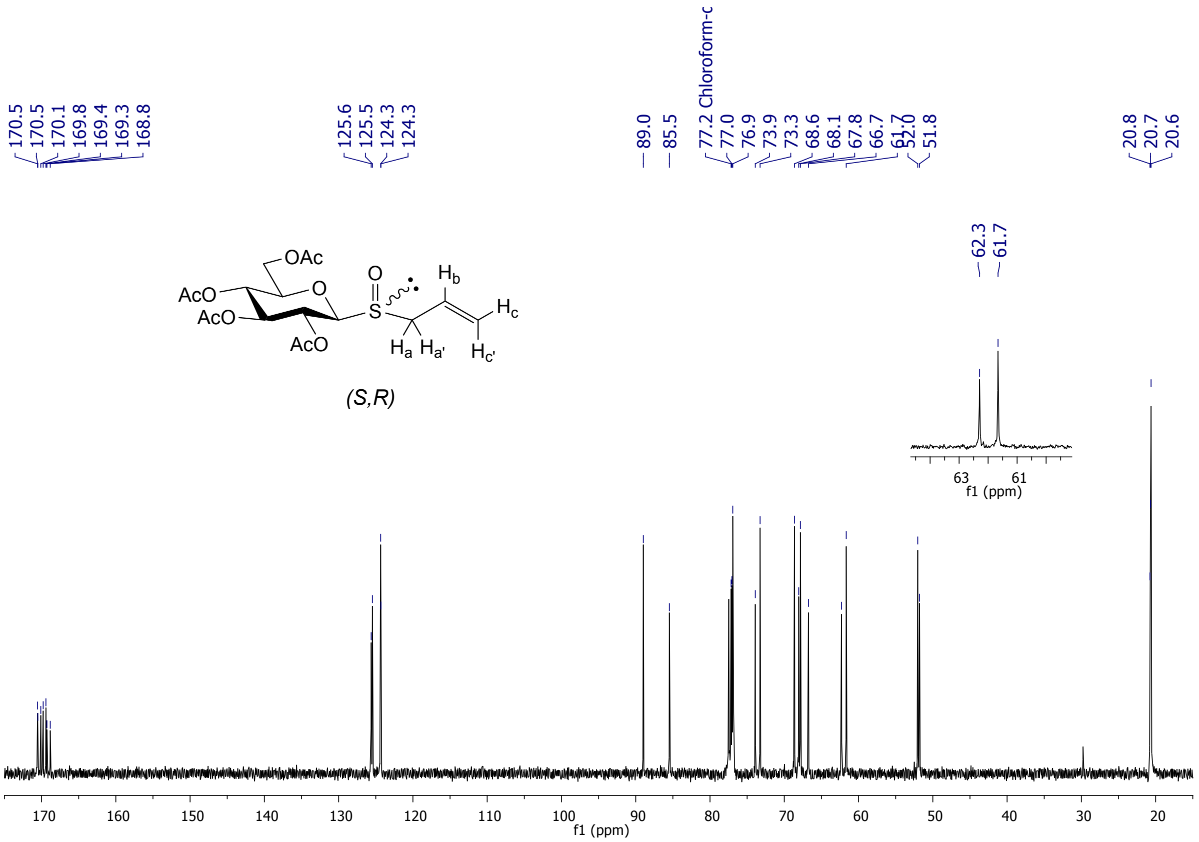

NOESY of compounds **4d*R*** and **4d*S***

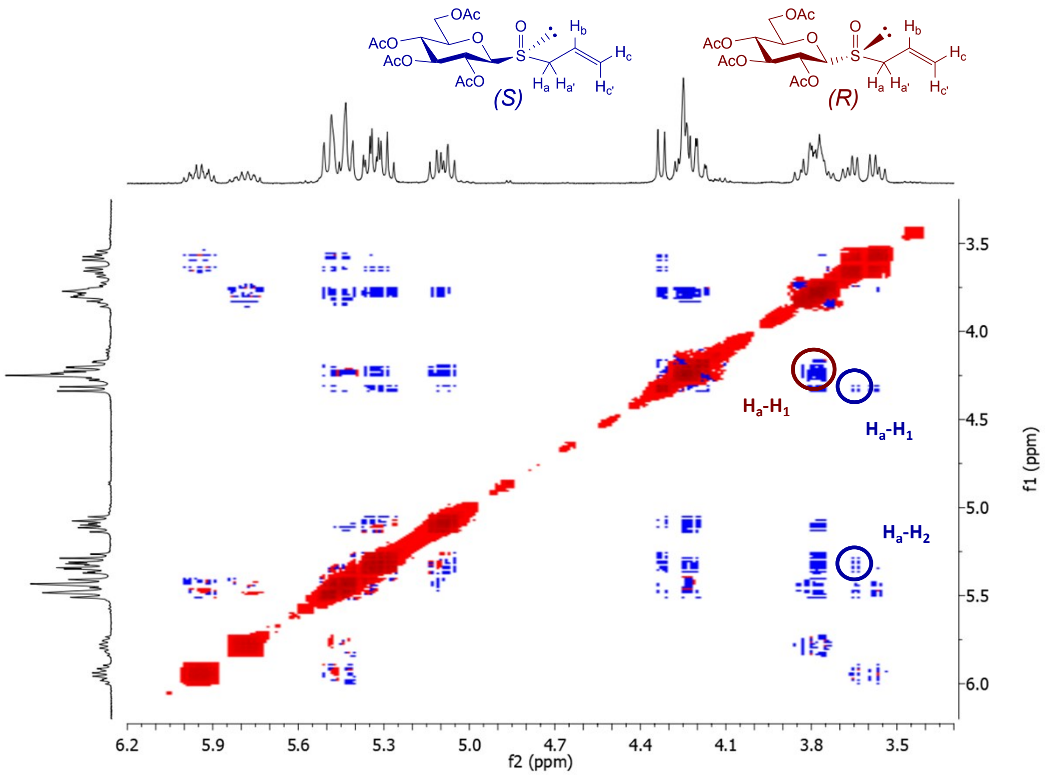

^{1}H NMR of compounds **4g*R*** and **4g*S***

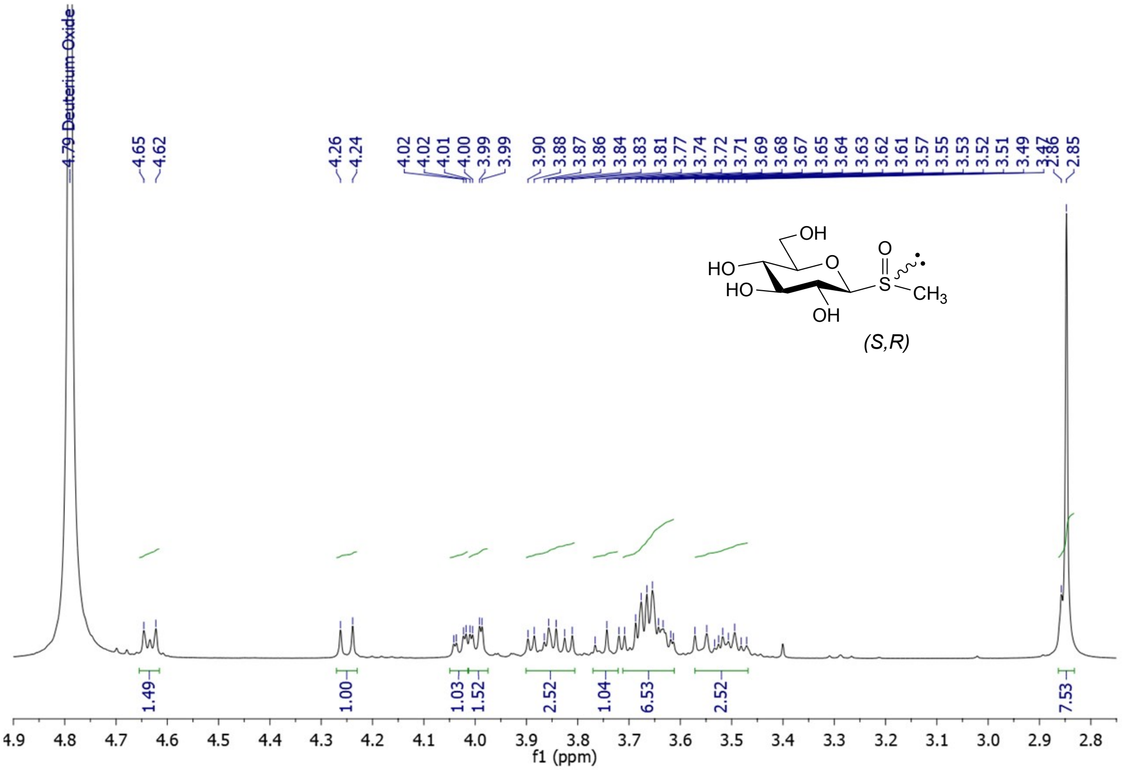

^{13}C NMR of compounds **4g*R*** and **4g*S***

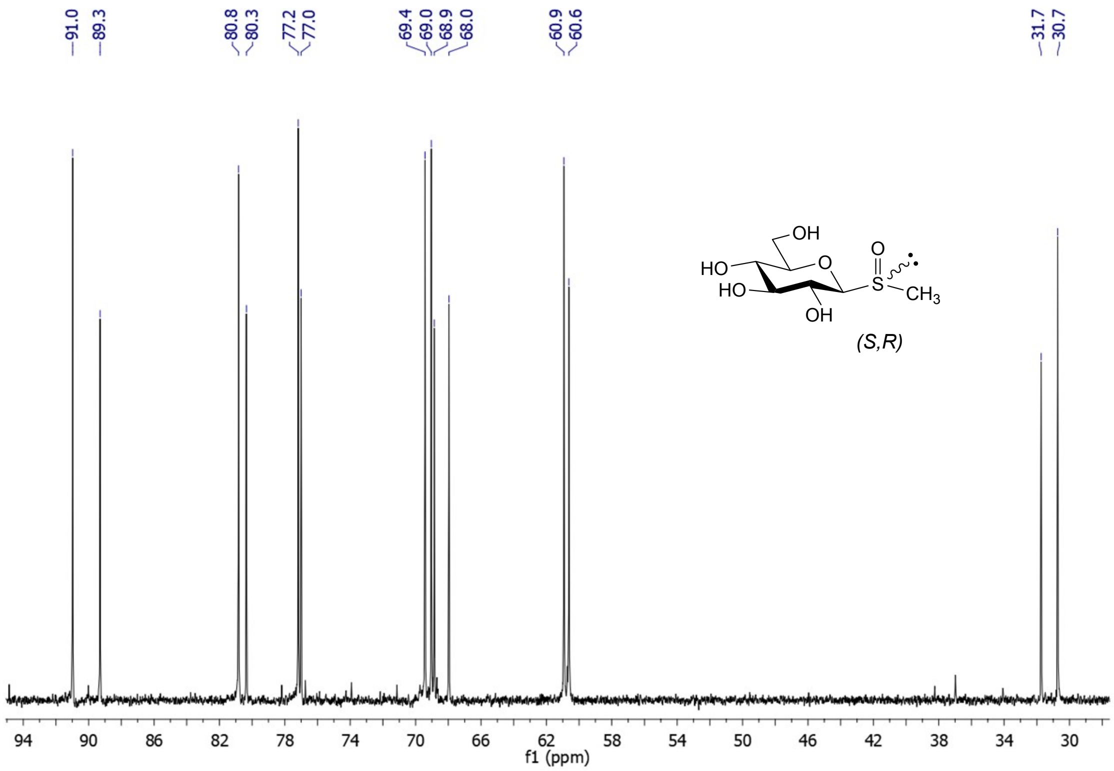

NOESY of compounds **4g*R*** and **4g*S***

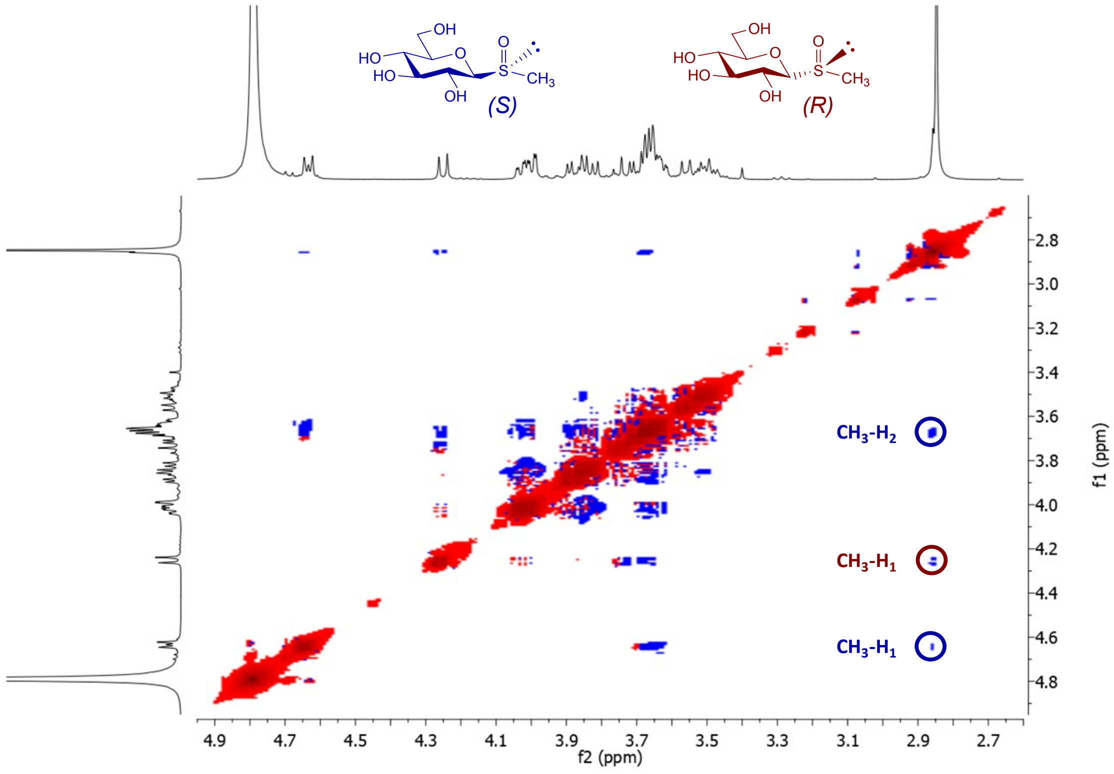

^{1}H NMR of compounds **4h*R*** and **4h*S***

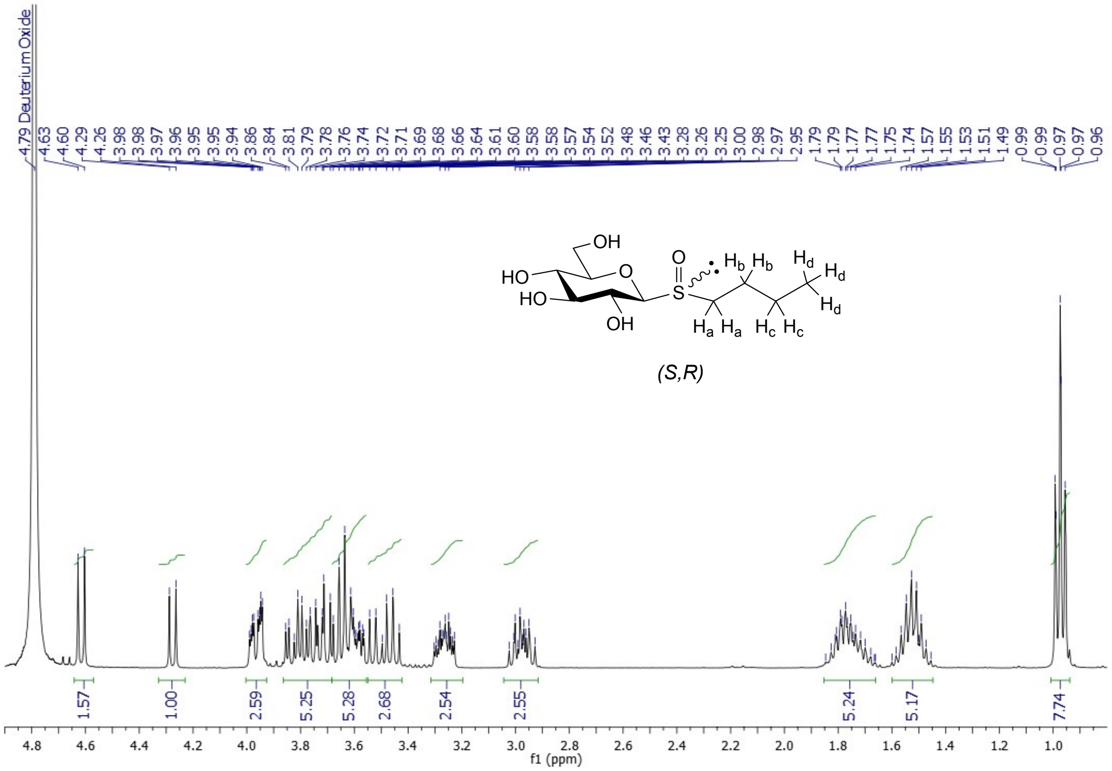

^{13}C NMR of compounds **4h*R*** and **4h*S***

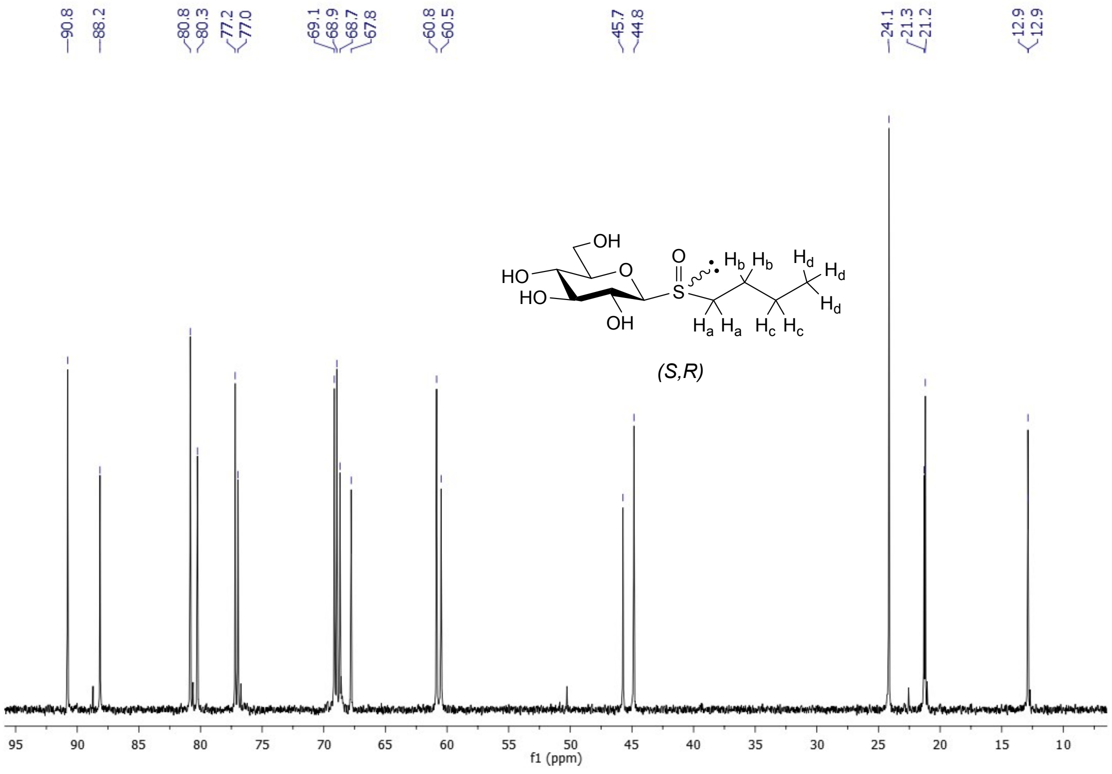

NOESY of compounds **4h*R*** and **4h*S***

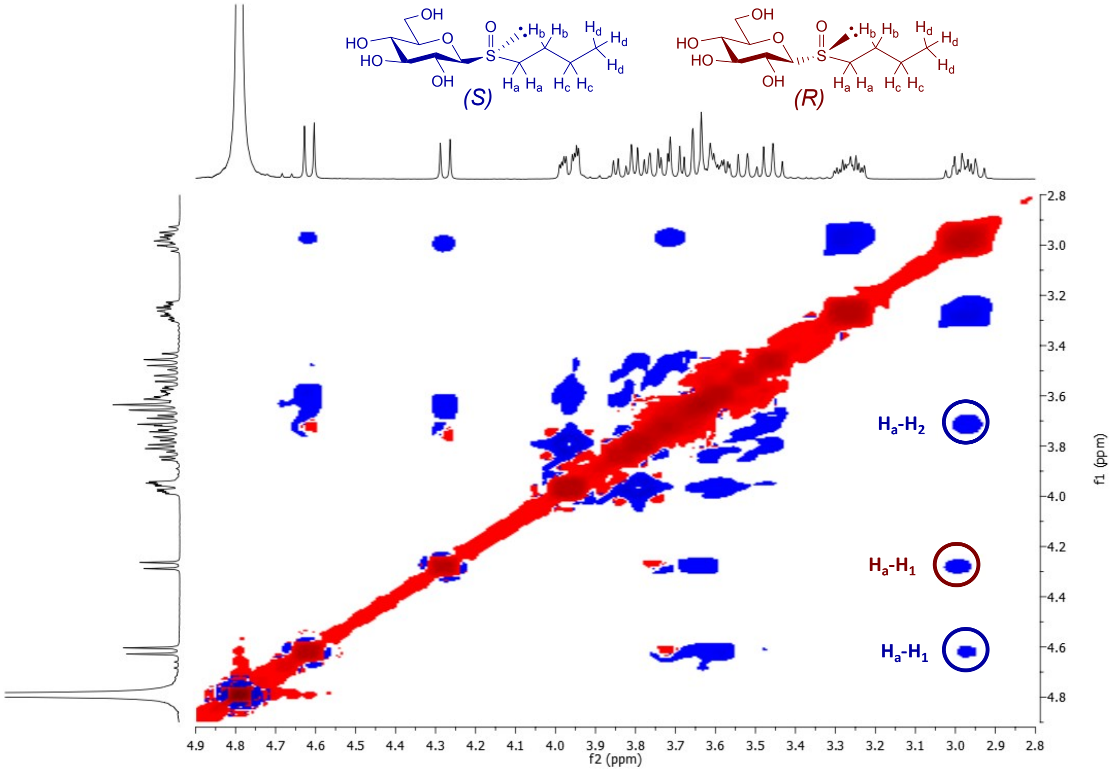

^{1}H NMR of compounds **4i*R*** and **4i*S***

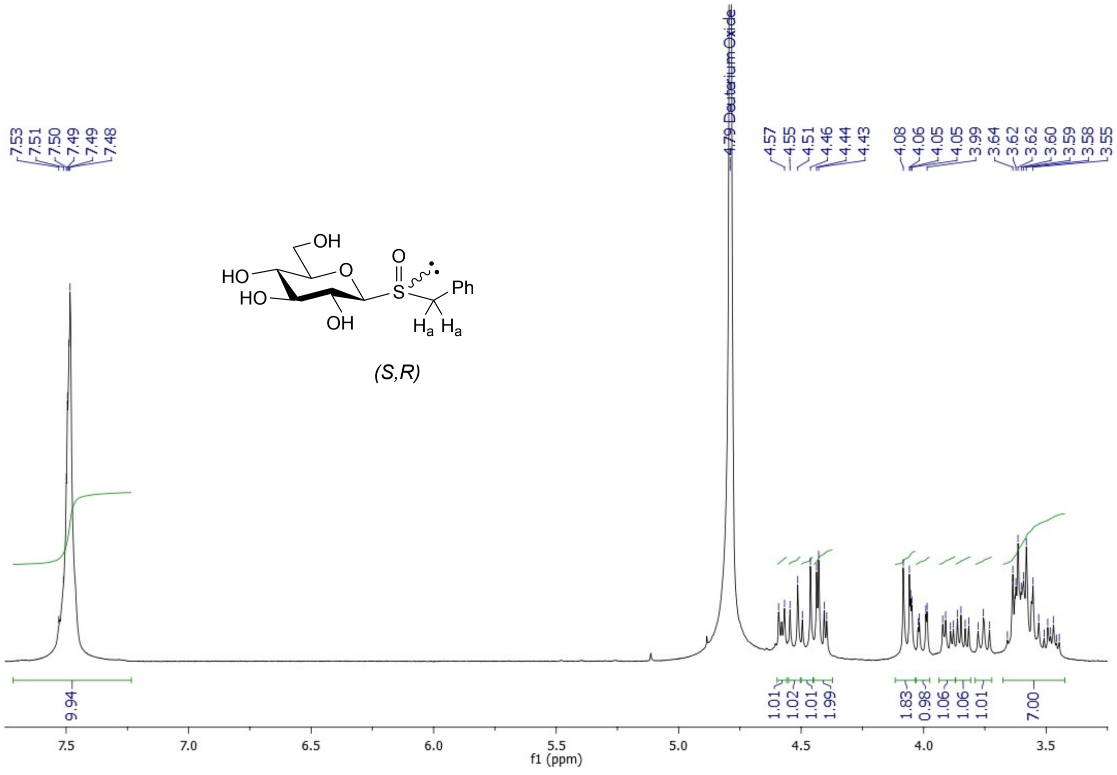

^{13}C NMR of compounds **4i*R*** and **4i*S***

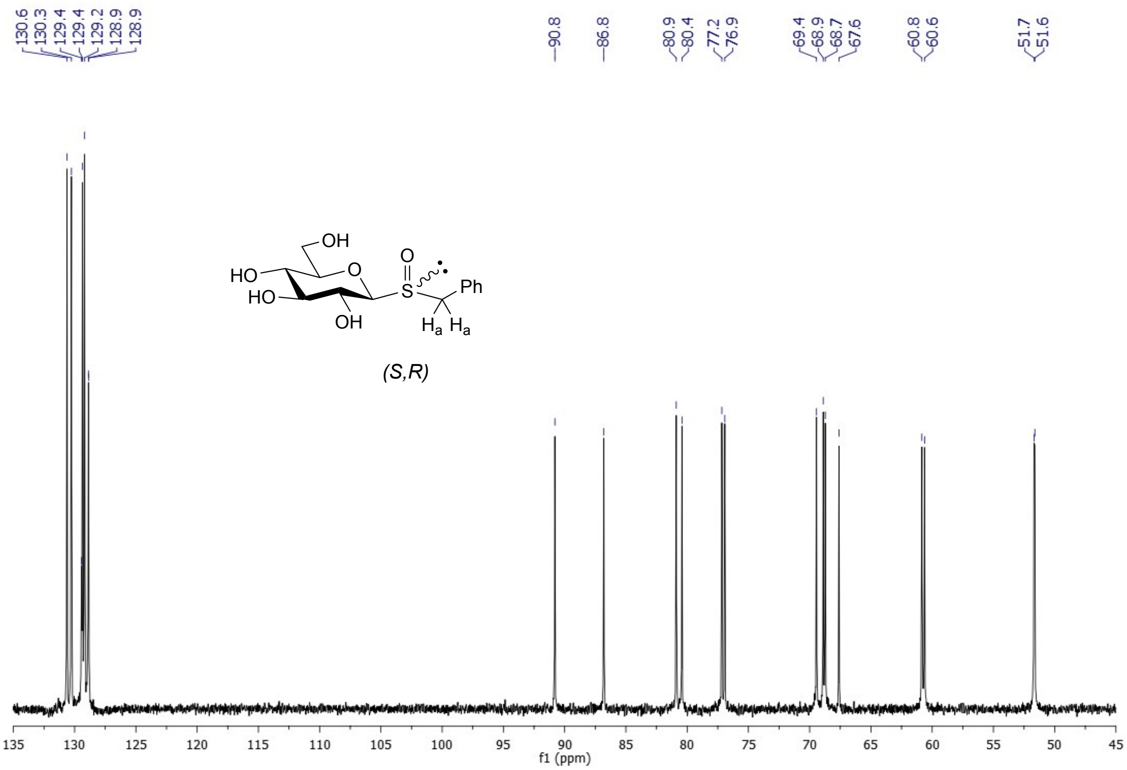

NOESY of compounds **4i*R*** and **4i*S***

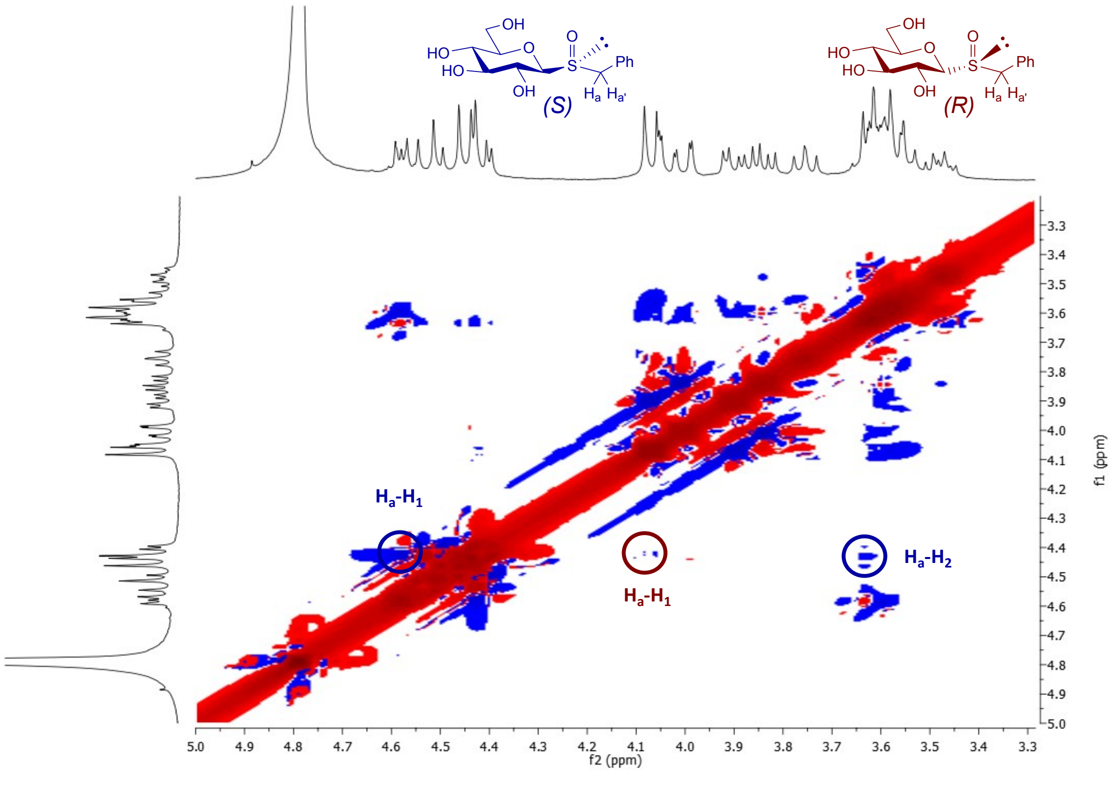

Comparison between ^{1}H spectra of penta-O-acetyl-D-glucopyranose and peracetylated fraction obtained after chromatographic column purification in the photooxidation reaction of **3j**

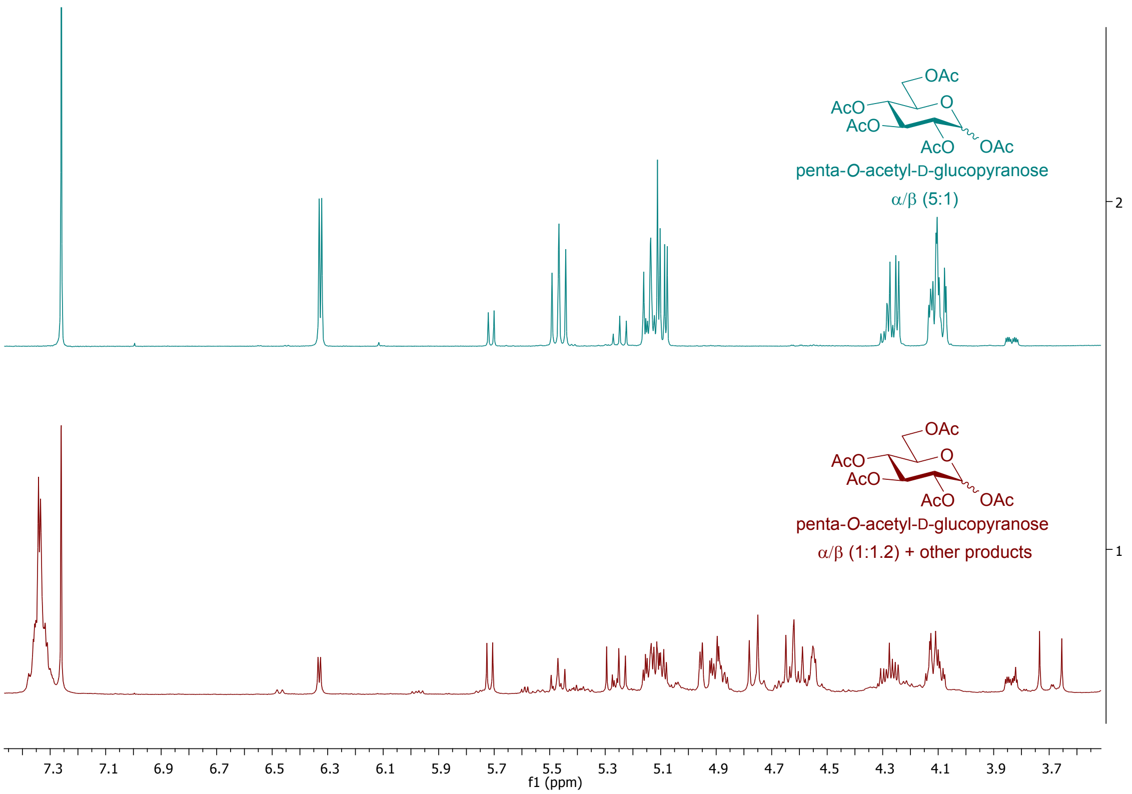

Supplement: RA-011-D0RA09534F-s001 [file RA-011-D0RA09534F-s001.pdf]
